# Direct Selection of Functional De Novo Macrocycles for Activation of *On‐Cellulo* Insulin Receptor

**DOI:** 10.1002/anie.202526008

**Published:** 2026-03-12

**Authors:** Yun‐Hsuan Kuo, Emiko Mihara, Junichi Takagi, Hiroaki Suga

**Affiliations:** ^1^ Department of Chemistry Graduate School of Science The University of Tokyo Tokyo Japan; ^2^ Laboratory of Protein Synthesis and Expression Institute for Protein Research Osaka University Suita Japan

**Keywords:** agonist discovery, cell‐based selection, mRNA display, nonproteinogenic amino acids, RaPID

## Abstract

The integration of mRNA display with genetic reprogramming, known as RaPID (Random non‐standard Peptides Integrated Discovery) system, has emerged as a promising platform for nonstandard peptide screening. The RaPID system relies on mRNA display of macrocyclic peptides which employs affinity‐based in vitro selection against an immobilized target protein on magnetic beads, followed by amplification of active species for repetitive rounds, enabling the enrichment of potent binders. Although the binding ability of such macrocyclic peptides is essential for its downstream functional activity, not all binders represent functionally active molecules. Here we have upgraded the RaPID system to directly discover functionally active species by extending to cellular screening, referred to as RaPID‐ExCells system. Using this system, we have discovered a potent de novo macrocycle (HL4) that specifically agonizes the insulin receptor (IR) on cells. Based on its sequence, we further performed the RaPID‐ExCells for deep mutational scanning of non‐proteinogenic amino acids without compromising the parental agonist activity and elucidated essential roles of residues in HL4. This work underscores the RaPID‐ExCells system as a unique platform for functional screening of macrocycles.

## Graphical abstract

A live‐cell screening platform, RaPID‐ExCells, integrates mRNA display‐derived macrocyclic peptide libraries with functional selection against native cell‐surface receptors. Application to the insulin receptor enabled de novo discovery of a macrocyclic agonist that activates endogenous signaling despite weak in vitro binding, highlighting the power of function‐first macrocycle selection.

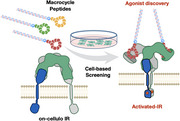

## 1 Introduction

Insulin receptors (IR), a class of receptor tyrosine kinases (RTKs), play pivotal roles in modulating various signaling cascades and mediating essential cellular functions, such as proliferation, survival, migration, and metabolism [1]. Dysfunctions in IR signaling give rise to metabolic diseases, including diabetes and cancers. Insulin stands out as one of the most significant hormones regulating IR activation and facilitating glucose entry into cells [2, 3, 4]. However, insulin administration has faced challenges such as insulin resistance [5], dosing complexities [6], and its propensity to aggregation [7]. Given the increasing prevalence of diabetes, the development of alternative molecules to insulin that are complement to existing therapeutic treatments is needed.

Recently, macrocyclic peptides are recognized as compelling therapeutic molecular modalities for addressing challenging protein targets [8, 9, 10]. Their inherent conformational restraint and structural preorganization mitigate entropic loss upon binding to proteins, resulting in enhanced binding affinity compared to their linear counterparts [11]. The macrocyclic scaffolds have shown a capacity to improve metabolic stability and membrane permeability [12, 13], endowing them with promising characteristics conducive to being potential drug candidates. As insulin alternatives, although insulin analogs [14, 15] and insulin mimetic peptides [16, 17, 18] have been developed to demonstrate the capability to regulate the IR function, in the realm of de novo discovery macrocyclic peptides that mimic the insulin function remain unexplored.

Over the past three decades, peptide display techniques have advanced substantially, offering diverse platforms for presenting peptide libraries and conducting high throughput screenings against specific protein targets [19, 20, 21, 22]. Among these methodologies, the RaPID (Random non‐standard Peptides Integrated Discovery) system, which integrates mRNA display with genetic code reprogramming enabled by flexizymes [23, 24, 25, 26, 27], has allowed us to screen trillions of unique macrocyclic peptides and uncover high affinity ligands against various challenging targets [28, 29, 30].

Despite these advantages, the RaPID system, like most in vitro display‐based selection platforms, fundamentally relies on affinity‐driven enrichment against purified or recombinant target proteins. As a consequence, enriched binders do not necessarily translate into biologically functional ligands, particularly for complex membrane proteins whose activity depends on native conformation, oligomerization state, or cellular context. Functional validation therefore typically requires individual chemical synthesis of candidate peptides followed by extensive cell‐based assays, a process that is labor‐intensive and significantly limits throughput.

In this work, we have upgraded the RaPID system to meet the above demand for finding functional species by extending to directly target a cellular protein receptor on live cells, referred to as RaPID‐ExCells (RaPID Extended to Cell‐based screening) system. By performing selection on intact cells, RaPID‐ExCells preserves the physiological conformation, oligomeric state, membrane environment, and downstream signaling competence of the target receptor, thereby shifting the selection pressure from binding affinity alone toward biological functionality. Using this platform, we conducted selection and enrichment of functional macrocyclic peptides against the IR, leading to the discovery of a potent de novo macrocyclic agonist without the need for extensive post‐selection functional triaging. Moreover, the integration of RaPID‐ExCells with deep mutational scanning (DMS) of non‐proteinogenic amino acids [31], providing deeper insights into the molecular determinants of receptor activation.

## 2 Results and Discussion

### 2.1 Macrocyclic Peptides Bound to IR‐Ectodomain Using the RaPID Platform

**FIGURE 1:**
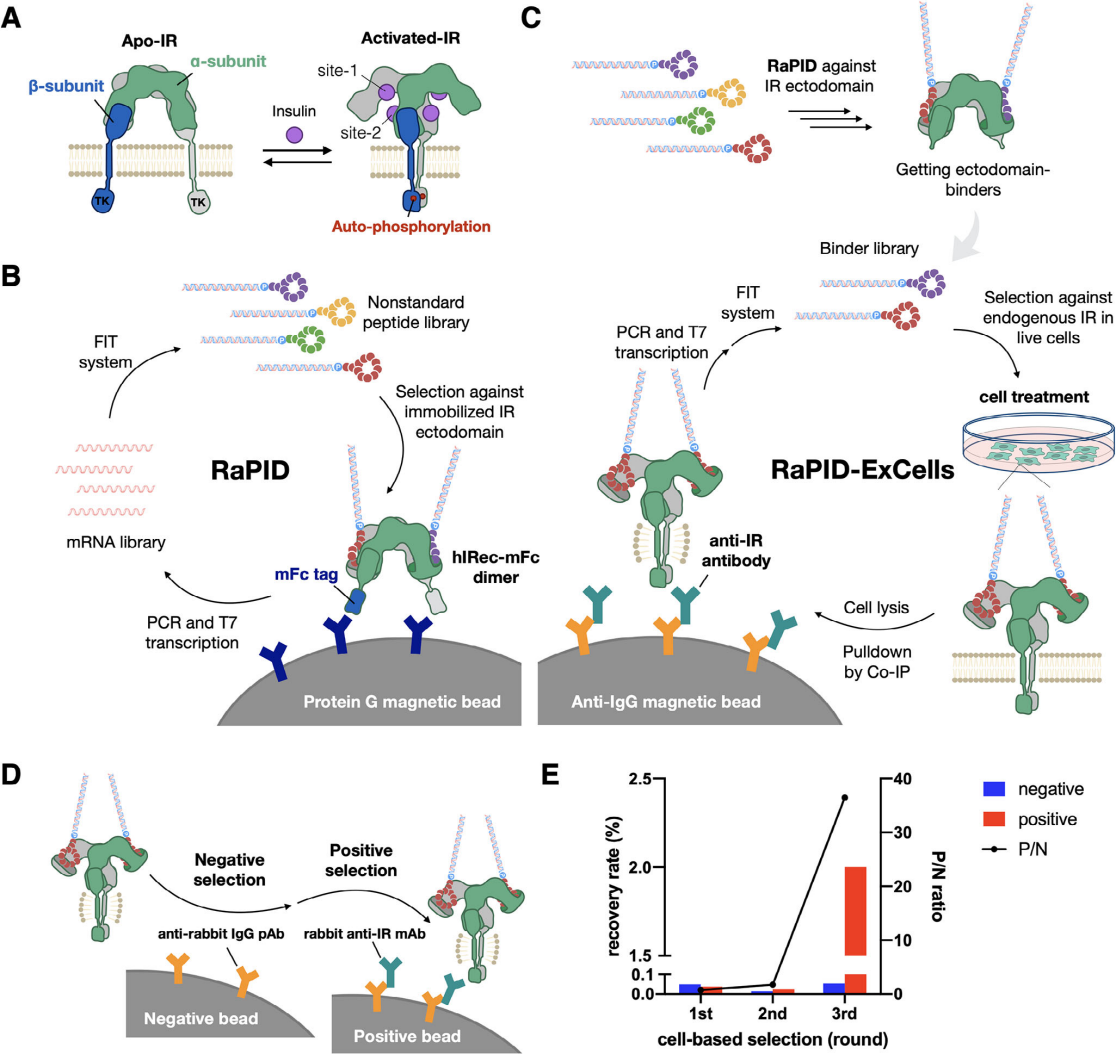
Activation of insulin receptor (IR) dimer and selection schemes used in this study. (A) Cartoon illustration of insulin‐induced conformational changes in the IR dimer and subsequent autophosphorylation of the tyrosine kinase (TK) domains. The IR dimer comprises two α‐ and two β‐subunits and contains four insulin‐binding sites. Upon insulin‐binding, the IR dimer undergoes a substantial conformational change and autophosphorylation in the TK domains. (B) Schematic representation of the RaPID system targeting IR. A nonstandard peptide library is constructed from an mRNA library using the flexible in vitro translation (FIT) system. The selection is performed against an mFc‐fused human IR ectodomain dimer (hIRec‐mFc) immobilized on Protein G magnetic beads. The cDNA‐peptide conjugates selected through this method are subsequently eluted from the beads and amplified for use in the next round of selections. (C) Schematic representation of the RaPID‐ExCells system. Peptides binding to endogenous IR on live cells are retrieved by co‐immunoprecipitation (Co‐IP) using a rabbit anti‐IR monoclonal antibody and sheep anti‐rabbit IgG beads. (D) Co‐IP workflow including negative and positive selection steps. In the negative selection, cell lysates are incubated with sheep anti‐rabbit IgG beads. In the positive selection, pre‐cleared lysates are incubated with a rabbit anti‐IR monoclonal antibody followed by co‐immunoprecipitation using sheep anti‐rabbit IgG beads. (E) Recovery rates and positive–negative (P/N) ratios of cDNA‐peptide conjugates obtained from positive and negative selections over three rounds using HEK293 cells.

The natural configuration of IR comprises two copies of extracellular ɑ‐subunits and β‐transmembrane subunits, forming a homodimer architecture (Figure 1A) [32, 33, 34]. Within the intracellular domain of IR, inactive tyrosine kinase (TK) domains undergo activation upon insulin binding. In the extracellular domains, maximally four insulins bind to an IR dimer at two distinct sites, termed site‐1 and site‐2, each playing distinct roles in regulating the conformational structure and activity of the IR [35, 36]. Insulin‐activation at the extracellular domains induces a conformational change in the IR, bringing the kinase domains into contact and initiating auto‐phosphorylation of specific Tyr residues in the intracellular domains. From a structural perspective, our aim was to discover macrocyclic peptides capable of stimulating IR activity. To specifically target the extracellular ectodomain, we employed IR‐ectodomain homodimers as targets and the RaPID platform to screen trillions of macrocyclic peptides.

The RaPID platform could facilitate the discovery of binders through affinity interactions with protein targets (Figure 1B). To enhance the likelihood of identifying binders that specifically target the extracellular region of IR, we engineered an IR homodimer composed of two mFc‐fused human IR ectodomains (hIRec‐mFc). The monomeric construction of hIRec‐mFc includes an insulin‐binding α‐subunit, a truncated β‐subunit, and a monomeric Fc (mFc) sequence for immobilization onto protein G magnetic beads (Figure S1) [37, 38]. The purity of expressed dimers was confirmed through SDS‐PAGE gel analysis (Figure S2A). To minimize the capture of non‐specific binders during selection, it was crucial to saturate the loading of hIRec‐mFc dimers onto the protein G magnetic beads. By gradually increasing the amounts of the capture beads, we demonstrated that protein loading reached saturation at a ratio of 0.625 pmol IR per µL of beads (Figure S2B), which was used throughout the RaPID selection campaign.

We used our standard macrocyclic library [39, 40], where the translation was initiated with *N‐*chloroacetyl‐L‐tyrosine (ClAc‐L‐Y), elongated with 11–15 random proteinogenic amino acids encoded by NNK codons (N = G, C, A, or U; K = G or U), a cysteine residue for spontaneous thioether‐closing cyclization with the N‐terminal ClAc group, followed by a GSGSGS linker as a spacer in the methionine‐deficient FIT system (Figures S3 and S4A). This library consisting of greater than a trillion (>10^12^) of unique members of macrocycles was displayed on mRNA via puromycin introduced at the 3′‐end of mRNA. After the reverse transcription of mRNAs to form macrocycle–mRNA/cDNA conjugates, it was subjected to three rounds of negative selections against Fc protein and naked magnetic beads, and one round of positive selection against the aforementioned beads immobilized with hIRec‐mFc dimers (Figure S4B). In total, we carried out five rounds of campaign and observed significant enrichment of peptides after three rounds (Figure S4C).

**FIGURE 2:**
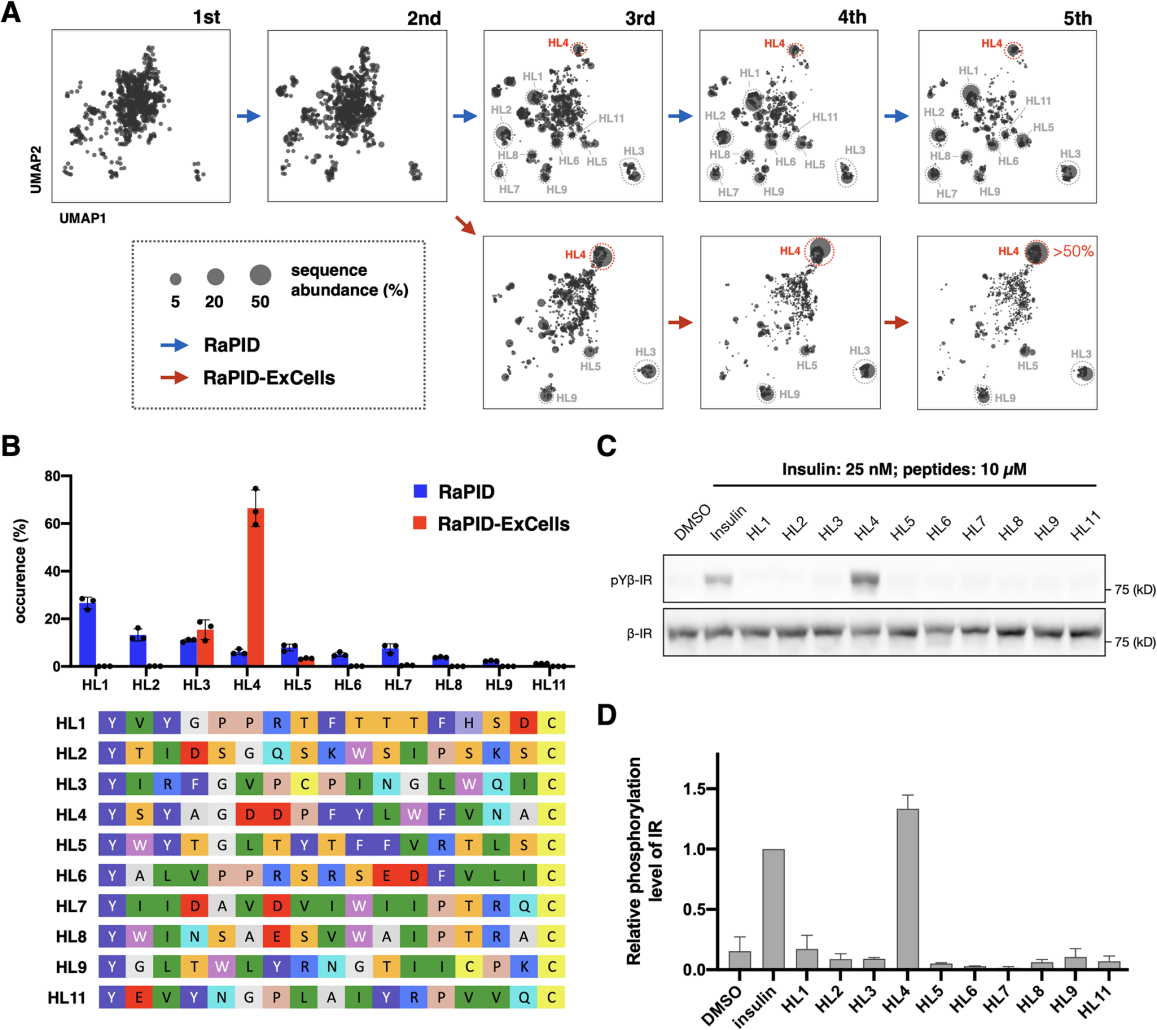
Peptide‐enriched evolution in RaPID and RaPID‐ExCells campaigns. (A) Evolution of selected peptide sequences analyzed by uniform manifold approximation and projection (UMAP). Each circle represents a unique peptide sequence, and the circle size reflects its relative abundance. Sequence clusters corresponding to HL1–HL11 are indicated, with the HL4 cluster highlighted in red. (B) Comparison of sequence occurrence frequencies in the fifth round of RaPID and RaPID‐ExCells selections. The relative abundances of HL1–HL11 peptide sequences are shown. Data represent mean ± SD from three independent experiments. (C) Autophosphorylation of the insulin receptor (pYβ‐IR) induced by peptides (10 µM) for 5 min. Total IR β‐subunit (β‐IR) levels are shown as a loading control. (D) Quantification of western blot data shown in (C). Phosphorylation levels were normalized to those induced by insulin. Data represent mean ± SD from three independent experiments.

A higher recovery rate of DNA from positive selection was observed compared to that of negative selection, indicating most selected binders were specifically bound to the hIRec‐mFc. The sequence information of the enriched peptides was subsequently identified by next generation sequencing (NGS). We identified more than 35 independent peptide candidates with occurrences larger than 0.1% in abundance (Figure S5). We focused on the top 11 peptides based on uniqueness of sequences, referred to as HL1–11, as candidates that potentially activate IR (Figure 2B). These macrocycles thus represent promising IR‐ectodomain‐binding scaffolds discovered directly through affinity‐driven selection.

### 2.2 RaPID Extending to Cell‐Based Screening, RaPID‐ExCells

It has been shown that insulin binding to IR could induce a notable conformational change, in which its substantial movements across the multiple domains would alter the shape of the IR dimer from an inverted‐V‐shape to a T‐shape (Figure 1A). Since we executed the affinity‐based selection to the ectodomain of IR (Figure 1B), the selected binders were certainly specific to the ectodomain (i.e., they do not bind to the intracellular domain of IR). However, it is uncertain that they could bind the appropriate binding site capable of inducing the conformational alteration to the “T‐shaped” IR dimer essential for the cellular agonist activity. We usually conduct chemical synthesis and purification of all candidates identified by the RaPID selection, and then individually screen their functional activity. However, in the current study we gave a thought to upgrading the RaID to RaPID‐ExCells system where the enriched naïve library was applied to “*on‐cellulo*” selection against IR expressed on cells (Figure 1C).

In the RaPID‐ExCells system, IRs on human embryonic kidney cells (HEK 293) served as the native IR target. We took the ectodomain‐binding yet diverse species enriched in round 2 in the RaPID selection, and treated them with live HEK 293 cells (Figure 1C). The cells then underwent four washes to eliminate unbound conjugates, followed by cell lysis using a nonionic detergent. Previous studies have shown that nonionic detergents can maintain the native conformation, dimeric state, and biological activity of IR and related RTKs after cell lysis, thereby allowing ligand–receptor interactions to remain intact under such conditions [41]. The lysate was then treated with a rabbit anti‐IR monoclonal antibody (mAb), and the ternary complex of peptide•IR•mAb was captured by sheep anti‐rabbit IgG polyclonal antibody (pAb) beads (Figure 1D). The co‐precipitated peptide–mRNA/cDNA species were recovered and then PCR‐amplified for the next round of selection.

To determine whether binders can be identified through endogenous IR, we evaluated the enrichment of the peptide–cDNAs determined by qPCR quantification for the positive and negative selections (Figure 1E). A significant enrichment was observed after three rounds of the ExCells campaign against *on‐cellulo* IR conformational species. We observed more than 2% of the recovery rate in the third round of the positive selection whereas only 0.05% for the negative selection. This notable increase in P/N (positive–negative) ratio indicates that more peptides bound to the *on‐cellulo* IR and were co‐immunoprecipitated by the anti‐IR mAb. Next, we compared the NGS data of peptides enriched in the RaPID‐ExCells experiment with those in the earlier RaPID experiment in order to gain more insights into the outcomes.

### 2.3 Macrocycles Enriched by the RaPID‐ExCells

The NGS data of the enriched peptides were analyzed using UMAP (Uniform Manifold Approximation and Projection) to visualize the sequences in a two‐dimensional (2D) representation according to their sequence‐based chemical similarity [42, 43, 44, 45, 46]. Enriched sequences from the respective rounds of the RaPID and RaPID‐ExCells campaigns were analyzed to depict 2D UMAPs (Figure 2A), in which circles and sizes represented unique sequences and their frequencies of occurrence. The peptide sequences with high structural similarities were positioned closer to each other and classified into clusters. Notably, we found that HL1–HL9 peptides were distinctly separated in the UMAP plots and classified into distinct clusters (Figure S6), indicating that they are chemically independent of one another.

Next, we utilized the UMAP plots to illustrate the peptide‐enriched evolution during the selection campaigns (Figure 2A). This 2D visualization provided a comparable overview between the evolution patterns of RaPID and RaPID‐ExCells campaigns. The RaPID library from round 2 was subjected in parallel to both RaPID‐ExCells and RaPID selections, and the respective populations were compared. The RaPID UMAPs revealed that the sequence clusters of HL1–HL9 were enriched during the selection (Figure S6, left panel). On the other hand, some clusters were deselected in the RaPID‐ExCells UMAPs, settling into four clusters of HL3–5 and HL9 (Figures 2B and S6, right panel). Notably, the HL4 cluster was more enriched than other clusters in round 5, reaching about 50% in the total population (Figure S6, right panel). In the RaPID UMAPs, the HL4 cluster also survived during the selection but remained a relatively minor cluster (Figure S6, left panel).

To characterize the functions of these enriched peptides, we employed immunoblotting to evaluate cellular IR activation. The respective peptides were synthesized using solid‐phase peptide synthesis (SPPS), and their purity and mass were determined by LC/MS (Figures S7 and S8). The phosphorylation level at the TK domain of IR, denoted as pYβ‐IR, was detected and quantified. The blotting of IR β‐subunits, denoted as β‐IR, served as an internal control to normalize the total amount of extracted IR. As depicted in Figure 1C,D, only HL4 exhibited the expected agonist activity, triggering the auto‐phosphorylation of IR. In contrast, other peptides showed no discernible agonist activity. Clearly, the RaPID‐ExCells effectively enriched HL4 (Figure 2A,B), indicating that it is advantageous for identifying functional molecules.

To quantitatively characterize the binding affinity of the discovered peptides, HL1–6, against IR, we performed surface plasmon resonance using hIRec‐mFc (Figure S9). The dissociation constants (*K*_D_) of peptides were evaluated from association kinetic (*k*_on_) and dissociation (*k*_off_) rate constants. We found that all peptides, except for HL2 (*K*_D_ = 1.7 µM), exhibited potent binding affinities where *K*_D_ values were determined to be in a 2–130 nM range (Figure S9). Interestingly, HL4 exhibited a rather weak affinity (*K*_D_ = 132 nM) to the ectodomain on the sensor chip. Given that HL4 was most strongly enriched by the RaPID‐ExCells campaign, HL4 should have a higher affinity to the native ectodomain of the cellular IR rather than its in vitro counterpart.

In this study, we introduce RaPID‐ExCells, an advanced selection strategy that integrates the molecular diversity and genetic programmability of the RaPID system with cell‐based target screening, thereby directly enriching macrocyclic peptides based on their functional engagement with targets in a native cellular context. Compared to conventional RaPID selections performed against isolated recombinant proteins, RaPID‐ExCells enables the identification of biologically active macrocyclic agonists, as exemplified by the discovery of HL4. Notably, HL4 exhibited weaker binding affinity toward the IR ectodomain relative to other enriched macrocycles (HL1–3), yet demonstrated superior binding and functional activity toward IR expressed on living cells. This apparent discrepancy underscores a fundamental limitation of affinity‐only, in vitro selections and highlights a key advantage of RaPID‐ExCells: by employing intact cells, the selection process preserves the native membrane environment, post‐translational modifications, and the dimeric state of the IR, which is essential for receptor activation.

### 2.4 Comparative Analysis of Agonist Activity in HL4 and Its Dimer

**FIGURE 3:**
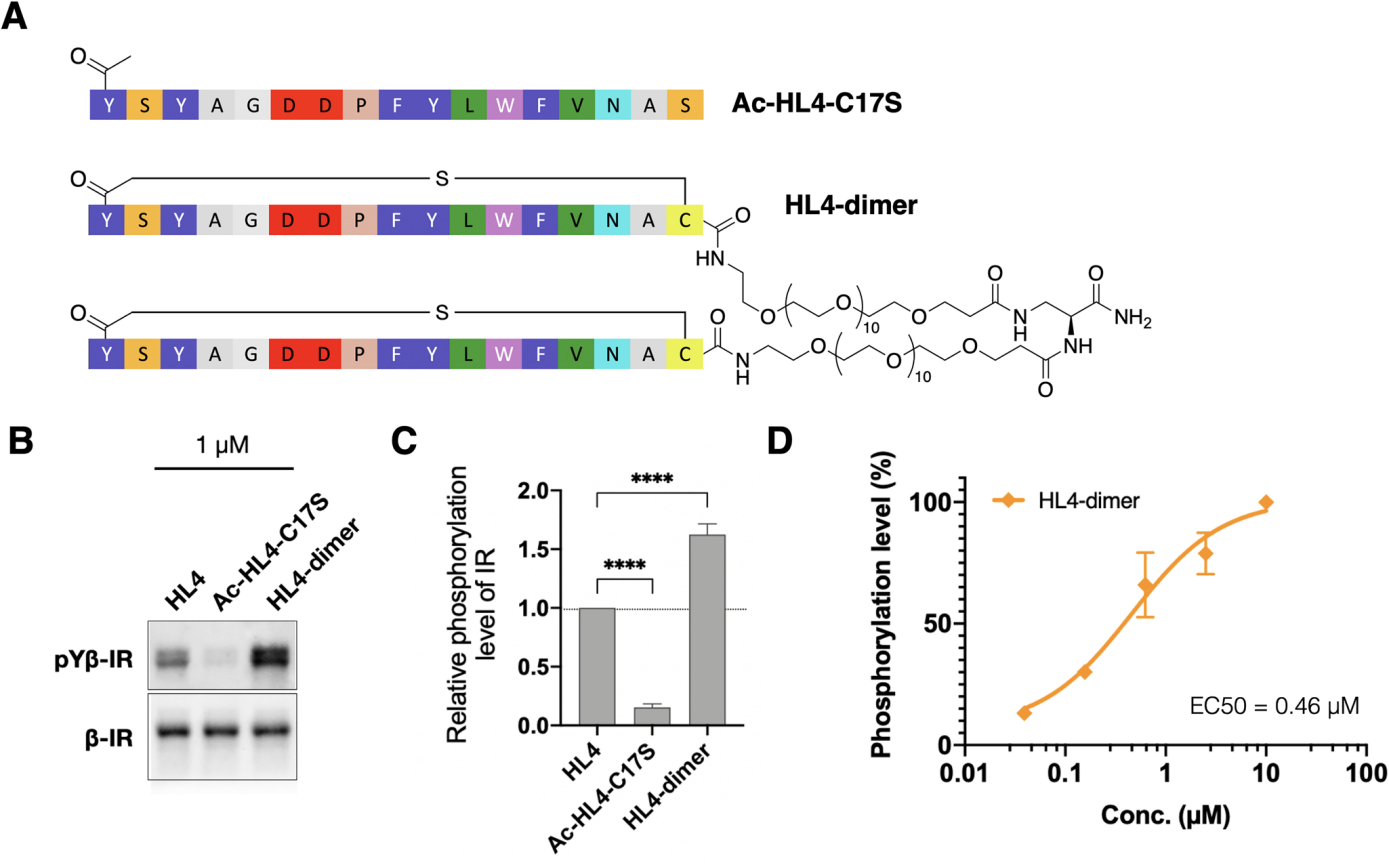
Agonist activity of HL4, HL4‐dimer, and linear Ac‐HL4‐C17S. (A) Chemical structures of the linear HL4 variant Ac‐HL4‐C17S and the HL4 homodimer. The homodimer consists of two HL4 monomers linked via PEG11 spacers to an L‐Dap core. (B) Autophosphorylation of the pYβ‐IR induced by peptides (1 µM) for 5 min. The linear Ac‐HL4‐C17S peptide shows no detectable agonist activity. (C) Quantification of western blot data shown in (B). Data represent mean ± SD from three independent biological replicates. Statistical significance was evaluated using a two‐tailed unpaired Student's *t*‐test (****p < 0.0001). (D) Dose–response curve of peptide‐induced IR phosphorylation. Data represent mean ± SD from three independent experiments.

We identified HL4 as a potent activator of IR autophosphorylation. Here we sought to further enhance the therapeutic potency of this peptide. Given that IR naturally forms dimers on the cell membranes, we hypothesized that dimerization of HL4 could further improve its agonist activity. To synthesize a homodimeric form of this peptide, we conjugated an _L_‐Dap amino acid with two PEG11 linkers and connected them to two HL4 monomers (Figure 3A). The introduction of polyethylene glycol (PEG) to peptides gives chemical perspective to improve their physicochemical properties, for example, aqueous solubility. To verify the importance of the macrocyclic structure, we also synthesized a linear HL4 mutant, referred to as Ac‐HL4‐C17S (Figure 3A), where the C17 residue was mutated to S17.

To characterize their agonist activities, we performed immunoblotting to evaluate the agonist activities of these peptides (Figure 3B). As expected, HL4 exhibited agonist activity, whereas the acyclic Ac‐HL4‐C17S showed no detectable activity, clearly indicating that the macrocyclic structure plays a pivotal role in activating IR (Figure 3B,C). Remarkably, the HL4‐dimer showed 2.5‐fold enhanced agonist activity compared to HL4 itself at a concentration of 1 µM. We carried out a dose‐dependent experiment with a series of HL4‐dimer concentrations (Figures 3D and S10), leading us to determine EC_50_ to be 460 nM.

We also delved into the metabolic stability of three peptides: Ac‐HL4‐C17S, HL4, and HL4‐dimer. These peptides underwent incubation with human serum at 37°C for defined time intervals, after which their quantities were quantified using LC/MS against an internal control peptide. Our findings revealed distinct half‐lives for each peptide: The half‐lives of Ac‐HL4‐C17S, HL4, and HL4‐dimer were 12, 20, and 51 h, respectively (Figure S11A). Collectively, these results indicate that dimerization markedly enhances the serum stability of HL4, supporting its suitability for cellular and functional studies and providing a favorable basis for its observed *on‐cellulo* agonist activity.

To further assess potential off‐target effects, particularly binding to IR‐like receptors, we evaluated the receptor selectivity of HL4 using a RTK phosphorylation array. HEK293FT cells were treated with HL4‐dimer (400 nM), insulin (4 nM), or vehicle control, and RTK activation profiles were analyzed using the Proteome Profiler Human Phospho‐RTK Array Kit (Figure S11B). While insulin induced robust phosphorylation of the IR as expected, HL4‐dimer selectively activated IR without significant phosphorylation of other closely related or structurally similar RTKs, including IGF‐1R and other kinases represented on the array.

The enhanced agonist activity observed for the HL4 dimer compared to its monomeric counterpart further suggests that RaPID‐ExCells may preferentially enrich ligands that exploit higher‐order receptor organization on the cell surface. At present, it remains unclear whether the increased activity arises from receptor dimer stabilization, cooperative engagement of two receptor protomers, or a more general avidity effect. Nevertheless, these findings illustrate how cell‐based selection can reveal functional modalities that are inaccessible to conventional in vitro RaPID screens, which are blind to receptor architecture and signaling dynamics.

### 2.5 Nonproteinogenic Deep Mutational Scanning of HL4 Using RaPID‐ExCells

**FIGURE 4:**
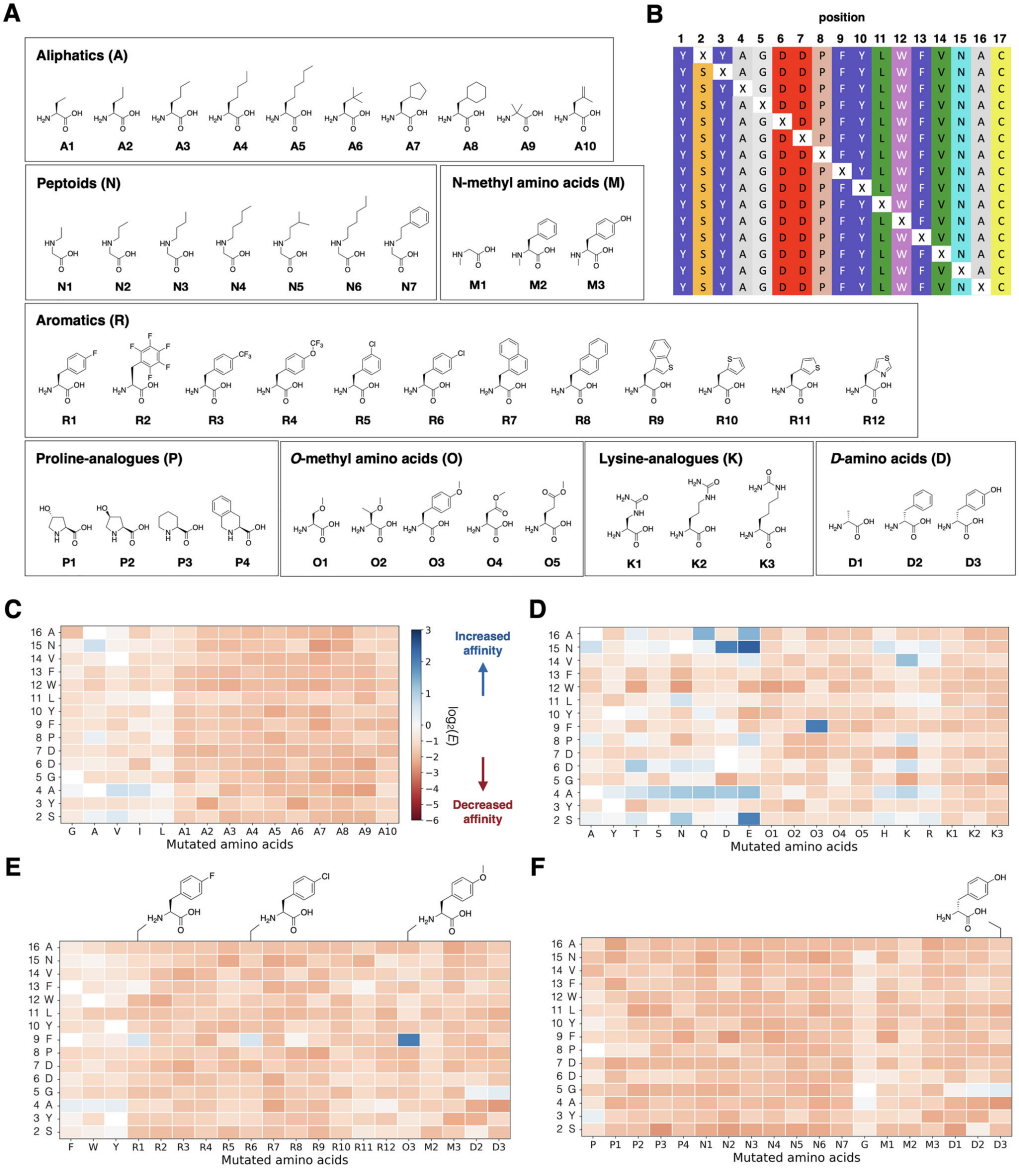
Deep mutational scanning of HL4 using RaPID‐ExCells. (A) Amino acids employed in the scanning campaign, including proteinogenic and non‐proteinogenic variants. “X” denotes the scanned position, which was substituted with 19 proteinogenic and 47 non‐proteinogenic amino acids. Enrichment scores are visualized as heatmaps and grouped into the following categories: (B) aliphatic amino acids, (C) polar amino acids, (D) aromatic amino acids, and (E) peptoids, proline analogues, and _D_‐amino acids. Matrix values represent log_2_‐transformed enrichment scores [log_2_(E)] for amino acid substitutions at each position within the core HL4 sequence. Peptides with log_2_(E) > 0 (blue) indicate increased enrichment relative to the parent sequence.

To gain deeper insights into the critical HL4 residues responsible for the binding of HL4 to cellular IR, we conducted DMS using RaPID‐ExCells [31]. We chose 47 nonproteinogenic amino acids (npAAs) as well as 19 proteinogenic amino acids (pAAs, except for Met) for the DMS campaign, so that a total of 66 amino acids were adopted for the entire campaign (Figure 4A). The 47 npAAs were *N*‐methyl amino acids (M1–M3), _D_‐amino acids (D1–D3), aliphatic npAAs (A1–A10), N‐alkyl‐alanines (peptoids, N1–N7), *O*‐methyl amino acids (O1–O5), proline analogues (P1–P4), guanidino lysine derivatives (K1–K3), and aromatic npAAs (R1–R12) [47, 48, 49, 50, 51, 52, 53, 54]. We reprogrammed the methionine elongator AUG codon for the assignment of an npAA or pAA at position 2–16 in the mRNA templates (Figures S12–S15). Since we assembled 47 npAAs and 19 pAAs at once into the library of mRNAs, we specified each mRNA encoding the respective npAA/pAA by a unique barcode sequence tailored to its 3′‐end for deep‐sequencing identification and thus each mRNA can be distinguished (Table S3).

The DMS campaigns yielded fruitful information on mutational tolerance at each residue of HL4 to pAAs and npAAs (Figures 4B–E and S16). A Log2‐transformed enrichment score [Log2(E)] was calculated for each mutant, with Log2(E) > 0 indicating increased affinity compared to the parent sequence; Log2(E) = 0 indicates identical affinity; otherwise, the affinity is decreased. Mutation of each HL4 residue to aliphatic npAAs and pAAs showed a silent or slightly negative impact on the recovery rate of HL4 toward cellular IR (Figure 4C). On the other hand, mutation to polar pAAs or *O*‐methyl amino acids increased the recovery rate at several positions (Figure 4D). Particularly, S1E, F8O3, and N15D/E mutations significantly enhanced the recovery rate of HL4 to cellular IR, suggesting that these mutants might have increased binding affinity. Most aromatic npAAs and pAAs mutations also resulted in a silent or negative effect, but F8R1 and F8R6 gave slightly positive effect (Figure 4E). Mutagenesis of HL4 with _D_‐amino acids, *N*‐methyl amino acids, peptoids, and proline analogues generally decreased the recovery rate, except for mutation of G5 to D1–D3 that exhibited a slight increase in the recovery rate (Figure 4E).

### 2.6 HL4 Mutants Containing npAAs Showed Increased Agonist Activity

**FIGURE 5:**
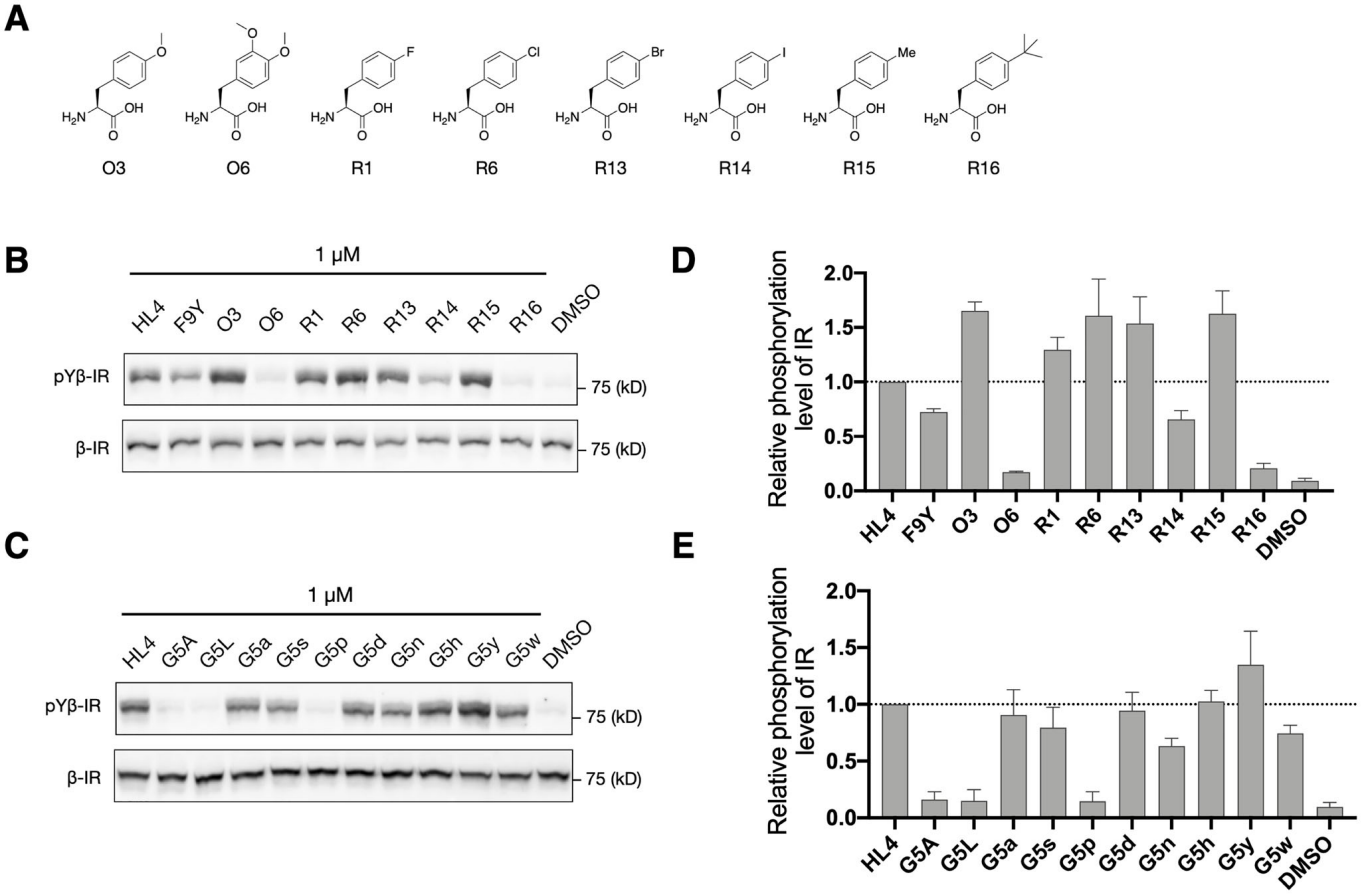
Agonist activity of HL4 mutants incorporating non‐proteinogenic amino acids. (A) Substitution of non‐proteinogenic aromatic amino acids for phenylalanine at position 9 (F9) in HL4. (B) Autophosphorylation of the pYβ‐IR induced by HL4 mutants containing non‐proteinogenic aromatic amino acids at position 9. (C) Autophosphorylation of the pYβ‐IR induced by HL4 mutants incorporating D‐amino acids at glycine position 5 (G5). Lowercase letters denote glycine substitutions with the corresponding D‐amino acids. (D, E) Quantification of western blot data shown in (B) and (C). Data represent mean ± SD from three independent experiments.

Inspired by the results of the DMS study, we identified several positions possibly amenable to the introduction of npAAs. Thus, we synthesized and purified distinct HL4 mutants incorporating npAAs (Figures S17–S22). Aromatic npAAs (O6 and R13–16, Figure 5A) were introduced to the phenylalanine at position 9 (F9) in HL4, and these mutants were tested for their agonist activity (Figure 5A). The F9‐to‐tyrosine mutant (F9Y), which reduced the recovery rate in the DMS study (Figure 4D), was a less active agonist than the wild‐type HL4. On the other hand, its O3, R1, and R6 mutants, which showed the increased recovery rates in the DMS study, exhibited agonist activity approximately 1.5‐fold higher than wildtype HL4 (Figure 5B,C). Thus, the DMS study identified the mutants that exhibit higher agonist activity most likely reflecting the improved affinity to cellular IR. We also synthesized mutants which were not included in the DMS study, O6 and R13‐R16, and evaluated their agonist activity. Interestingly, an additional *m*‐methoxy group in O3 drastically reduces the agonist activity. Another large negative effect was observed for *p*‐iodo and *p*‐tert‐butyl groups. These substituents made F9 bulky, which probably created an adverse steric clash to the binding site. On the other hand, other *p*‐substituents with R6, R13, and R15 had a positive impact on increasing the agonist activity by 1.5‐fold, indicating that an additional modest steric interaction is favorable to the *p*‐position of F9.

The DMS study showed G5 mutation to any _L_‐amino acids (Figure 4C–F) was incompatible, but that with _D_‐amino acids were tolerated despite a modest effect (Figure 4E,F). Therefore, we mutated G5 with more diverse _D_‐amino acids, for example, _D_‐Ala mutation, referred to as G5a (Figure 5C,E). First, we synthesized two representative mutants where G5 was mutated to _L_‐alanine and _L_‐leucine (G5A and G5L, respectively), confirming that these macrocycles lost wild‐type agonist activity (Figure 5C,E). We also confirmed that the G5a (_D_‐Ala) mutant retained the agonist activity comparable to wildtype HL4 as predicted by DMS. Most G5x (x = s, d, n, and w) showed 0.6–0.9‐fold decreases in agonist activity; except that G5h retained the wildtype level, whereas G5p resulted in a completely loss of activity. Most importantly, the G5y mutant exhibited 1.2‐fold higher agonist activity. These data suggest that achiral G5 would play a role in making a turn structure which could be substituted with appropriate _D_‐amino acids, such as _D_‐tyrosine.

The DMS study also included the scanning of *N*‐methyl amino acids (M1–M3), proline analogs (P1–P4), and *N‐*alkylglycines (N1–N7), but none of these made a positive impact on the recovery rates. However, because incorporation of these amino acids could potentially enhance both metabolic stability and oral bioavailability of HL4, we evaluated their agonist activity. This also allows us to validate the reliability of data generated by DMS study for future experiments. We thus prepared the series of mutants containing such secondary amino acids, represented by F9M2, Y10M3, and F13M2 which were included in the DMS study (Figures S21 and S22). Moreover, we synthesized some additional mutants with corresponding *N*‐methyl amino acids, such as S2^Me^S, A4^Me^A, and six more such substituents (Figures S21 and S22). Again, the DMS data matched the loss of their agonist activity for F9M2, Y10M3, and F13M2 (Figure S23). Among those which were not included in the DMS study, S2^Me^S, Y3^Me^Y, A4^Me^A, and D6^Me^D showed about 30%–50% of wildtype activity, whereas others were nearly inactive (Figure S23). Since the retention of agonist activity was observed in the mutants in the upstream positions close to *N*‐terminus, possibly indicating that this region is exposed to solvent and tolerate the mutations of *N*‐methylation on peptide bond as far as the same side‐chain was kept. Since G5 might act as a turn‐motif in macrocycle, this upstream region may be applicable to future investigation for shortening the peptide sequence and optimizing the structure for potential oral availability.

### 2.7 **Structure**‐**Activity Relationships of npAA‐Incorporated HL4 Mutants**

The application of RaPID‐ExCells combined with DMS and npAA incorporation enabled a comprehensive exploration of sequence‐activity relationships, facilitating the identification of optimized substitutions to enhance peptide agonist activity. Consistent with DMS results, we identified several npAA substitutions that improved agonist function. Notably, the introduction of moderately bulky groups (O3, R1, R3, R13, and R15) at the para‐position of phenylalanine (F9) enhanced agonist activity, suggesting that steric interactions at this site can be leveraged to improve binding or signaling. In contrast, meta‐substitutions (O3) or larger para‐substituents (R14, R16) severely diminished activity, indicating that the F9 side chain is deeply buried within a constrained binding pocket with limited tolerance for bulkier or alternatively positioned modifications.

Strikingly, our DMS revealed that glycine at position 5 (G5) exhibits broad tolerance for diverse _D_‐amino acids, with minimal impact on agonist activity. Except for G5p, which exhibited a completely loss of activity, other mutants modestly reduced function, underscoring its stereospecific role. This observation aligns with glycine's unique properties as the only achiral, conformationally flexible proteinogenic amino acid. The permissible incorporation of _D_‐amino acids likely preserves backbone geometry by adopting compatible φ/ψ backbone torsion angles, whereas _L_‐amino acids would introduce destabilizing steric clashes or torsional strain. Beyond functional optimization, the strategic use of _D_‐amino acids at flexible positions like G5, _D_‐tyrosine in this case, could also enhance proteolytic stability and modestly improve agonist activity, a critical consideration for therapeutic peptide development.

### 2.8 Essential Role of the FYXWF Motif in Insulin Receptor Activation

Previous phage display studies have identified insulin‐mimetic peptides capable of binding the IR and inducing autophosphorylation [17, 55]. Among these, a 31‐mer peptide S597 contains a critical FYXWF motif, which is indispensable for agonist activity. Cryo‐EM structural studies revealed that this motif interacts with the L1 domain of IR (Figure S24B), mimicking the binding mode of the native α‐CT segment (Figure S24C) [56, 57]. Intriguingly, our de novo macrocyclic peptide HL4 also shares an FYLWF motif (residues 9–13; Figure S24A). Through comprehensive region‐focused scanning (Figure S25A) and functional assays (Figure S25B–E), we demonstrated the absolute requirement of the motif's aromatic residues for HL4 activity, as their substitution abolished agonist function while non‐aromatic mutations showed minimal effects. This strongly suggests HL4 engages the L1 domain through a similar FYXWF‐mediated mechanism. A striking functional dichotomy emerged when comparing HL4 with a linear S597‐derived peptide, referred to as the 16‐mer S597‐component‐1: While this linear peptide fails to activate IR without its full‐length context, the 17‐mer HL4 peptide autonomously triggers receptor activation. This stark contrast highlights the crucial advantage conferred by HL4's macrocyclic architecture in achieving potent agonist activity. These findings not only validate previous structural insights but also provide new molecular understanding of macrocyclic peptide agonists, underscoring their therapeutic potential through structural optimization.

Our analysis further identified several positions amenable to *N*‐methyl amino acid incorporation, predominantly at non‐aromatic sites distal from the essential FYXWF motif embedded in residues 9–13 (Figure S24D). The preserved agonist activity in N‐terminal proximal mutants suggests this region is solvent‐exposed and tolerant of peptide bond *N*‐methylation when side‐chain identity is maintained. Given G5's potential role as a turn‐inducing residue in the macrocycle, this flexible N‐terminal region presents an attractive target for future peptide minimization studies aimed at developing shorter, more drug‐like analogs with potential oral bioavailability. The synergistic application of RaPID‐ExCells with npAA scanning provides a powerful platform for semi‐rational design of the next‐generation peptide therapeutics.

## 3 Conclusion

In conclusion, this work introduces an innovative selection strategy by integrating RaPID and cell‐based targeting approaches, referred to as RaPID‐ExCells. Targeting receptors on the cell surface offers a native environment conducive to the discovery of functional binders. The cellular assay highlighted HL4 as a unique agonist peptide, exclusively identified through RaPID‐ExCells. This finding underscores the significance of the cell‐based targeting approach in identifying peptides with specific cellular activities. Identified diverse agonists incorporating npAAs were discovered by employing DMS via RaPID‐ExCells. We demonstrated the introduction of npAAs into the aromatic motif leads to an improvement in agonist activity. Moreover, the use of RaPID‐ExCells not only for RaPID selection but also for DMS campaigns provides a rapid approach to identifying functional macrocycles targeting various cellular receptors or membrane proteins on cells. This strategy provides a powerful platform for identifying functional macrocyclic ligands that engage cellular receptors in their native membrane environment, enabling direct selection based on cellular activity rather than isolated biochemical interactions.

## Conflicts of Interest

The authors declare no conflicts of interest.

## Supporting information




**Supporting File 1**: anie71738‐sup‐0001‐SuppMat.docx.

## Data Availability

The data that support the findings of this study are available in the Supporting Information of this article.

## References

[1] R. A. Haeusler , T. E. McGraw , and D. Accili , “Biochemical and Cellular Properties of Insulin Receptor Signalling,” Nature Reviews Molecular Cell Biology 19 (2018): 31–44, 10.1038/nrm.2017.89. 28974775 PMC5894887

[2] M. C. Petersen and G. I. Shulman , “Mechanisms of Insulin Action and Insulin Resistance,” Physiological Reviews 98 (2018): 2133–2223, 10.1152/physrev.00063.2017. 30067154 PMC6170977

[3] M. Li , X. Chi , Y. Wang , S. Setrerrahmane , W. Xie , and H. Xu , “Trends in Insulin Resistance: Insights Into Mechanisms and Therapeutic Strategy,” Signal Transduction and Targeted Therapy 7 (2022): 216, 10.1038/s41392-022-01073-0. 35794109 PMC9259665

[4] J. P. Mayer , F. Zhang , and R. D. DiMarchi , “Insulin Structure and Function,” Peptide Science 88 (2007): 687–713, 10.1002/bip.20734. 17410596

[5] J. Boucher , A. Kleinridders , and C. R. Kahn , “Insulin Receptor Signaling in Normal and Insulin‐Resistant States,” Cold Spring Harbor Perspectives in Biology 6 (2014): a009191, 10.1101/cshperspect.a009191. 24384568 PMC3941218

[6] B. A. Perkins , J. L. Sherr , and C. Mathieu , “Type 1 Diabetes Glycemic Management: Insulin Therapy, Glucose Monitoring, and Automation,” Science 373 (2021): 522–527, 10.1126/science.abg4502. 34326234

[7] Q. Hua and M. A. Weiss , “Mechanism of Insulin Fibrillation,” Journal of Biological Chemistry 279 (2004): 21449–21460, 10.1074/jbc.M314141200. 14988398

[8] X. Li , T. W. Craven , and P. M. Levine , “Cyclic Peptide Screening Methods for Preclinical Drug Discovery,” Journal of Medicinal Chemistry 65 (2022): 11913–11926, 10.1021/acs.jmedchem.2c01077. 36074956

[9] A. A. Vinogradov , Y. Yin , and H. Suga , “Macrocyclic Peptides as Drug Candidates: Recent Progress and Remaining Challenges,” Journal of the American Chemical Society 141 (2019): 4167–4181, 10.1021/jacs.8b13178. 30768253

[10] F. Giordanetto and J. Kihlberg , “Macrocyclic Drugs and Clinical Candidates: What Can Medicinal Chemists Learn From Their Properties?,” Journal of Medicinal Chemistry 57 (2014): 278–295, 10.1021/jm400887j. 24044773

[11] J. E. DeLorbe , J. H. Clements , B. B. Whiddon , and S. F. Martin , “Thermodynamic and Structural Effects of Macrocyclic Constraints in Protein−Ligand Interactions,” ACS Medicinal Chemistry Letters 1 (2010): 448–452. 21116482 10.1021/ml100142y PMC2992351

[12] P. Matsson , B. C. Doak , B. Over , and J. Kihlberg , “Cell Permeability Beyond the Rule of 5,” Advanced Drug Delivery Reviews 101 (2016): 42–61, 10.1016/j.addr.2016.03.013. 27067608

[13] P. G. Dougherty , A. Sahni , and D. Pei , “Understanding Cell Penetration of Cyclic Peptides,” Chemical Reviews 119 (2019): 10241–10287, 10.1021/acs.chemrev.9b00008. 31083977 PMC6739158

[14] X. Xiong , J. G. Menting , M. M. Disotuar , et al., “A Structurally Minimized Yet Fully Active Insulin Based on Cone‐Snail Venom Insulin Principles,” Nature structural & molecular biology 27 (2020): 615–624, 10.1038/s41594-020-0430-8. PMC7374640 32483339

[15] I. B. Hirsch , “Insulin Analogues,” New England Journal of Medicine 352 (2005): 174–183, 10.1056/NEJMra040832. 15647580

[16] M. Lubos , J. Pícha , I. Selicharová , et al., “Modulation of the Antagonistic Properties of an Insulin Mimetic Peptide by Disulfide Bridge Modifications,” Journal of Peptide Science 29 (2023): e3478, 10.1002/psc.3478. 36633503 PMC10909431

[17] J. Park , J. Li , J. P. Mayer , et al., “Activation of the Insulin Receptor by an Insulin Mimetic Peptide,” Nature Communications 13 (2022): 5594, 10.1038/s41467-022-33274-0. PMC9508239 36151101

[18] L. Schäffer , R. E. Brissette , J. C. Spetzler , et al., “Assembly of High‐Affinity Insulin Receptor Agonists and Antagonists From Peptide Building Blocks,” Proceedings of the National Academy of Sciences of the United States of America 100 (2003): 4435–4439, 10.1073/pnas.0830026100. 12684539 PMC153573

[19] C. Villequey , S. S. Zurmühl , C. N. Cramer , et al., “An Efficient mRNA Display Protocol Yields Potent Bicyclic Peptide Inhibitors for FGFR3c: Outperforming Linear and Monocyclic Formats in Affinity and Stability,” Chemical Science 15 (2024): 6122–6129, 10.1039/D3SC04763F. 38665530 PMC11040643

[20] S. G. Reyes , Y. Kuruma , M. Fujimi , et al., “PURE mRNA Display and cDNA Display Provide Rapid Detection of Core Epitope Motif via High‐Throughput Sequencing,” Biotechnology and Bioengineering 118 (2021): 1702–1715, 10.1002/bit.27696. 33501662

[21] G. Kamalinia , B. J. Grindel , T. T. Takahashi , S. W. Millward , and R. W. Roberts , “Directing Evolution of Novel Ligands by mRNA Display,” Chemical Society Reviews 50 (2021): 9055–9103, 10.1039/D1CS00160D. 34165126 PMC8725378

[22] M. S. Newton , Y. Cabezas‐Perusse , C. L. Tong , and B. Seelig , “ *In Vitro* Selection of Peptides and Proteins—Advantages of mRNA Display,” ACS Synthetic Biology 9 (2020): 181–190, 10.1021/acssynbio.9b00419. 31891492 PMC8203280

[23] Y. Goto and H. Suga , “The RaPID Platform for the Discovery of Pseudo‐Natural Macrocyclic Peptides,” Accounts of Chemical Research 54 (2021): 3604–3617, 10.1021/acs.accounts.1c00391. 34505781

[24] S. Wang , E. C. Woods , J. Jo , et al., “An mRNA Display Approach for Covalent Targeting of a *Staphylococcus aureus* Virulence Factor,” Journal of the American Chemical Society 147 (2025): 8312–8325, 10.1021/jacs.4c15713. 40013487 PMC12118155

[25] T. Katoh , Y. Goto , and H. Suga , Pept. Macrocycles, ed. M.B. Coppock and A.J. Winton (Springer US, 2022) 247–259.

[26] Y. Goto , T. Katoh , and H. Suga , “Flexizymes for Genetic Code Reprogramming,” Nature Protocols 6 (2011): 779–790, 10.1038/nprot.2011.331. 21637198

[27] S. E. Iskandar , L. F. Chiou , T. M. Leisner , et al., “Identification of Covalent Cyclic Peptide Inhibitors in mRNA Display,” Journal of the American Chemical Society 145 (2023): 15065–15070, 10.1021/jacs.3c04833. 37395736 PMC11246720

[28] S. A. Dai , Q. Hu , R. Gao , et al., “State‐Selective Modulation of Heterotrimeric Gαs Signaling With Macrocyclic Peptides,” Cell 185 (2022): 3950–3965.e25, 10.1016/j.cell.2022.09.019. 36170854 PMC9747239

[29] J. M. Rogers , M. Nawatha , B. Lemma , et al., “ *In Vivo* Modulation of Ubiquitin Chains by *N*‐Methylated Non‐Proteinogenic Cyclic Peptides,” RSC Chemical Biology 2 (2021): 513–522, 10.1039/D0CB00179A. 34179781 PMC8232551

[30] K. Colas , D. Bindl , and H. Suga , “Selection of Nucleotide‐Encoded Mass Libraries of Macrocyclic Peptides for Inaccessible Drug Targets,” Chemical Reviews 124 (2024): 12213–12241, 10.1021/acs.chemrev.4c00422. 39451037 PMC11565579

[31] J. M. Rogers , T. Passioura , and H. Suga , “Nonproteinogenic Deep Mutational Scanning of Linear and Cyclic Peptides,” Proceedings of the National Academy of Sciences of the United States of America 115 (2018): 10959–10964, 10.1073/pnas.1809901115. 30301798 PMC6205457

[32] E. Uchikawa , E. Choi , G. Shang , H. Yu , and X. Bai , “Activation Mechanism of the Insulin Receptor Revealed by Cryo‐EM Structure of the Fully Liganded Receptor–Ligand Complex,” Elife 8 (2019): e48630, 10.7554/eLife.48630. 31436533 PMC6721835

[33] T. Gutmann , K. H. Kim , M. Grzybek , T. Walz , and Ü. Coskun , “Visualization of Ligand‐Induced Transmembrane Signaling in the Full‐Length Human Insulin Receptor,” Journal of Cell Biology 217 (2018): 1643–1649, 10.1083/jcb.201711047. 29453311 PMC5940312

[34] M. C. Lawrence , “Understanding Insulin and Its Receptor From Their Three‐Dimensional Structures,” Molecular Metabolism 52 (2021): 101255, 10.1016/j.molmet.2021.101255. 33992784 PMC8513149

[35] X. Xiong , A. Blakely , J. H. Kim , et al., “Symmetric and Asymmetric Receptor Conformation Continuum Induced by a New Insulin,” Nature Chemical Biology 18 (2022): 511–519, 10.1038/s41589-022-00981-0. 35289328 PMC9248236

[36] J. Li , J. Park , J. P. Mayer , et al., “Synergistic Activation of the Insulin Receptor via Two Distinct Sites,” Nature Structural & Molecular Biology 29 (2022): 357–368, 10.1038/s41594-022-00750-6. PMC9115778 35361965

[37] C. Wang , Y. Wu , L. Wang , et al., “Engineered Soluble Monomeric IgG1 Fc With Significantly Decreased Non‐Specific Binding,” Frontiers in immunology 8 (2017): 1545, 10.3389/fimmu.2017.01545. 29181008 PMC5693891

[38] N. Yasui , Y. Kitago , A. Beppu , et al., “Functional Importance of Covalent Homodimer of Reelin Protein Linked via Its Central Region,” Journal of Biological Chemistry 286 (2011): 35247–35256, 10.1074/jbc.M111.242719. 21844191 PMC3186359

[39] Y. Yamagishi , I. Shoji , S. Miyagawa , et al., “Natural Product‐Like Macrocyclic N‐Methyl‐Peptide Inhibitors Against a Ubiquitin Ligase Uncovered From a Ribosome‐Expressed De Novo Library,” Chemistry & Biology 18 (2011): 1562–1570, 10.1016/j.chembiol.2011.09.013. 22195558

[40] Y. Goto , A. Ohta , Y. Sako , Y. Yamagishi , H. Murakami , and H. Suga , “Reprogramming the Translation Initiation for the Synthesis of Physiologically Stable Cyclic Peptides,” ACS Chemical Biology 3 (2008): 120–129, 10.1021/cb700233t. 18215017

[41] R. J. Delle Bovi and W. T. Miller , “Expression and Purification of Functional Insulin and Insulin‐Like Growth Factor 1 Holoreceptors From Mammalian Cells,” Analytical Biochemistry 536 (2017): 69–77, 10.1016/j.ab.2017.08.011. 28830678 PMC5701837

[42] D. Rogers and M. Hahn , “Extended‐Connectivity Fingerprints,” Journal of Chemical Information and Modeling 50 (2010): 742–754, 10.1021/ci100050t. 20426451

[43] A. A. Vinogradov , J. S. Chang , H. Onaka , Y. Goto , and H. Suga , “Accurate Models of Substrate Preferences of Post‐Translational Modification Enzymes From a Combination of mRNA Display and Deep Learning,” ACS Central Science 8 (2022): 814–824, 10.1021/acscentsci.2c00223. 35756369 PMC9228559

[44] L. McInnes , J. Healy , and J. Melville , “UMAP: Uniform Manifold Approximation and Projection for Dimension Reduction,” preprint, arXiv, September 18, 2020, 10.48550/ARXIV.1802.03426.

[45] R. J. G. B. Campello , D. Moulavi , and J. Sander , Adv. Knowl. Discov. Data Min., ed. J. Pei , V.S. Tseng , L. Cao , H. Motoda , and G. Xu (Springer Berlin Heidelberg, 2013): 160–172.

[46] F. Pedregosa , G. Varoquaux , A. Gramfort , et al., “Scikit‐learn: Machine Learning in Python,” preprint, arXiv, Jun 5, 2018, 10.48550/ARXIV.1201.0490.

[47] T. Passioura , W. Liu , D. Dunkelmann , T. Higuchi , and H. Suga , “Display Selection of Exotic Macrocyclic Peptides Expressed Under a Radically Reprogrammed 23 Amino Acid Genetic Code,” Journal of the American Chemical Society 140 (2018): 11551–11555, 10.1021/jacs.8b03367. 30157372

[48] T. Kawakami , T. Ishizawa , and H. Murakami , “Extensive Reprogramming of the Genetic Code for Genetically Encoded Synthesis of Highly N‐Alkylated Polycyclic Peptidomimetics,” Journal of the American Chemical Society 135 (2013): 12297–12304, 10.1021/ja405044k. 23899321

[49] T. Kawakami , H. Murakami , and H. Suga , “Ribosomal Synthesis of Polypeptoids and Peptoid−Peptide Hybrids,” Journal of the American Chemical Society 130 (2008): 16861–16863. 19053417 10.1021/ja806998v

[50] T. Fujino , Y. Goto , H. Suga , and H. Murakami , “Reevaluation of the D‐Amino Acid Compatibility With the Elongation Event in Translation,” Journal of the American Chemical Society 135 (2013): 1830–1837. 23301668 10.1021/ja309570x

[51] T. Kawakami , H. Murakami , and H. Suga , “Messenger RNA‐Programmed Incorporation of Multiple N‐Methyl‐Amino Acids Into Linear and Cyclic Peptides,” Chemistry & Biology 15 (2008): 32–42, 10.1016/j.chembiol.2007.12.008. 18215771

[52] T. Katoh , Y. Iwane , and H. Suga , “Logical Engineering of D‐Arm and T‐Stem of tRNA That Enhances D‐Amino Acid Incorporation,” Nucleic Acids Research 45 (2017): 12601–12610, 10.1093/nar/gkx1129. 29155943 PMC5728406

[53] H. Murakami , A. Ohta , H. Ashigai , and H. Suga , “A Highly Flexible tRNA Acylation Method for Non‐Natural Polypeptide Synthesis,” Nature Methods 3 (2006): 357–359, 10.1038/nmeth877. 16628205

[54] Y. Sako , Y. Goto , H. Murakami , and H. Suga , “Ribosomal Synthesis of Peptidase‐Resistant Peptides Closed by a Nonreducible Inter‐Side‐Chain Bond,” ACS Chemical Biology 3 (2008): 241–249, 10.1021/cb800010p. 18338852

[55] R. C. Pillutla , K. Hsiao , J. R. Beasley , et al., “Peptides Identify the Critical Hotspots Involved in the Biological Activation of the Insulin Receptor,” Journal of Biological Chemistry 277 (2002): 22590–22594, 10.1074/jbc.M202119200. 11964401

[56] J. G. Menting , J. Whittaker , M. B. Margetts , et al., “How Insulin Engages Its Primary Binding Site on the Insulin Receptor,” Nature 493 (2013): 241–245, 10.1038/nature11781. 23302862 PMC3793637

[57] F. Weis , J. G. Menting , M. B. Margetts , et al., “The Signalling Conformation of the Insulin Receptor Ectodomain,” Nature Communications 9 (2018): 4420, 10.1038/s41467-018-06826-6. PMC6200814 30356040

