# Supplementary material for: Direct Selection of Functional De Novo Macrocycles for Activation of On‐Cellulo Insulin Receptor
Source: Angew Chem Int Ed Engl. 2026 Mar 12;65(17):e26008. doi: 10.1002/anie.202526008 (PMC13098473; doi:10.1002/anie.202526008)
Supplement: Supplementary file 1 — Supporting File 1: anie71738‐sup‐0001‐SuppMat.docx. [file ANIE-65-e26008-s001.docx]

**Supporting Information for**

**Direct Selection of Functional *De Novo* Macrocycles for Activation of *On-Cellulo* Insulin Receptor**

Yun-Hsuan Kuo^1^, Emiko Mihara^2^, Junichi Takagi^2,^, Hiroaki Suga^1,*^

^1^ Department of Chemistry, Graduate School of Science, The University of Tokyo, Bunkyo-ku, Tokyo, Japan

^2^ Laboratory of Protein Synthesis and Expression, Institute for Protein Research, Osaka University, Suita, Japan

*Correspondence to hsuga@chem.s.u-tokyo.ac.jp

Table of Contents

[Material and Methods 4](#_Toc217081293)

[Materials. 4](#_Toc217081294)

[Protein Expression and Purification of Insulin Receptor. 4](#_Toc217081295)

[Synthesis of Activated Amino Acid Substrates. 5](#_Toc217081296)

[Preparation of Flexizymes and tRNAs. 5](#_Toc217081297)

[Preparation of Aminoacyl-tRNAs. 6](#_Toc217081298)

[mRNA Library Construction. 6](#_Toc217081299)

[Puromycin Ligation of mRNA Library. 7](#_Toc217081300)

[In Vitro Translation and Reverse Transcription. 8](#_Toc217081301)

[Fidelity of Non-Proteinogenic Amino Acid Incorporation. 9](#_Toc217081302)

[RaPID Selection against the Ectodomain of Insulin-Receptors. 9](#_Toc217081303)

[RaPID-Excells Selection against Endogenous Insulin Receptors. 10](#_Toc217081304)

[Site-Saturation Mutagenesis Scanning against Endogenous Insulin Receptors. 11](#_Toc217081305)

[Next Generation Sequencing (NGS). 12](#_Toc217081306)

[NGS Analysis. 13](#_Toc217081307)

[Enrichment Score Calculation for Site-Saturation Mutagenesis. 14](#_Toc217081308)

[Chemical Synthesis of Thioether-Closed Peptides. 14](#_Toc217081309)

[Agonist Activity of Insulin Receptor by Immunoblotting. 15](#_Toc217081310)

[Affinity Measurement by Surface Plasmon Resonance. 16](#_Toc217081311)

[Supporting Figures 17](#_Toc217081312)

[Figure S1. 17](#_Toc217081313)

[Figure S2. 18](#_Toc217081314)

[Figure S3. 19](#_Toc217081315)

[Figure S4. 20](#_Toc217081316)

[Figure S5. 21](#_Toc217081317)

[Figure S6. 22](#_Toc217081318)

[Figure S7. 23](#_Toc217081319)

[Figure S8. 24](#_Toc217081320)

[Figure S9. 25](#_Toc217081321)

[Figure S10. 26](#_Toc217081322)

[Figure S11. 27](#_Toc217081323)

[Figure S12. 28](#_Toc217081324)

[Figure S13. 29](#_Toc217081325)

[Figure S14. 30](#_Toc217081326)

[Figure S15. 31](#_Toc217081327)

[Figure S16. 32](#_Toc217081328)

[Figure S17. 33](#_Toc217081329)

[Figure S18. 34](#_Toc217081330)

[Figure S19. 35](#_Toc217081331)

[Figure S20. 36](#_Toc217081332)

[Figure S21. 37](#_Toc217081333)

[Figure S23. 39](#_Toc217081334)

[Figure S24. 40](#_Toc217081335)

[Figure S25. 41](#_Toc217081336)

[Figure S26. 42](#_Toc217081337)

[Supporting Tables 43](#_Toc217081338)

[Table S1. Primers for construction of flexizymes and tRNAs. 43](#_Toc217081339)

[Table S2. Primers for construction of NNK and focused mRNA libraries. 43](#_Toc217081340)

[Table S3. Primers for mRNA library construction in site-saturation mutagenesis. 44](#_Toc217081341)

[Table S4. Primers for RaPID screening and NGS. 48](#_Toc217081342)

[Table S5. Aminoacylation of tRNAs with non-proteinogenic amino acids. 50](#_Toc217081343)

# Material and Methods

**Materials.** Boc-protected and Fmoc-protected amino acids were purchased from Watanabe Chemical Industries, Ltd. Other reagents were purchased from Nacalai Tesque, Inc. unless otherwise specified.

**Protein Expression and Purification of Insulin Receptor.** To produce soluble hIR ectodomain protein for the RaPID selection, the entire ectodomain region (residues 1-956) was PCR-amplified from the cDNA clone (#H04D054L22, Danaform) and appended before the monomeric version of human IgG1 Fc (mFc), followed by a biotin acceptor sequence (BAS) and a 10x His tag in the pcDNA3.1 vector (Thermo Fisher). The rationale for employing mFc, a stable monomeric human IgG1 Fc carrying mutations T366R, L368H, P395K, K409T, and M428L^42^, is to avoid distortion of soluble IR ectodomain dimer conformation that may occur when fusing with strongly hoodimerizing Fc. Protein production was conducted using the Expi293F Expression system (Thermo Fisher Scientific). The Expi293F cells were co-transfected with hIRec-mFc-BAS-His and BirA-ΔKDEL (an ER-resident mutant of the biotin ligase BirA)^43^, followed by addition of 100 µM D-biotin (Nacalai Tesque) to the culture medium one day post-transfection. The supernatant was harvested four days post-transfection and mixed with Ni-NTA agarose (QIAGEN) and rotated for 2 hours at room temperature. The agarose was transferred to an Econo-Column (Bio-Rad) and washed three times with 10 column volumes of Tris-buffered saline (TBS, 20 mM Tris, 150 mM NaCl, pH 7.5). Proteins were eluted with TBS containing 250 mM imidazole. The fractions containing hIR-mFc-BAS-His were combined and dialyzed against phosphate-buffered saline (PBS, 10 mM Na-phosphate, 140 mM NaCl, 2.7 mM KCl, pH 7.4) at 4 °C. Purified protein was concentrated using an Amicon Ultra-4 Centrifugal Filters Ultracel®-30K and stored at -80 °C until use.

**Synthesis of Activated Amino Acid Substrates.** Activated amino acid substrates were synthesized according to previously reported methods^44–48^. In brief, cyanomethyl-ester (CME) and dinitrobenzyl-ester (DEB) substrates were synthesized from commercial boc-protected amino acids. The reaction mixture was stirred at room temperature in the dark. After reaction for 16 h, the mixture was diluted by adding ethyl acetate and subsequently washed with 1M HCl_(aq)_, water, saturated NaHCO_3_ and brine. The organic layer was dried over anhydrous sodium sulfate and concentrated under vacuum. The residue was dissolved in a minimal amount of ethyl acetate and subjected to purification by flash column chromatography using n-hexane/ethyl acetate solvent system. To a solution of DBE derivative in DCM (2 mL), 4 M HCl in dioxane was added. After stirring at room temperature for 1 h, the final products were precipitated by adding cold diethyl ether. The solids were washed with cold diethyl ether for three times and dried under vacuum.

**Preparation of Flexizymes and tRNAs.** Flexizymes (eFx and dFx)^26^ and tRNAs (tRNA^fMet^_CAU_ and tRNA^Pro1E2^_CAU_)^49^ were transcribed *in vitro* by T7 RNA polymerase based on designed DNA templates as previously described. For construction of DNA templates, extension and PCR solutions were prepared using corresponding primer sets (2 µM each for extension and 0.5 µM each for PCR) (Table S1) in a reaction buffer containing 10 mM Tris (pH 9.0), 50 mM KCl, 2.5 mM MgCl_2_, 0.1 vol% Triton X-100, 0.25 mM dNTP mix and 60 nM *Taq* DNA polymerase. Extension reaction was initiated at 95 ℃ for 1 min, and followed by 5 cycles of annealing at 50 ℃ and extension at 72 ℃ for 1 min each. Subsequently, 10 µL of the extension products was added to 1 mL of PCR solution, and amplified by 12 cycles of PCR reactions (denaturation at 95 ℃, annealing at 50 ℃ and extension at 72 ℃ for 40 sec each). The PCR amplification was checked by 3% agarose gel, and the resulting products were purified via phenol-chloroform extraction and ethanol precipitation. Transcription of flexizymes was carried out at 37 ℃ overnight in 1 mL of a reaction mixture containing 40 mM Tris-HCl (pH 8.0), 1 mM spermidine, 0.01 vol% Triton X-100, 20 mM MgCl_2_, 10 mM DTT, 5 mM NTP mix, 0.04 U/µL RNasin RNase inhibitor (Promega) and 0.12 µM T7 RNA polymerase. For transcription of tRNAs, additional 5 mM guanosine monophosphate (GMP) was added to the above mixture and 3.75 mM NTP mix was used instead. After the transcription, the RNA transcripts were treated with RQ1 DNase (Promega) for 1 hr at 37 ℃, and purified by electrophoresis at 230 V for 1 hr through 8% (tRNAs) or 12% (flexizymes) polyacrylamide gels containing 6 M Urea. The desired band was cut from the gels, grinded, and followed by extraction three times with each 3 mL of 0.3 M NaCl for 1 hr. The purified products were collected by ethanol precipitation.

**Preparation of Aminoacyl-tRNAs.** Aminoacylation of tRNA under the catalysis of flexizymes was performed according to the method described previously^50^. In this study, *N*-chloroacetyl-l-Tyr-CME (ClAc-l-Tyr-CME) was charged onto tRNA^fMet^_CAU_ for translation initiation and the other activated amino acids were charged to tRNA^Pro1E2^_CAU_ for translation elongation. Aminoacylation of tRNA was carried out at 4 ℃ for specified time under the catalysis of flexizymes in a reaction buffer containing 50 mM HEPES-KOH (pH 7.5), 0.6 M MgCl_2_, 20 % (vol/vol) DMSO, 25 µM Fx, 25 µM tRNA and 5 mM pre-activated amino acid substrates (Table S5). The resulting aminoacyl-tRNAs were precipitated by 70% (vol/vol) ethanol containing 75 mM sodium acetate (pH 5.2). The solution was centrifuged for 15 min at 13,000 rpm and the supernatant was discarded. The pellets were washed twice with 70% (vol/vol) ethanol containing 100 mM sodium acetate (pH 5.2) and once with 70% (vol/vol) ethanol.

**mRNA Library Construction.** DNA libraries were constructed through PCR amplification utilizing oligonucleotide primers outlined in Table S2 (NNK and focused libraries) and Table S3 (deep scanning library). The initial extension was performed with 3 cycles of denaturation at 98 ℃ for 10 sec, annealing at 55 ℃ for 5 sec and extension at 68 ℃ for 5 sec in a 1 mL of KOD One^TM^ PCR master mix containing 0.5 µM forward and reverse overlapping primer pairs. Subsequently, the entire extension production was combined with a 1 mL of KOD One^TM^ PCR master mix containing 0.5 µM primer pairs, and subjected to four cycles of denaturation at 98 ℃ for 10 sec, annealing at 61 ℃ for 5 sec and extension at 68 ℃ for 5 sec were conducted for PCR amplification. Following amplification, the DNA products were purified by phenol-chloroform-isoamyl (PCI) and chloroform-isoamyl (CI) extraction, and then evaluated by 4200 TapeStation (Agilent). The extracted DNA was precipitated with 70% (v/v) ethanol in water, followed by dissolution in water. This solution was then added to a transcription reaction mix containing 40 mM Tris (pH 8.0), 1 mM spermidine, 3.75 mM each NTP, 20 mM MgCl_2_, 10 mM DTT, 0.01% (v/v) Triton X-100, 240 nM T7 polymerase, and 0.04 U/µL RNasin RNase inhibitor (Promega). T7 transcription was allowed to proceeded at 37 ℃ for 14 hr. DNA within the reaction products was digested by the addition of RQ1 RNase-free DNase (Promega), followed by further incubation at 37 ℃ for 60 min. The reaction was quenched by the addition of EDTA and NaCl to final concentrations of 67 mM and 270 mM, respectively. The transcripts were precipitated with 45% (v/v) isopropanol in water, washed by 70% (v/v) ethanol in water, dissolved in water, and purified through electrophoresis using a 6% polyacrylamide gel containing 6 M urea. The resulting RNA products were extracted from the gel with 300 mM NaCl_(aq)_, precipitated by 70% (v/v) ethanol in water, dissolved in water and stored at -80 ℃.

**Puromycin Ligation of mRNA Library.** Individual mRNA libraries were attached to a puromycin-linker via Y-ligation using T4 RNA ligase. The ligation reaction was carried out at 25 ℃ for 30 min in a buffer comprising 40 mM Tris (pH 7.8), 10 mM MgCl_2_, 10 mM DTT, 0.5 mM ATP, 20% (vol/vol) DMSO, 1 µM mRNA library, 1.5 µM puromycin-linker and 0.3 µM T4 RNA ligase. The resulting mRNA-puromycin conjugates were purified through PCI/CI extraction, followed by washing and precipitation with 70% (v/v) ethanol in water. Subsequently, the conjugates were dissolved in water, and the concentration of the ligation products was adjusted to 6 µM before storage at –80 ℃.

***In Vitro* Translation and Reverse Transcription.** Ribosome synthesis of thioether-closed macrocyclic peptides from mRNA was carried out via a flexible *in vitro* translation (FIT) system as previous described^51^. In this study, methionine was removed from FIT system and substituted with ClAc-l-Tyr-tRNA^fMet^_CAU_ to generate peptide libraries initiated with ClAc-l-Tyr. The translation reaction was performed at 37 ℃ for 30 min in a reaction buffer containing 50 mM HEPES-KOH (pH 7.6), 100 mM KOAc, 12 mM Mg(OAc)_2_, 20 mM creatine phosphate, 2 mM ATP, 2 mM GTP, 2 mM CTP, 2 mM UTP, 2 mM spermidine, 1 mM DTT, 1.5 mg/mL *E. coli* total tRNA (Roche), 1.2 µM *E. coli* ribososme, 0.6 µM methionyl-tRNA formyltransferase, 2.7 µM IF1, 0.4 µM IF2, 1.5 µM IF3, 20 µM EF-Tu, 20 µM EF-Ts, 0.1 µM EF-G, 0.25 µM RF2, 0.17 µM RF3, 0.5 µM RRF, 4 µg/mL creatine kinase, 3 µg/mL myokinase, 0.1 µM pyrophosphatase, 0.1 µM nucleotide diphosphate kinase, 0.1 µM T7 RNA polymerase, 0.73 µM AlaRS, 0.02 µM CysRS, 0.13 µM AspRS, 0.23 µM GluRS, 0.15 µM PheRS, 0.09 µM GlyRS, 0.02 µM HisRS, 0.4 µM IleRS, 0.11 µM LysRS, 0.04 µM LeuRS, 0.03 µM MetRS, 0.38 µM AsnRS, 0.16 µM ProRS, 0.06 µM GlnRS, 0.03 µM ArgRS, 0.04 µM SerRS, 0.09 µM ThrRs, 0.04 µM ValRS, 0.03 µM TrpRS, 0.02 µM TyrRS, 500 µM each proteinogenic amino acid except methionine, 50 µM ClAc-l-Tyr-tRNA^fMet^_CAU_, and 1 µM puromycin-conjugated mRNA library. Following a further incubation at 25 ℃ for 10 min, the translation product was subjected to incubation at 37 ℃ for 30 min in presence of 16.67 mM EDTA. This step aimed to dissociate ribosomal subunits and facilitate cyclization. The resulting mRNA-linked macrocyclic peptides were subsequently reverse transcribed at 42 ℃ for 1 h using the corresponding reverse primers (CGS3an13.R39 for NNK and focused libraries, and SGG2an13.R36 for site-saturation mutagenesis) in presence of M-MLV reverse transcriptase (RNase H-) (Promega).

**Fidelity of Non-Proteinogenic Amino Acid Incorporation.** tRNA^fMet^_CAU_ aminoacylated with N-chloroacetyl-tyrosine and tRNA^Pro1E2^_CAU_ aminoacylated with one of the non-proteinogenic amino acids were employed under the catalysis of flexizymes, as indicated in Table S5. Subsequently, the FIT translation system was supplemented with a DNA template name YK2_ATG_DNA. This template was designed with an ATG codon for tRNA^fMet^_CAU_ initiation, an ATG codon for tRNA^Pro1E2^_CAU_ elongation, and a TAA stop codon for stalling translation. The translation process was conducted at 37 ℃ for 30 min. The translated peptide was isolated using a C18 C-tip (WAKO), eluted with 50% saturated ɑ-cyano-4-hydrocinnamic acid in 80% acetonitrile/water, 0.5% acetic acid and spotted onto a MALDI plate. The m/z value of peptides was determined using MALDI-TOF in positive reflector mode (Bruker).

YK2_ATG_DNA =

GGCGTAATACGACTCACTATAGGGTTAACTTTAAGAAGGAGAAAAACATGTACAAGAAATACAAAATGGACTACAAGGACGACGACGACAAGTAAGCTTCG

**RaPID Selection against the Ectodomain of Insulin-Receptors.** The *in vitro* selection of thioether-macrocyclic peptides was conducted against Fc-fused human IR ectodomain (hIRec-mFc) using mRNA libraries encoding random peptide sequences ranging from 11-mer to 15-mer. The hIRec-mFc was expressed and purified according to the methods described above. The mRNA libraries were conjugated with puromycin, translated into macrocyclic peptides, and subjected to reverse transcriptions of the mRNA to cDNA, following the methods mentioned above. In the first round, translation was carried out on a scale of 150 µL to encompass the diversity of peptide library. Subsequent rounds, starting from round 2, involved a scaled-down translation volume of 5 µL. The translation products were mixed with an equal volume of a blocking buffer containing 100 mM Tris (pH 7.6), 300 mM NaCl, 0.1% (v/v) Tween-20 and 2 mg/mL acetylated BSA. The mRNA-peptide mixture underwent three rounds of negative selections followed by a positive selection. For the negative selections, mixtures of Dynabeads Protein G (Thermo Fisher Scientific) and Fc-immobilized Dynabeads Protein G were utilized (R&D Systems). For the positive selection, the expressed hIRec-mFc dimers were immobilized onto Dynabeads Protein G, and the bead amount was measured to achieve a concentration of 300 nM of proteins in the solution. In each selection, the solution and beads were mixed at 4 ℃ for 30 min, and then the beads were washed three times with a TBS-T buffer containing 50 mM Tris (pH 7.4), 150 mM NaCl, 0.05% (v/v) Tween-20. The pulled-down peptide-cDNA conjugates on the negative and positive beads were eluted at 95 ℃ for 5 min in a KOD One PCR mix containing forward and reverse primers, T7g10M.F46 and CGS3an13.R39 (Table S4). The amount of cDNA was evaluated by qPCR using SYBR Green I and LightCycler 96 (Roche). The selected products were amplified by PCR with a cycle number determined based on the qPCR results. The amplified DNA was purified, precipitate by 70% (v/v) ethanol in water, and dissolved in water. The recovered DNA was identified by next generation sequencing using Miseq sequencing system (Illumina). For the subsequent round of selection, the DNA solution was added to a transcription mix containing 40 mM Tris (pH 8.0), 1 mM spermidine, 3.75 mM each NTP, 20 mM MgCl_2_, 10 mM DTT, 0.01% (v/v) Triton X-100, 240 nM T7 polymerase, and 0.04 U/µL RNasin RNase inhibitor (Promega). T7 transcription was allowed to proceeded at 37 ℃ overnight. The resulting mRNA was purified by 45% isopropanol in water, washed and precipitated by 70% ethanol in water, and dissolved in water for the next round of selection.

**RaPID-Excells Selection against Endogenous Insulin Receptors.** HEK293H cells (Thermo Fisher Scientific) were plated in a 12-well plate at a seeding density of 1 × 10^5^ cells per well and cultured at 37 ℃ and 5% CO_2_ in DMEM medium supplemented with 10% Fetal Bovine Serum (FBS) for 24 hr. Following this, the cells were starved in DMEM without FBS at 37 ℃ and 5% CO_2_ for 4 hr. The mRNA-peptide fusions for selections were prepared using the methods described above. The translation products were desalted and exchanged to a TBS buffer containing 50 mM Tris (pH 7.6) and 150 mM NaCl using Sephadex G25 column (Sigma-Aldrich) before cell treatment. After serum-starvation, the cells were stimulated with the desalted solutions at 37 ℃ for 10 min. Subsequently, the stimulated cells were washed three times with warm FBS free DMEM medium and once with cold PBS buffer. Following the washes, the cells were incubated with 150 µL of a cold lysis buffer containing 50 mM HEPES (pH 7.4), 150 mM NaCl, 20 mM sodium pyrophosphate, 5 mM sodium fluoride, 2 mM sodium orthovanadate, 1 mM EDTA, 2 mM phenylmethylsulfonyl fluoride (PMSF), 1% (v/v) Triton X-100, 10% glycerol (v/v), cOmplete Protease Inhibitor Cocktail (Roche) and PhosSTOP (Roche) on ice for 1 hr. The cell lysates were scraped and collected from the plate, then centrifuged at 11,000 g at 4 ℃ for 15 min. The 100 µL of supernatant was collected and mixed with 5 µL of 20 mg/mL acetylated BSA. The solutions were incubated with 10 µL of Dynabeads M280 Sheep Anti-Rabbit IgG (Thermo Fisher Scientific) at 4 ℃ for 1 hr as negative selection. Subsequently, the supernatant was mixed with rabbit anti-IR-β mAb (Cell Signaling #3025) and incubated at 4 ℃ overnight. Following this incubation, co-immunoprecipitation was carried out by mixing the solutions with 10 µL of Dynabeads M280 Sheep Anti-Rabbit IgG at 4 ℃ for 2 hr, considered as a positive selection. After the selections, the beads were washed three times with a TBS-X buffer containing 50 mM Tris (pH 7.6), 150 mM NaCl and 1% (v/v) Triton X-100. The pulled-down peptide-cDNAs on the beads were eluted at 95 ℃ for 5 min in a KOD One PCR mix containing the primer pairs, T7g10M.F46 and CGS3an13.R39. The resulting cDNAs were analyzed and amplified using the methods described in *in vitro* selection.

**Site-Saturation Mutagenesis Scanning against Endogenous Insulin Receptors.** The incorporation of non-proteinogenic amino acids (npAAs) for site-saturation mutagenesis was previously described^31^. In this study, we extended this scanning technique to target the endogenous insulin receptor in live cells using RaPID-Excells. To construct DNA templates for site-saturation mutagenesis, a single NNK or ATG codon was introduced at the mutation site of interest (Table S3). The NNK codon scanned natural amino acids, while the ATG codon explored non-proteinogenic amino acids. A total of 19 natural amino acids and 47 npAAs were introduced to scan the HL4 peptide sequence. To identify the incorporated amino acid, a unique barcode was added to the tail of each scanning DNA library (highlighted in red in Table S3), resulting in 48 barcodes: one for the NNK library and 47 for npAAs. Activated npAAs were loaded onto tRNA^Pro1E2^_CAU_ through the catalysis of flexizymes, as specified in Table S5. Each npAA was introduced to its corresponding barcode-encoded mRNA library using the FIT system, along with an additional 50 µM npAA-tRNA^Pro1E2^_CAU_ for elongation at the second ATG codon. The translated mutants were mixed with an equal volume of blocking buffer containing 100 mM Tris (pH 7.6), 300 mM NaCl, 0.1% (v/v) Tween-20 and 2 mg/mL acetylated BSA. This mixture was incubated with anti-HA magnetic beads (Thermo Fisher) at 4 ℃ for 1 hr. Beads were washed three times with ice-cold TBS-T buffer to remove unligated mRNAs and incompletely-translated peptides. The C-terminal HA-tag ensured that only fully translated peptide products were retained, and they were eluted at 37 ℃ for 30 min using a TBS-T buffer containing 2 mg/mL HA peptide and 1 mg/mL acetylated BSA. The HA-purified solution was then incubated with Bio-Bead SM-2 Resin (Bio-Rad) to remove detergents. Subsequently, the products were desalted and exchanged to a TBS buffer using Sephadex G25 column (Sigma-Aldrich) before cell treatment. The selections of the mutant libraries targeting endogenous IR were carried out following the cell-based selection mentioned above.

**Next Generation Sequencing (NGS).** The recovered cDNA from each selection underwent a two-step tailed PCR to incorporate initiation sequences (Rd1 SP and Rd2 SP) and indexed sequences, following the Nextera XT v2 Set primers (Illumina). In the first-step PCR, denaturation at 98 ℃ for 10 sec, annealing at 63 ℃ for 5 sec, and extension at 68 ℃ for 5 sec were performed with 6 cycles. This step utilized a KOD OneTM PCR master mix and 0.5 µM primer pairs, Rd1T7g10M.F70 and an13Rd2.R49 (Table S4). Subsequently, 1 µL of the resulting products was added to 100 µL of a KOD OneTM PCR master mix containing 0.5 µM indexed primer pairs. The second-step PCR was conducted with 8 cycles of denaturation at 98 ℃ for 10 sec, annealing at 52 ℃ for 5 sec, and extension at 68 ℃ for 5 sec. The PCR products were assessed using a 4200 TapeStation (Agilent) and purified with a NucleoSpin kit (TaKaRa). DNA concentration was measured with a Qubit dsDNA BR assay kit and a Qubit 3.0 fluorometer (Thermo Fisher). The DNA was appropriately diluted to 4 nM with a buffer containing 10 mM Tris-HCl (pH 8.5) and 0.1% Tween-20 (v/v), then denatured in 0.1 M NaOH at room temperature for 5 min. The denatured library, mixed with 50% (mol/mol) PhiX Control v3 (Illumina), was diluted in Hyb buffer to a final concentration of 10 pM and subsequently sequenced on a Miseq instrument (Illumina) using single-read 1x251 cycle mode with v2 chip.

**NGS Analysis.** The original Python code is available at <https://github.com/avngrdv/clibas>. In essence, the code facilitated the parsing of .fastq files and transforming DNA sequences into peptide sequences. The resulting peptides underwent filtering to exclude sequences with incorrect length, open reading frames lacking stop codons, ORFs containing ambiguous symbol, and Q scores below 20. For visualizing sequences convergence, the top 1000 peptides were transformed into matrices of extended-connectivity fingerprints (ECFP)^52^ based on the chemical structures of constituent amino acids, utilizing the rdkit package ([http://www.rdkit.org](http://www.rdkit.org/)) with a max_radius parameter set to 4. Accurate Models of Substrate Preferences of Post-Translational Modification Enzymes from a Combination of mRNA Display and Deep Learning^53^. Subsequently, two-dimensional embeddings of the peptides were generated using UMAP (Uniform Manifold Approximation and Projection) with optimal parameters (n_neighbors and min_dist) determined by the sequence convergence of the peptide datasets (<https://github.com/lmcinnes/umap>)^54^. The resulting data, projected onto the UMAP1 and UMAP2 axes, were visualized to illustrate the convergence of analyzed peptide sequences. Furthermore, HDBSCAN (hierarchical density-based spatial clustering of applications with noise) was applied for clustering, optimizing clustering hyperparameters (min_cluster_size and min_samples) based on the overall sequence conservation and the size of the resulting clusters (<https://github.com/scikit-learn-contrib/hdbscan>)^55,56^.

**Enrichment Score Calculation for Site-Saturation Mutagenesis.** The enrichment score (*E*) was calculated using the Equations [1-3], with a value of *E* = 0 indicates identical enrichment to the wild-type peptide. The fraction of reads for each peptide sequence, denoted as *f_i_*, was determined by the formula:

$f_{i}=\frac{N_{i}}{\sum N_{i}}$ , [1]

where *N_i_* is the number of reads of a peptide sequence and $\sum N_{i}$ represents the total number of reads across all peptides. Raw enrichment score (*e_i_*) for each mutant *i* was derived by

$e_{i}=\frac{f_{i (positive)}}{f_{i (negative)}}$, [2]

where the values of *f_i_* _(positive)_ and *f_i_* _(negative)_ are the fractions of the mutant *i* sequenced from the positive and the negative selections. The *E_i_* for each mutant was then calculated by normalizing the *e_i_* of the mutant to that of the wild-type peptide:

$E_{i}=\frac{e_{i}}{e_{i (wild-type)}}$ . [3]

Here, *e_i_* _(wild-type)_ represents the raw enrichment score of wild-type peptide sequence. The resulting values of Log_2_(*E_i_*) for each mutant were calculated for visualization in a heatmap plot. Peptides with Log_2_(*E*) > 0 indicate increased enrichment relative to the wild-type sequence.

**Chemical Synthesis of Thioether-Closed Peptides.** Macrocyclic peptides were synthesized by solid phase peptide synthesis (SPPS) using Fmoc-protected amino acids. After the reaction, the N-terminal of the peptides was couple with N-(choloroacetoxy)succinimide to yield N-chloroacetyl peptides. The peptides were subsequently cleaved using a TFA cocktail containing TFA (trifluoroacetic acid), water, TIS (triisopropylsilane), and DODT (3,6-dioxa-1,8,-octanedithiol) in a ratio of 92.5:2.5:2.5:2.5 for 2 h. The cleavage mixture was followed by cold ether precipitation to isolate the peptides. The N-chloroacetyl peptides were dissolved in a solution containing 80% DMSO and water. The cyclization process was initiated by adding triethylamine to the solution, followed by incubation at room temperature for 1 h. The cyclic peptides were purified by HPLC using column with a gradient of 0-60% B.

**Agonist Activity of Insulin Receptor by Immunoblotting.** HEK293H cells were initially seeded in a 6-well plate at a density of 2 × 10^5^ cells per well and maintained at 37 ℃ with 5% CO_2_ in DMEM medium supplemented with 10% FBS for 24 h. Subsequently, the cells were serum-starved by culturing them in DMEM without FBS for an additional 24 hr. Following the serum-starvation, the cells were treated with peptides or insulins at the indicated concentrations at 37 ℃ for 5 min. After the treatment, the cells were gently washed with cold phosphate-buffered saline (PBS) and subsequently incubated with a lysis buffer containing 50 mM HEPES (pH 7.4), 150 mM NaCl, 1% (v/v) Triton X-100, 10% (v/v) glycerol, 1 mM EDTA, 100 mM sodium fluoride, 20 mM sodium pyrophosphate, 2 mM sodium orthovanadate, 2 mM phenylmethylsulfonyl fluoride (PMSF), as well as cOmplete Protease Inhibitor Cocktail (Roche) and PhosSTOP (Roche) for 1 hr. The cell lysates were centrifuged at 20,817 g at 4 ℃ for 15 min. The total protein concentration of the lysate was measured by Micro BCA Protein Assay Kit (Thermo Scientific). Cell lysates containing 50 µg of total protein were then analyzed by SDS-PAGE on 8% acrylamide gels and transferred to polyvinylidene fluoride (PVDF) membrane (Thermo Scientific) using an iBlot 2 Gel Transfer Device (Thermo Scientific). The membrane was blocked by incubating it with a blocking buffer containing 50 mM Tris-HCl (pH 7.6), 150 mM NaCl, 0.05% Tween-20 (v/v) and 5% (w/v) bovine serum albumin for 30 min at room temperature. The appropriate primary antibodies, either anti-IR-β (Cell Signaling #3025; labeled as IRβ) or anti-IR-pY1150/1151 (Cell Signaling #3024; labeled as pY-IRβ), was added and incubated at 4 ℃ overnight on an orbital shaker. The next day, the membrane was washed with TBS-T containing 50 mM Tris-HCl (pH 7.6), 150 mM NaCl, 0.05% Tween-20 (v/v) three times for 5 min each. Secondary antibodies in blocking buffer were added and incubated for 1 hr at room temperature, with anti-rabbit-immunoglobulin G (IgG) (H+L chain) pAb-HRP (MBL) used as the secondary antibody. After incubation, the membrane was washed with TBS-T as described above and incubated with ECL Prime Western Blotting Detection Reagent (Fisher Scientific) for 5 min. Chemiluminescence was recorded using the ChemiDoc Touch Imaging System (Bio-Rad). For blotting quantification, the phosphorylation level of pY-IRβ were normalized to total protein (IRβ) and shown as intensities relative to that treated with insulins.

**Affinity Measurement by Surface Plasmon Resonance.** The study investigated the binding affinities between cyclic peptides and IR using a Biacore 8K instrument (cytiva) operated at 25°C. The assay buffer formulation comprised with 50 mM Tris (pH 7.4), 150 mM NaCl, 0.05% (v/v) Tween-20, and 0.1% (v/v) DMSO. hIRec-mFc dimers were immobilized on a Sensor Chip Protein G (cytiva) following standard manufacturer protocols. Single-cycle kinetics were employed for the interaction sensorgrams, with indicated ligand concentrations sequentially flowed over the chip at a rate of 30 μL/min. The association and dissociation phases were set at 30-40 seconds and 60-70 seconds between steps, respectively, and the final dissociation step extended to 200 seconds. Analysis was performed using a 1:1 binding model to determine kinetic parameters and interaction affinities accurately.

# Supporting Figures

**
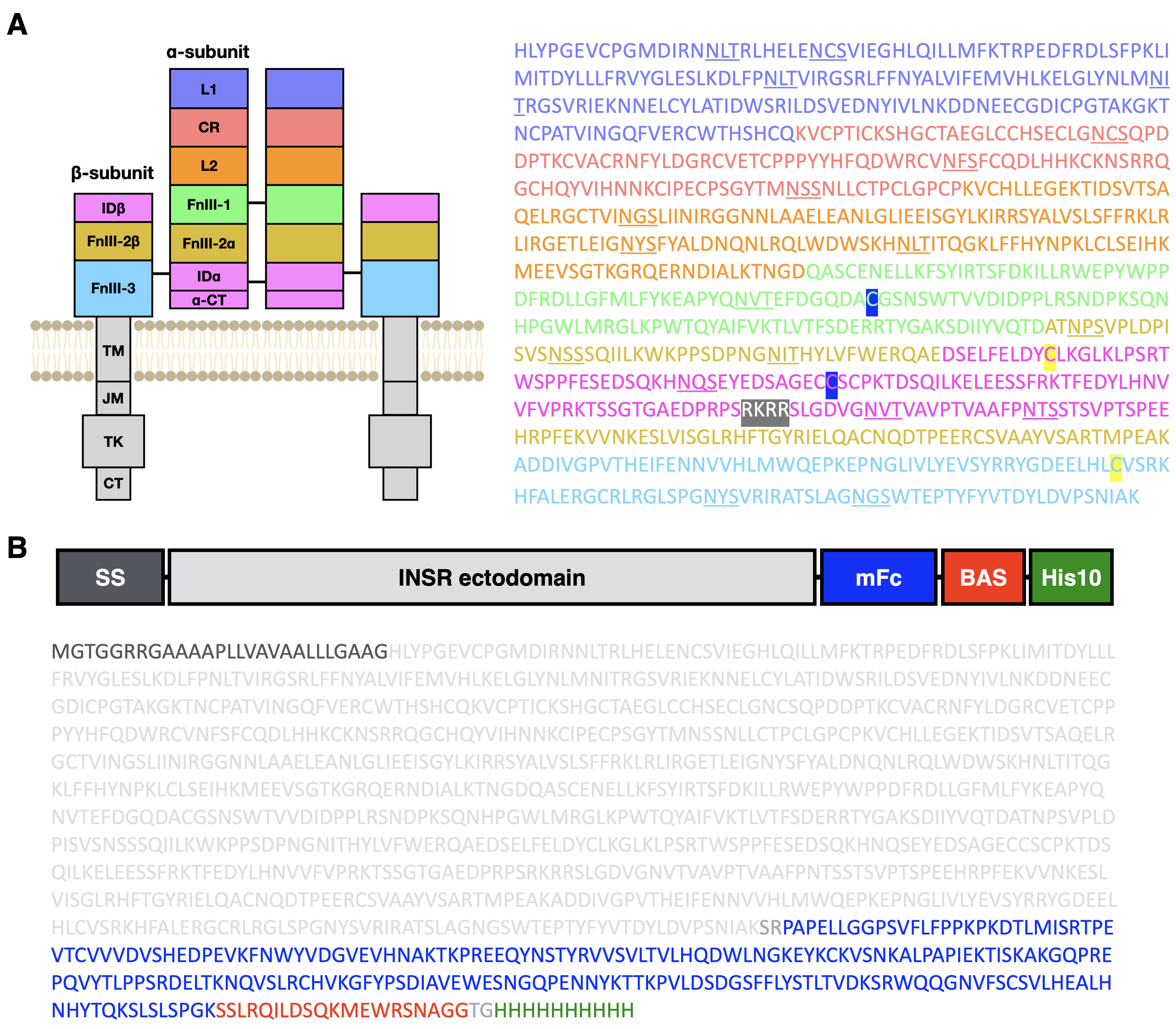
**

**Figure S1.** Protein construction of insulin receptors. (A) Domain architecture of a human insulin receptor (IR) dimer and its ectodomain protein sequence. The IR dimer comprises two ɑ- and two β-subunits linked by disulfide bonds. The ɑ-subunit carries the insulin-binding region composed of a leucine-rich repeat (L1), a cysteine-rich region (CR), a second leucine-rich repeat (L2), two fibronectin type III domains (FnIII-1 and FnIII-2ɑ), an insert domain ɑ (IDɑ), and an ɑ-helical C-terminal domain (ɑ-CT). The β-subunit includes extracellular insert domain β (IDβ), two fibronectin type III domains (FnIII-2β and FnIII-3), a transmembrane helix (TM), juxtamembrane (JM), tyrosine kinase (TK), and C-terminal tail (CT) domains. The corresponding protein sequence for the IR ectodomain is displayed on the right, with colors aligning with the domain architecture. A sequence of RKRR recognized for the cleavage of IR precursor is highlighted in gray. Cysteines used for the formation of inter- and intra-disulfide bonds are indicated in blue and yellow respectively. The Asn-X-Ser/Thr motifs for Asn-glycosylation are denoted by underscores. There are 15 glycosylation positions within the ɑ-chain and 4 positions within the β-subunit. (B) Construction of the recombinant Fc-fused human IR ectodomain (hIRec-mFc). The signal sequence (SS) is positioned at the *N*-terminal of the IR sequence, serving to direct the localization of the expressed IR. The ectodomain of the human IR, composed of both α-subunit and β-subunit components, is seamlessly integrated. Following this, a monomeric Fc (mFc) protein sequence is present. A BAS tag highlighted in red has been incorporated for biotinylation at the central Lys position. Subsequently, ten consecutive histidine residues are introduced.


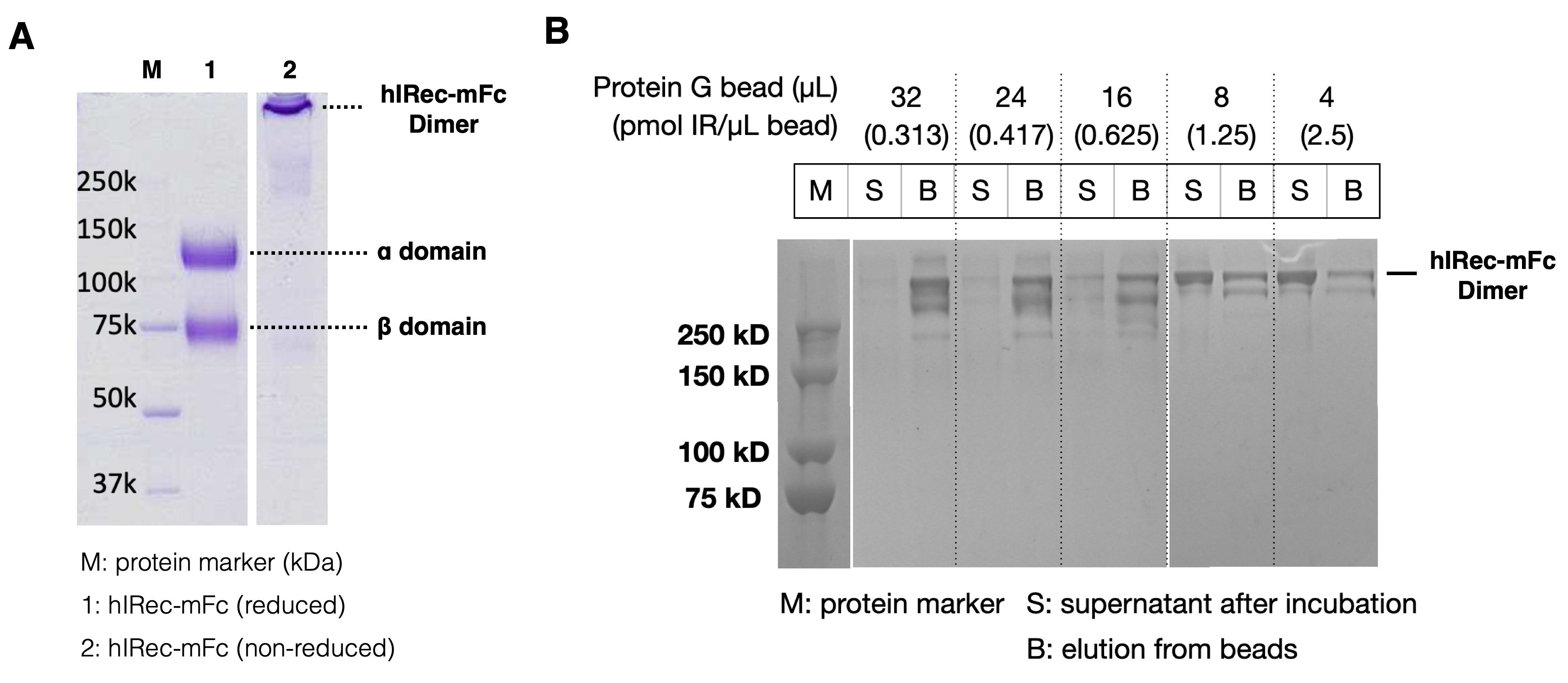


**Figure S2.** Immobilization of target proteins. (A) SDS-page analysis of purified hIRec-mFc proteins. Electrophoresis was conducted using a 10% c-pagel. The inter- and intra-sulfide bonds of a hIRec-mFc dimer were reduced by adding DTT to the loading buffer. The α-domain and the β-domain were visualized under reducing conditions, revealing two distinct bands. In the non-reducing condition, a dimer form of hIRec was observed. (B) Immobilization of hIRec-mFc dimers onto Protein G beads. The immobilization of hIRec-mFc dimers onto Protein G beads was analyzed using a 6% SDS acrylamide gel. A quantity of 10 pmol of hIRec-mFc dimer was incubated with the indicated volume of beads at 4 ℃ for 15 min in a TBS-T buffer containing 50 mM Tris (pH 7.4), 150 mM NaCl, and 0.05% (v/v) Tween-20. The gel analysis illustrates the saturated binding of IR with beads, where 10 pmol of IR was incubated with 16 µL of Protein G beads (0.417 pmol IR/µL bead), a crucial step for subsequent *in vitro* selections.


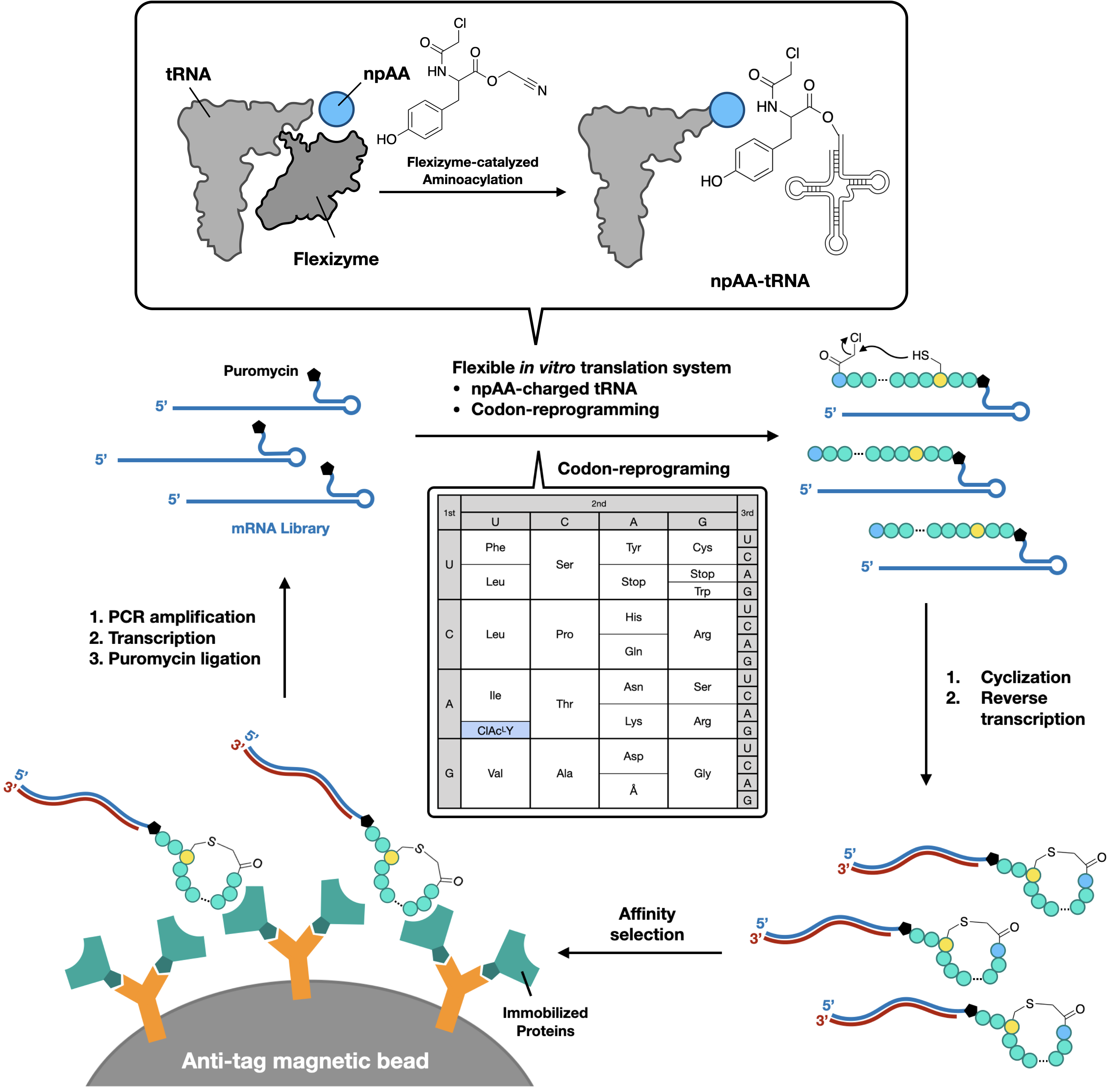


**Figure S3.** RaPID platform with introduction of a non-proteinogenic amino acid (npAA). Flexizyme is a ribozyme that facilitates the aminoacylation of tRNA, allowing it to interact with an activated substrate featuring a cyanomethyl ester group, thereby producing npAA-tRNA. In this example, *N*-chloroacetyl-_L_-tyrosine (ClAc^L^-Y) is charged onto tRNAf^Met^_CAU_ under the catalysis of flexizymes, resulting in the formation of ClAc^L^-Y-tRNA^Met^_CAU_. For RaPID, the selection process begins with a mRNA library, which is conjugated with puromycin linkers for subsequent *in vitro* peptide synthesis. Genetic code reprogramming is harnessed to incorporate the npAA to an assigned codon using a flexible *in vitro* translation (FIT) system. Here, to construct thioether-closed peptide library, a ClAc^L^-Y-tRNA^Met^_CAU_ is introduced to the ‘ATG’ codon using a methionine-free FIT system. The resulting peptides with ClAc^L^-Y at the *N*-terminal lead to spontaneous cyclization by interacting with a cysteine in the peptide, producing a macrocyclic peptide library. Following this, the peptide-mRNA library is reverse transcribed to peptide-cDNA library for subsequent selections. In selection process, the beads pre-immobilized with protein targets serves as a screening platform, exclusively retaining peptides that can bind to the proteins. The selected peptide-cDNA conjugates are subsequently eluted from magnetic beads, amplified by PCR, and the resulting DNAs undergo transcription and ligation with puromycin for the next round of selection.


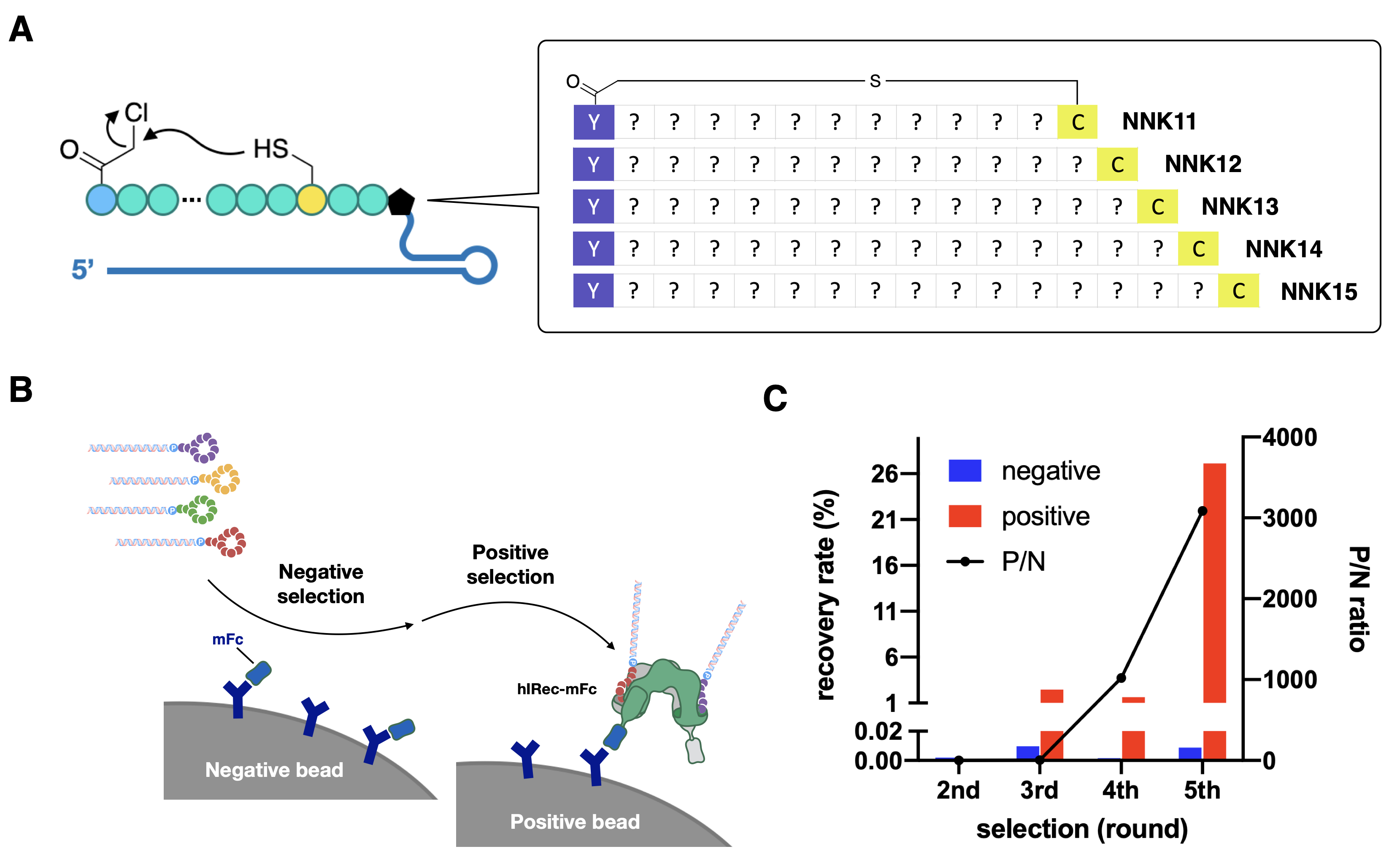


**Figure S4.** Library design. (A) Design of NNK11-NNK15 peptide library. Thioether-closed peptides, featuring random amino acid sequences ranging from 11-mer to 15-mer, were prepared for the first round of RaPID scanning. (B) *in vitro* selection scheme with negative and positive selections. In the negative selection step, mixtures of Fc-bound and Fc-free beads were incubated with the peptide-cDNA library. Subsequently, supernatants from the negative selection were incubated with positive beads pre-immobilized with hIRec-mFc. The selected peptide-cDNA conjugates on negative and positive beads were eluted for further amplification and analysis. (C) Assessment of the recovery rate of peptide-cDNA conjugates and the positive-to-negative ratio (P/N ratio) throughout each round of the RaPID selection. The recovery rate was calculated by dividing the quantity of peptide-cDNA conjugates retrieved from negative or positive beads by the total amount of peptide-cDNA conjugates before the selection process.


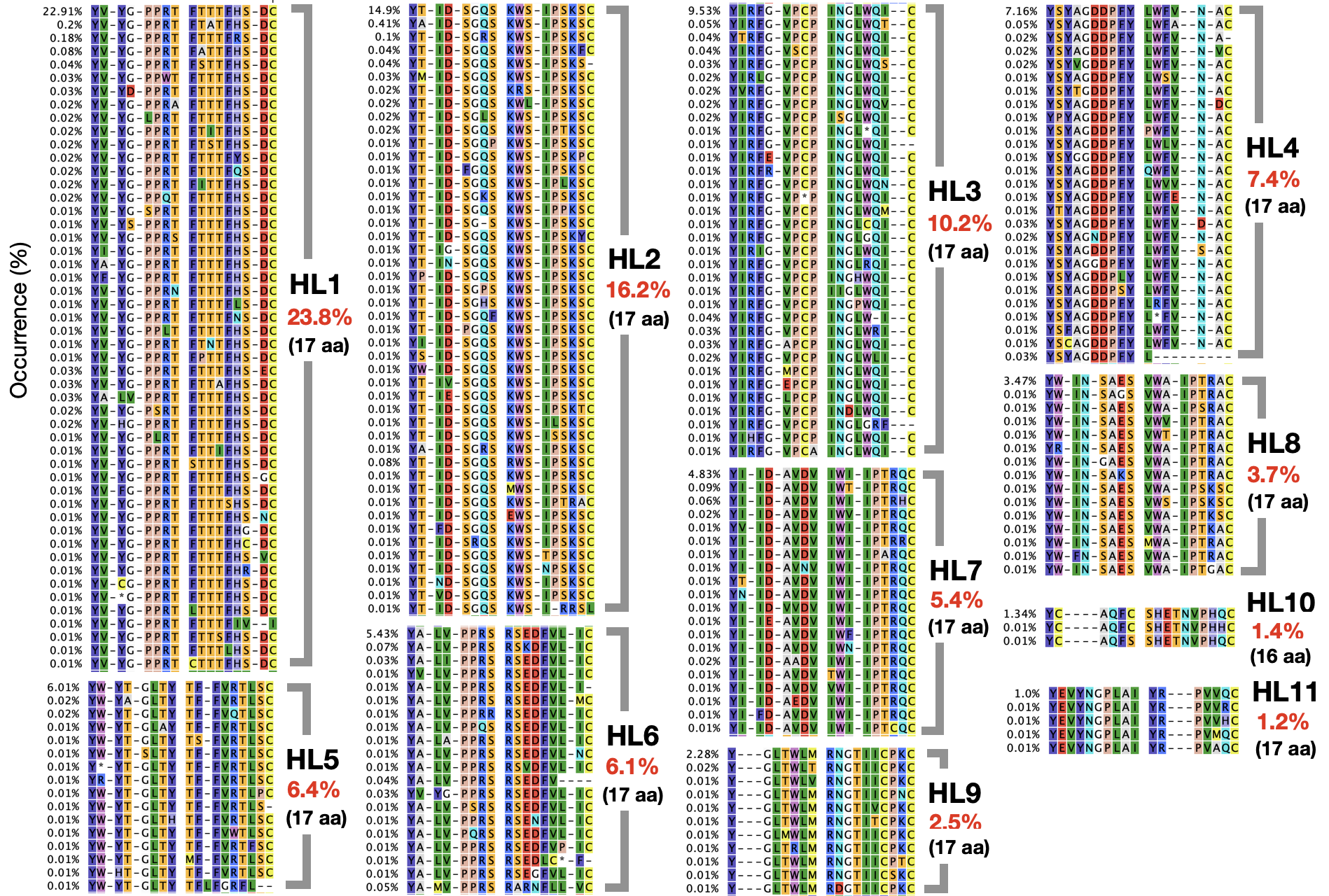


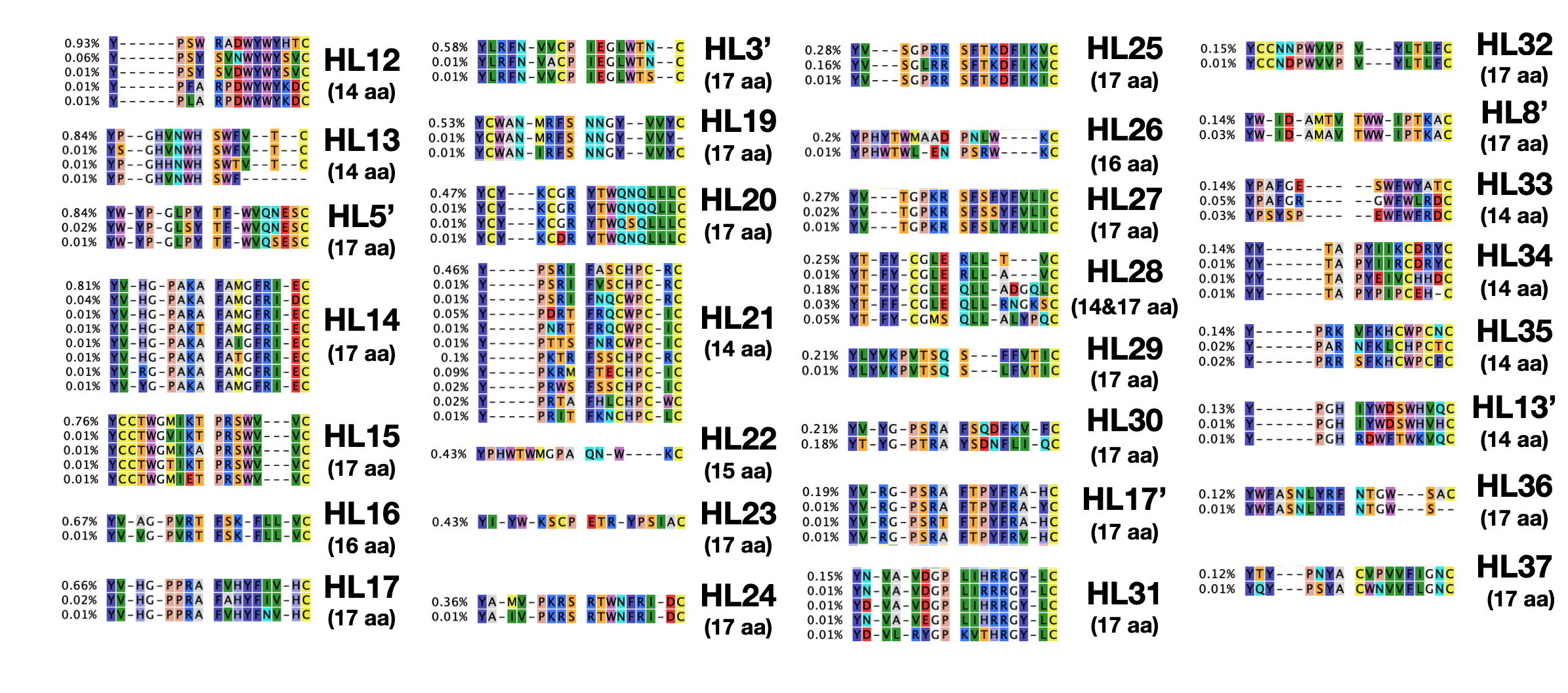


**Figure S5.** The next-generation sequencing (NGS) results from the 5th round of the RaPID selection against hIRec dimers. Sequence alignments were conducted using CLC Sequence Viewer with parameters set at gap open cost = 20 and gap extension cost = 1. Sequences with occurrences below 0.01% were excluded from the alignments. Notably, for HL1 to HL11, the overall occurrences within the same family were summarized and are highlighted in red.


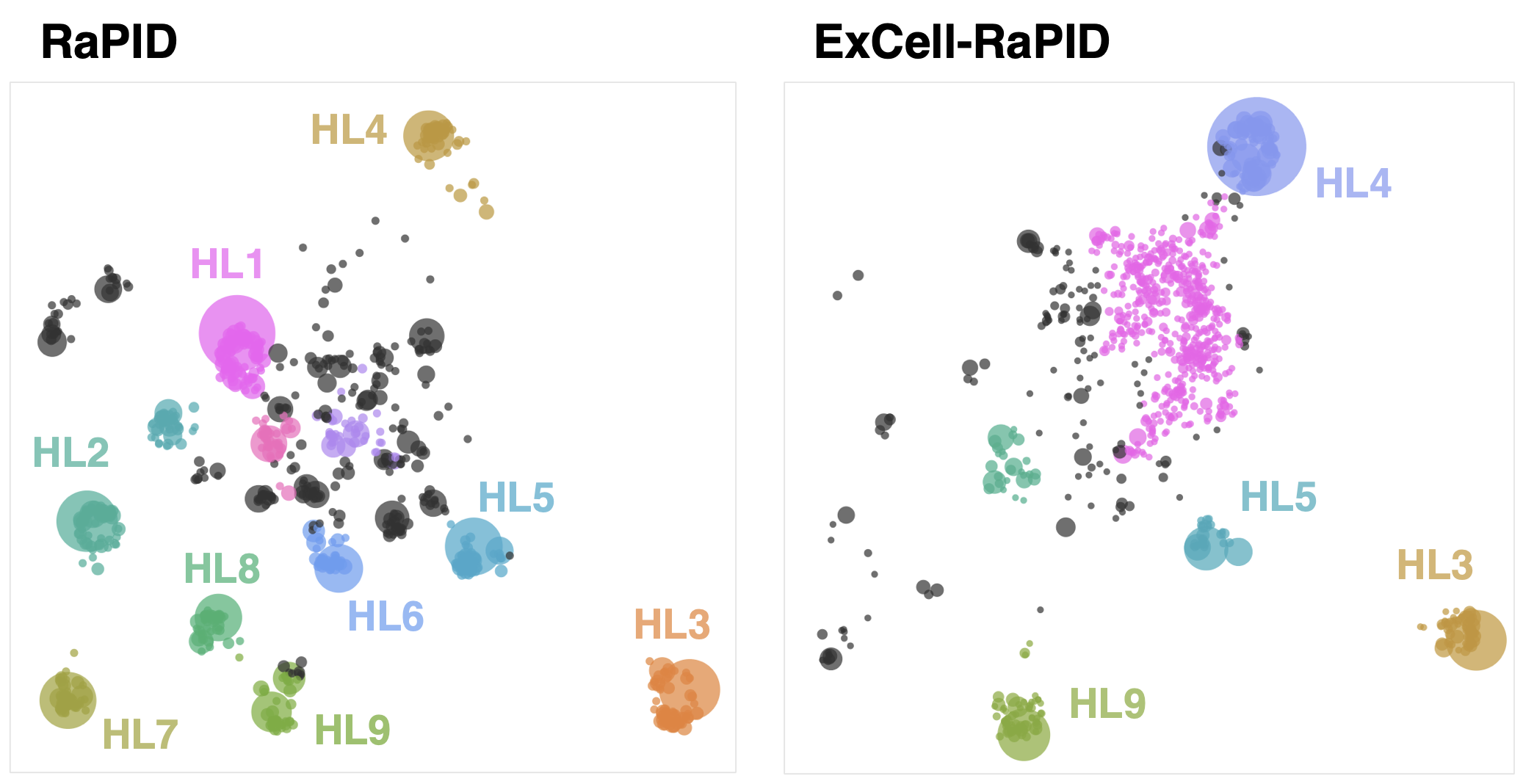


**Figure S6.** UMAP analysis of RaPID and RaPID-ExCells selections in the 5th round. Clusters were determined using HDBSCAN, with colors indicating their corresponding cluster family. The size of the circles represents the occurrence of each sequence.


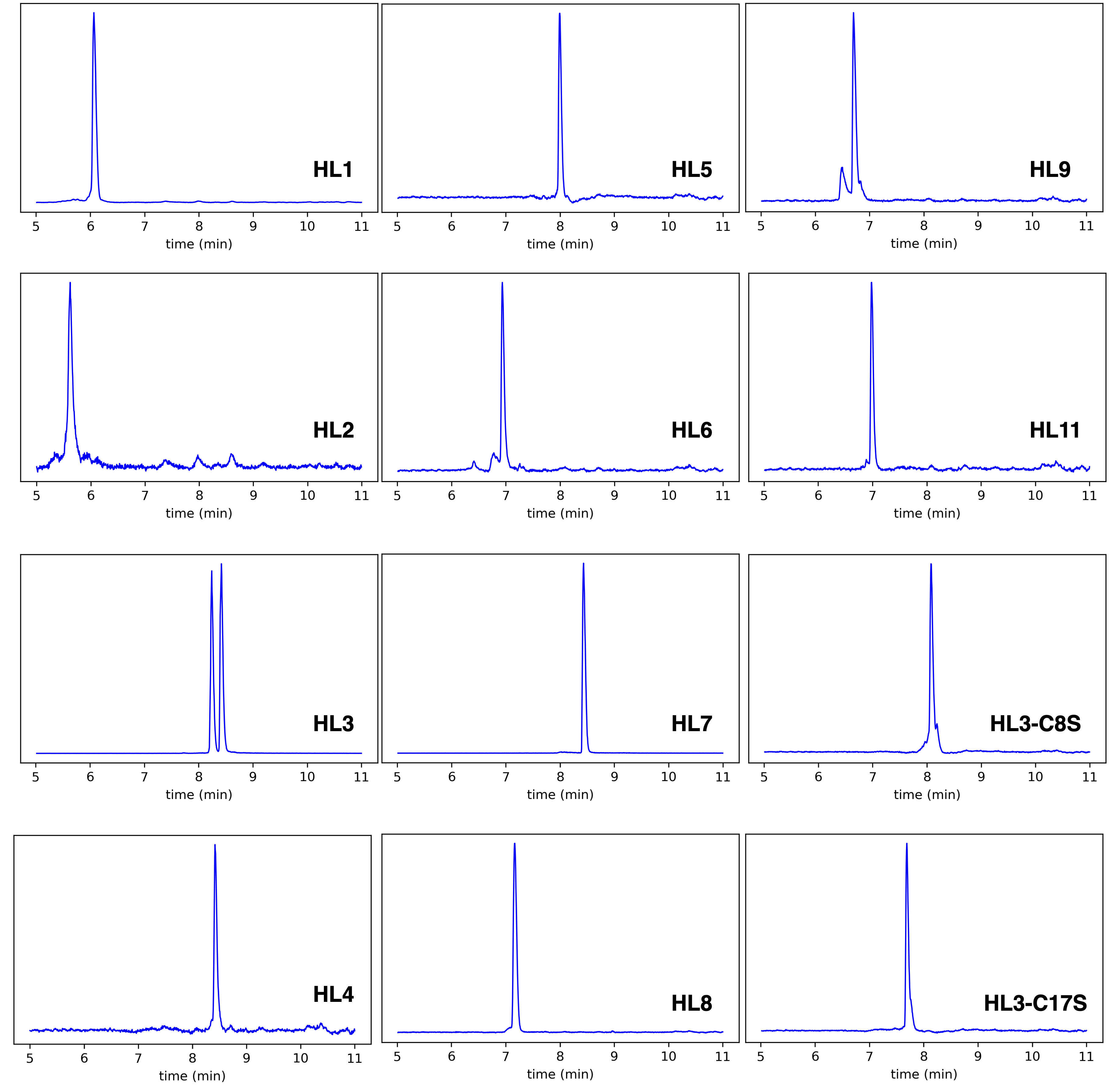


**Figure S7.** Chromatogram of purified cyclic peptides. All chromatograms were subtracted by the blank. HL3 exhibited two distinct peaks, with the first being cyclized through the cysteine at position 8, and the second being cyclized through the cysteine at position 17.


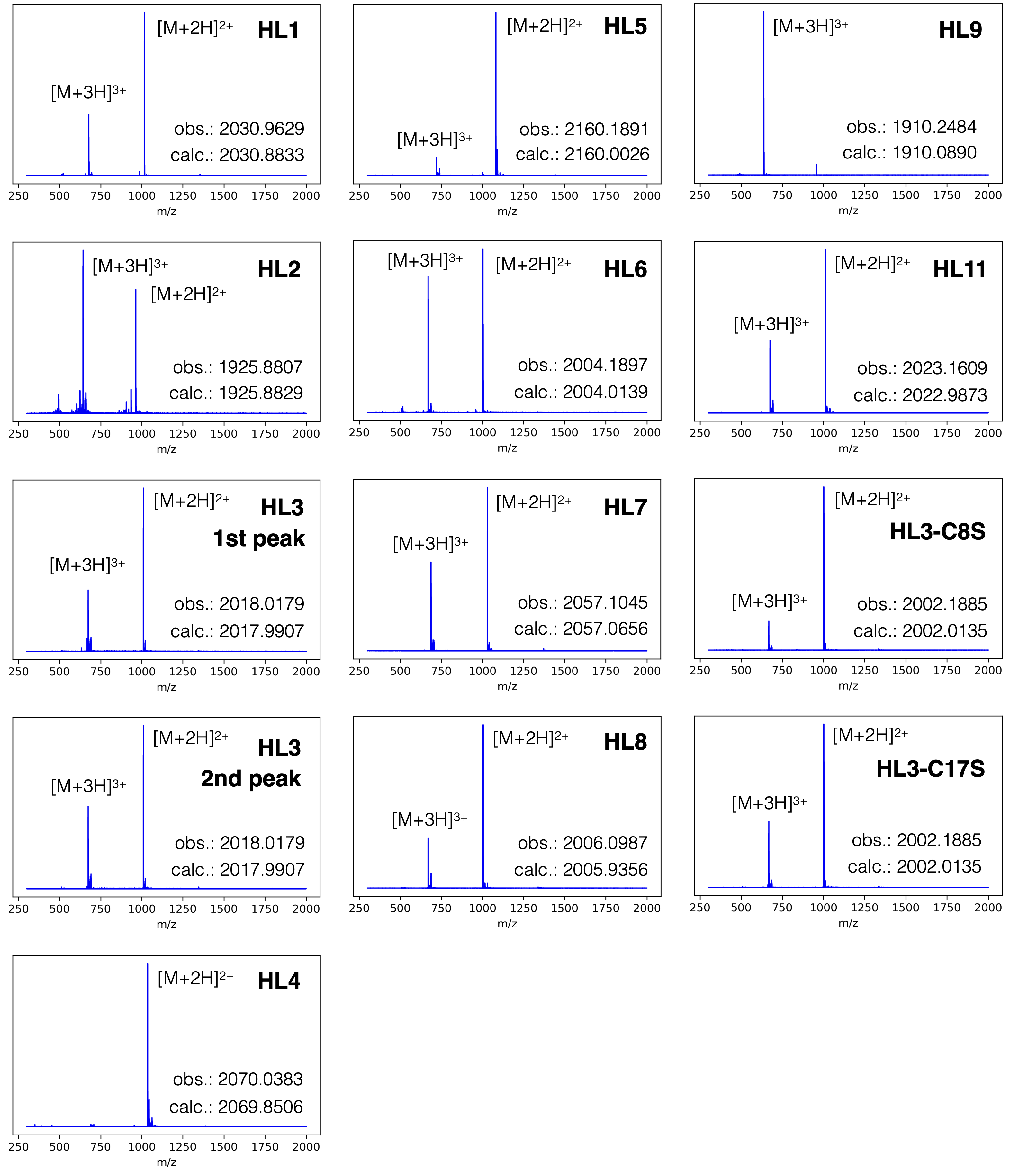


**Figure S8.** ESI-MS analysis of purified cyclic peptides using LC/MS. The major peaks of the peptides in the Fig. S4 were analyzed. The observed mass of each peptide was derived from the composite extracted-ion chromatogram. obs.: observed mass; calc.: calculated mass.


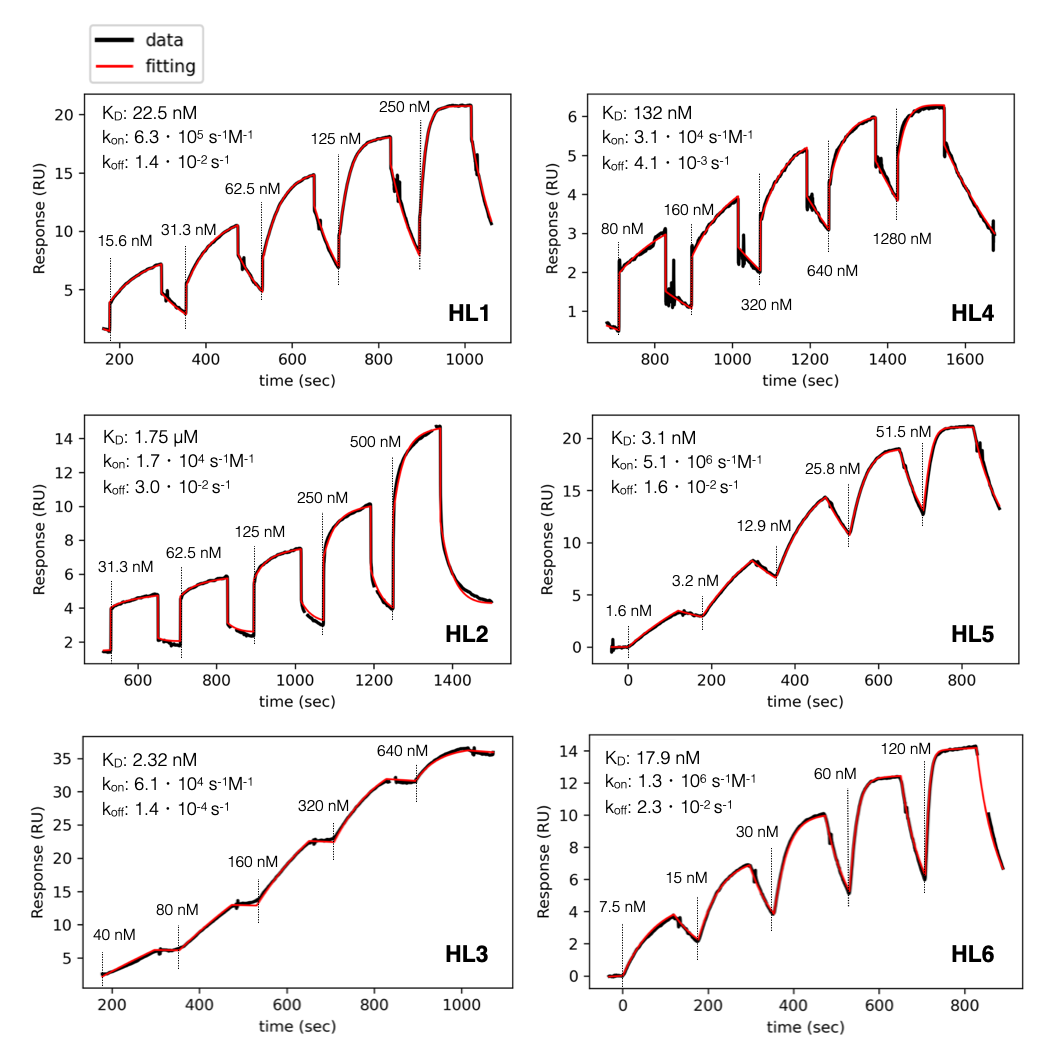


**Figure S9.** Surface plasmon resonance (SPR) sensorgrams for cyclic peptides, HL1, HL2, HL3, HL4, HL5 and HL6. Ligands with indicated concentrations were flew through Protein G chip immobilized with hIRec-mFc dimers for SPR measurement. Affinity equilibrium constant (K_D_), association kinetic constant (k_on_) and dissociation kinetic constant (k_off_) were determined by fitting with 1:1 binding model. Experimental data are shown in black and the best fitting curves are shown in red.


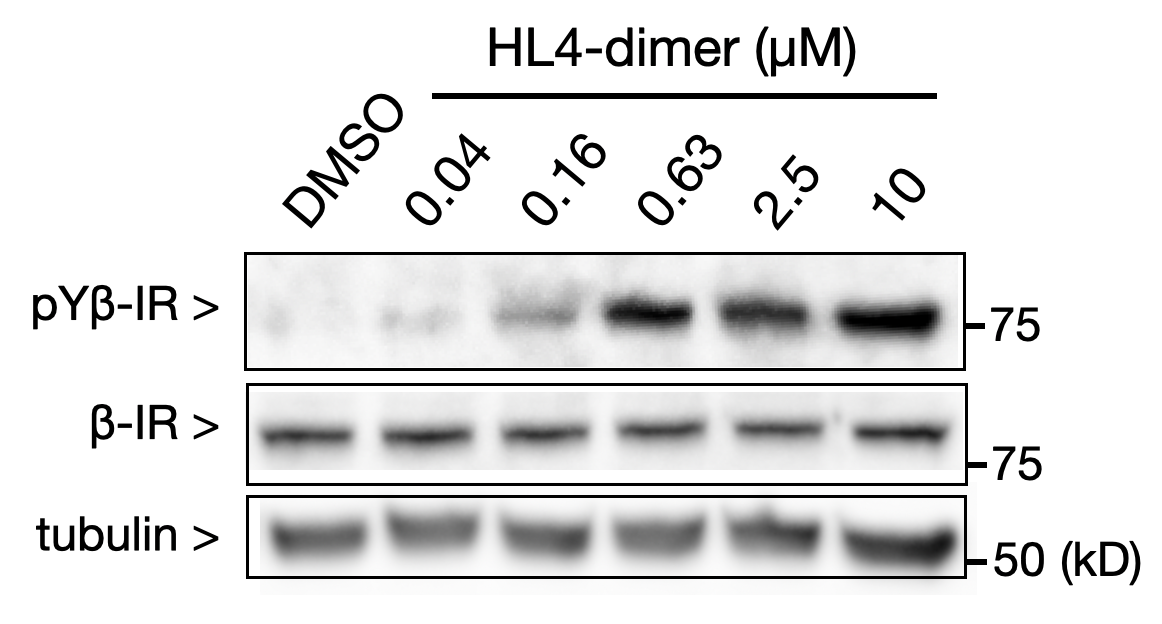


**Figure S10.** Dose-responsive curve of peptide-stimulated phosphorylation of IR quantified by Western blotting. The reaction was carried out at 37°C for 10 minutes.


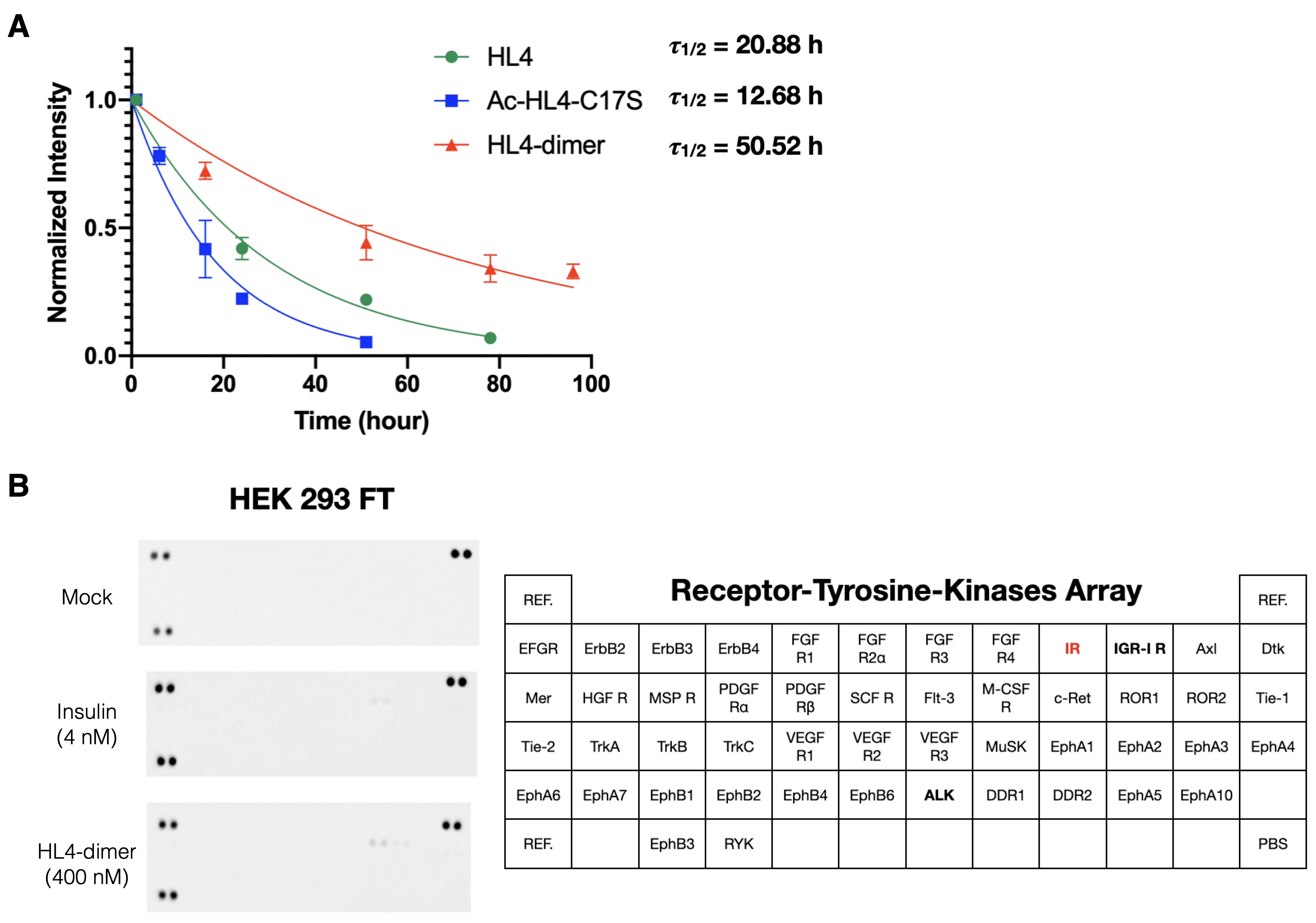


**Figure S11.** Serum stability and on-target specificity of HL4 peptides. (A) Serum stability of HL4, Ac-HL4-C17S, and HL4-dimer. Peptides were treated with human serum at 37°C in the presence of an internal standard for the corresponding time intervals. Quantification of the remaining amounts of peptides was assessed at various time points by LC/MS. (B) Receptor tyrosine kinase (RTK) phosphorylation profiling in HEK293FT cells. HEK293FT cells were treated with vehicle (Mock), insulin (4 nM), or HL4-dimer (400 nM) and incubated at 37 °C for 10 min. Cell lysates were analyzed using the Proteome Profiler™ Human Phospho-RTK Array Kit according to the manufacturer’s instructions. Representative array images are shown. The positions of individual RTKs on the array membrane are indicated in the schematic on the right. IR, insulin receptor.

**
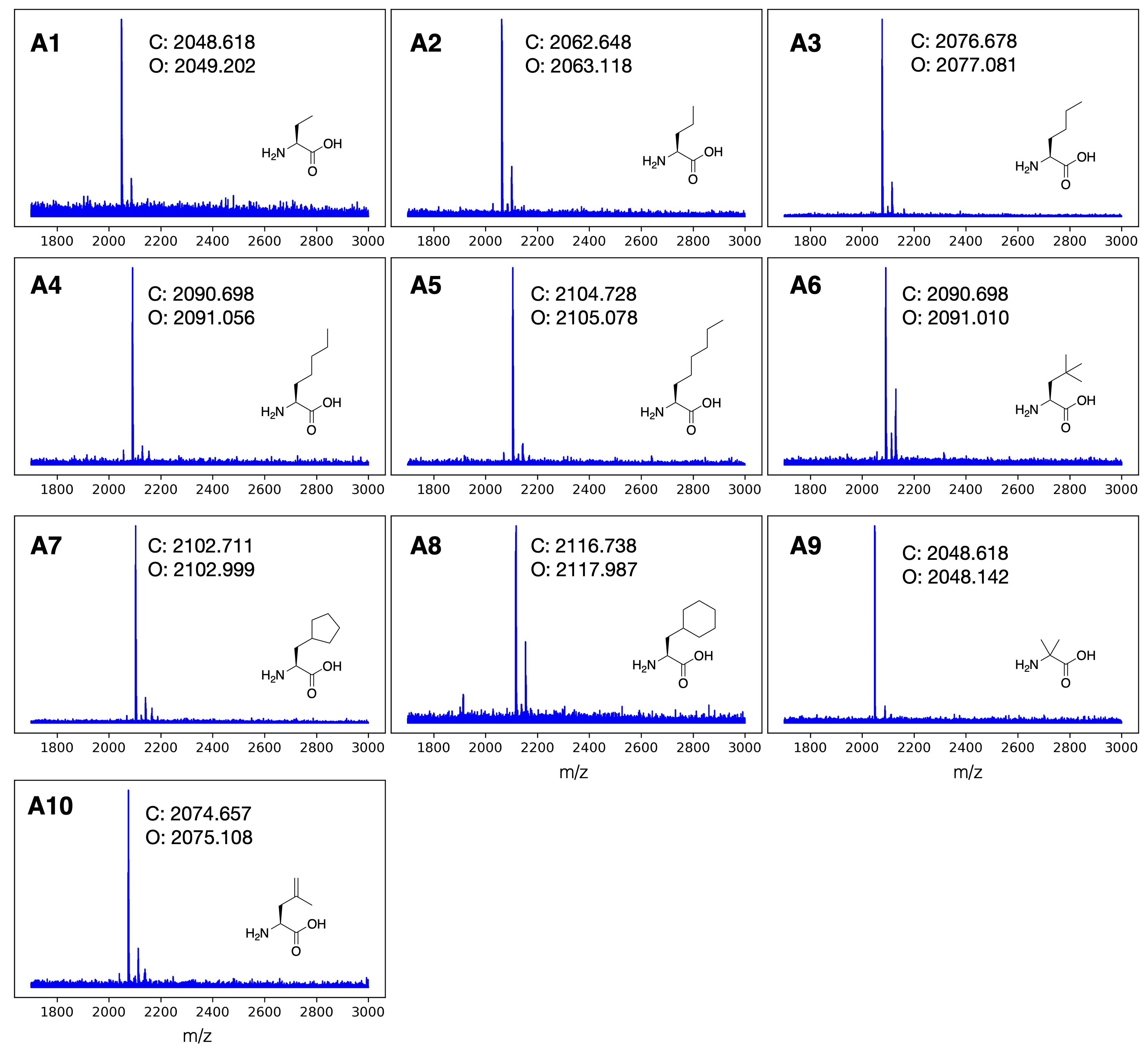
**

**Figure S12.** MALDI-TOF spectra of translation products for each reprogrammed genetic code with non-proteinogenic aliphatic amino acids. The aliphatic amino acids (A1-A10), as shown in the inset, were introduced to the ‘AUG’ elongation codon for translation. The observed mass of each peptide was derived from the composite extracted-ion chromatogram. O: observed mass; C: calculated mass.

**
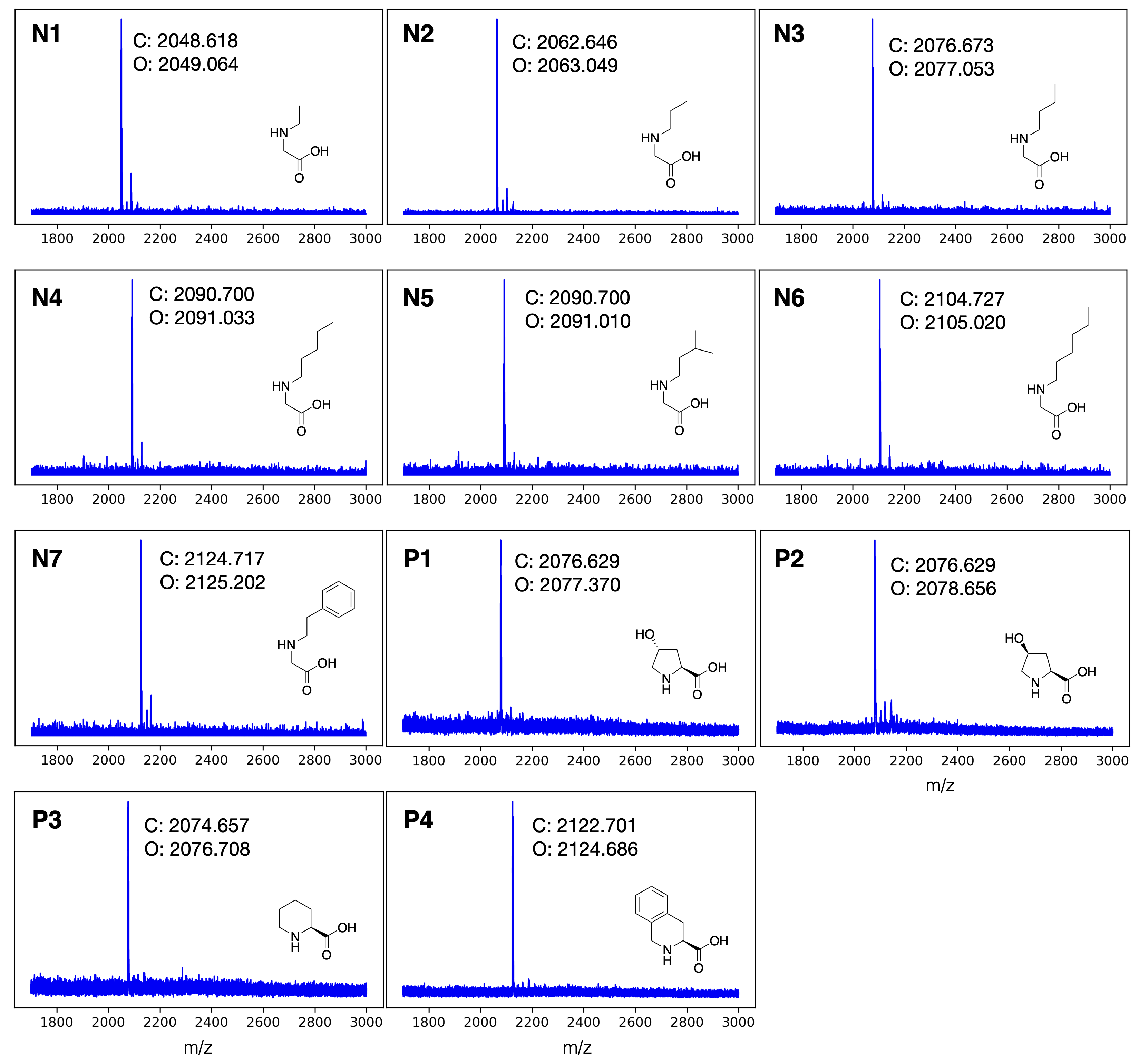
**

**Figure S13.** MALDI-TOF spectra of translation products for each reprogrammed genetic code with non-proteinogenic peptoids and proline-analogues. The peptoids (N1-N7) and proline-analogues (P1-P4), as shown in the inset, were introduced to the ‘AUG’ elongation codon for translation. The observed mass of each peptide was derived from the composite extracted-ion chromatogram. O: observed mass; C: calculated mass.


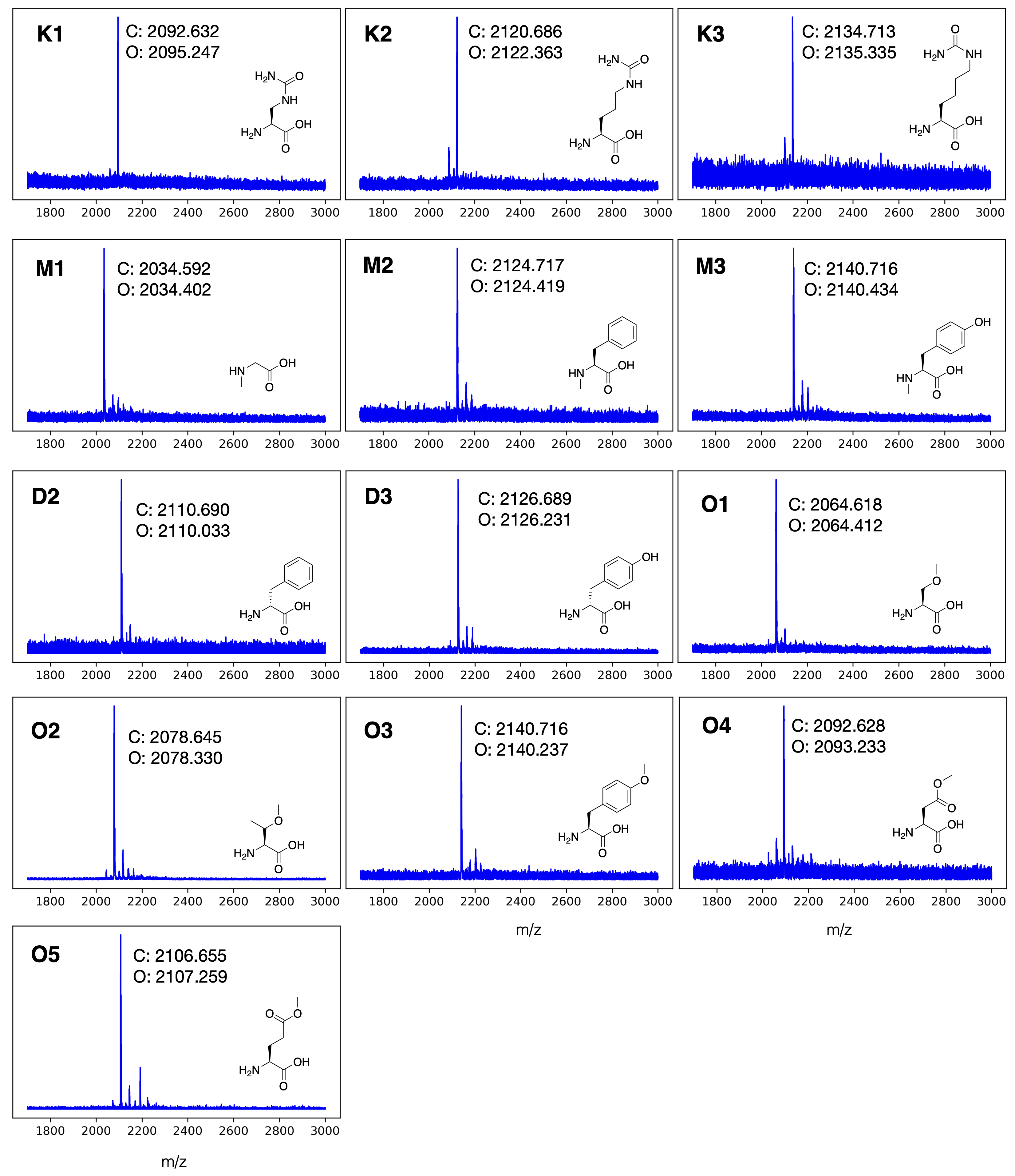


**Figure S14.** MALDI-TOF spectra of translation products for each reprogrammed genetic code with various non-proteinogenic amino acids. The lysine-analogues (K1-K3), D-amino acids (D2-D3), N-methyl amino acids (M1-M3), and O-methyl amino acids (O1-O5), as shown in the inset, were introduced to the ‘AUG’ elongation codon for translation. The observed mass of each peptide was derived from the composite extracted-ion chromatogram. O: observed mass; C: calculated mass.

**
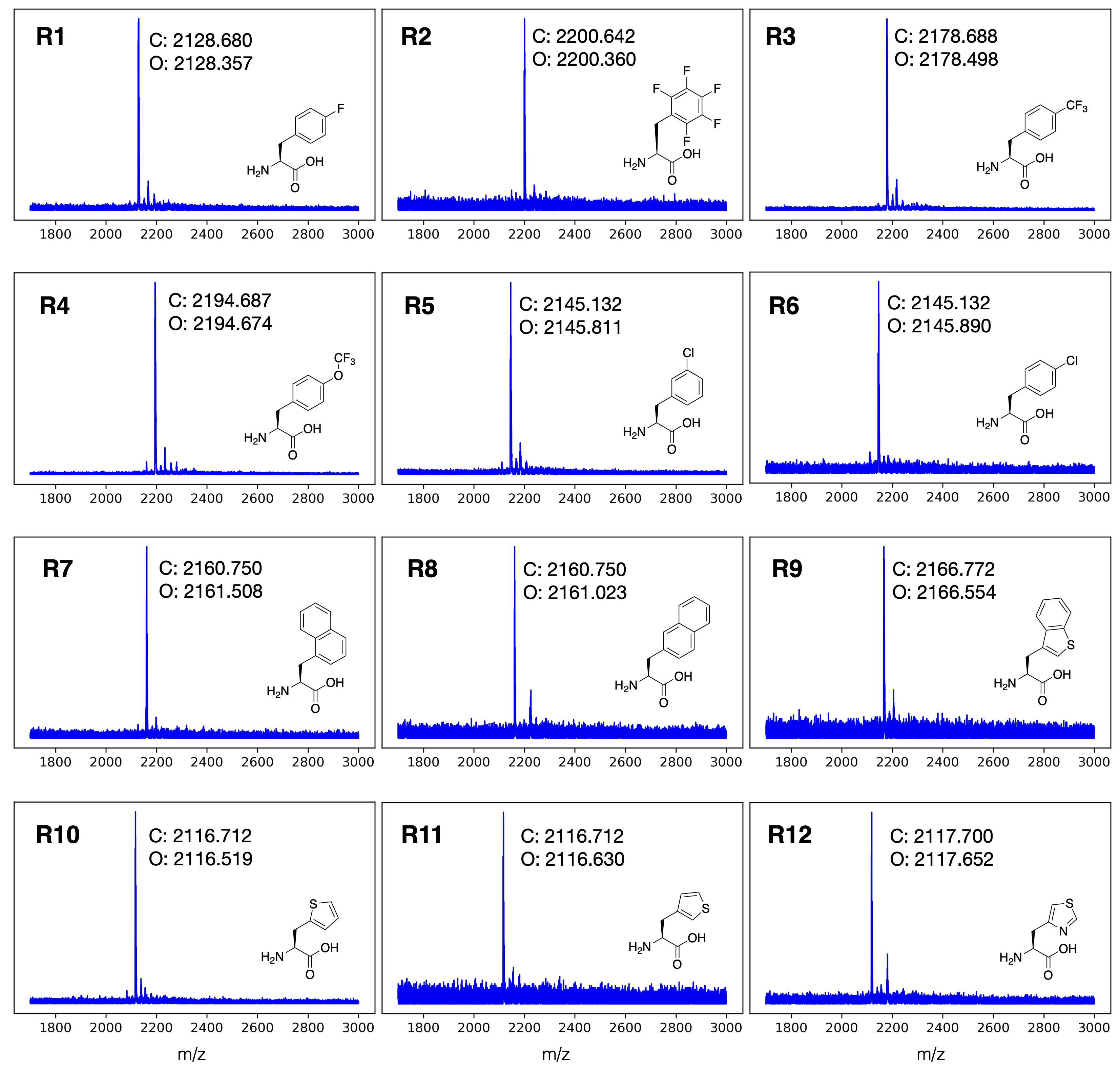
**

**Figure S15.** MALDI-TOF spectra of translation products for each reprogrammed genetic code with aromatic non-proteinogenic amino acids. The aromatics (R1-R12), as shown in the inset, were introduced to the ‘AUG’ elongation codon for translation. The observed mass of each peptide was derived from the composite extracted-ion chromatogram. O: observed mass; C: calculated mass.

**
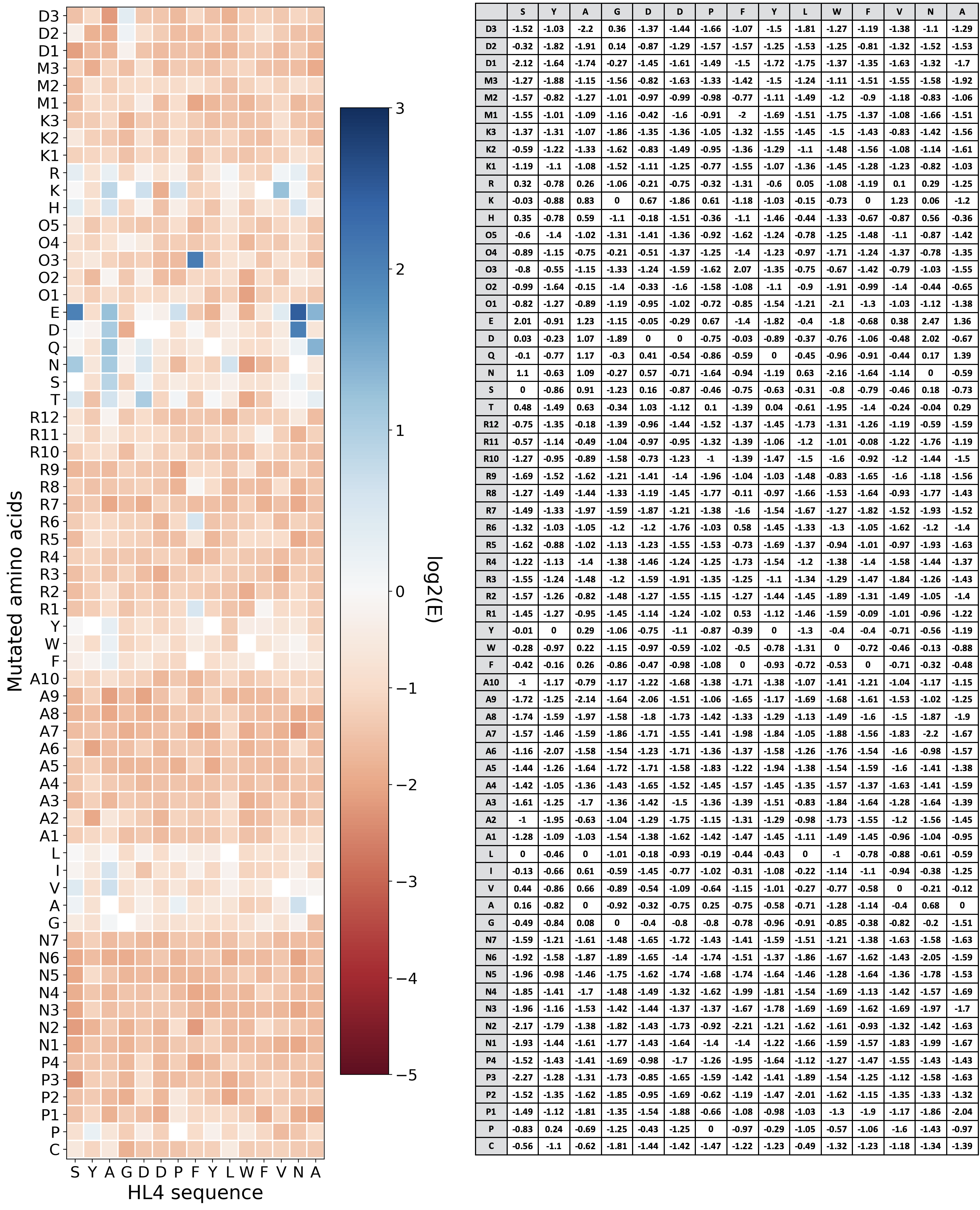
**

**Figure S16.** Overall view of site-saturation mutagenesis through HL4. The scanning was conducted in triplicate, and the displayed values represent the average Log2(*E*) values, as shown on the right.

**
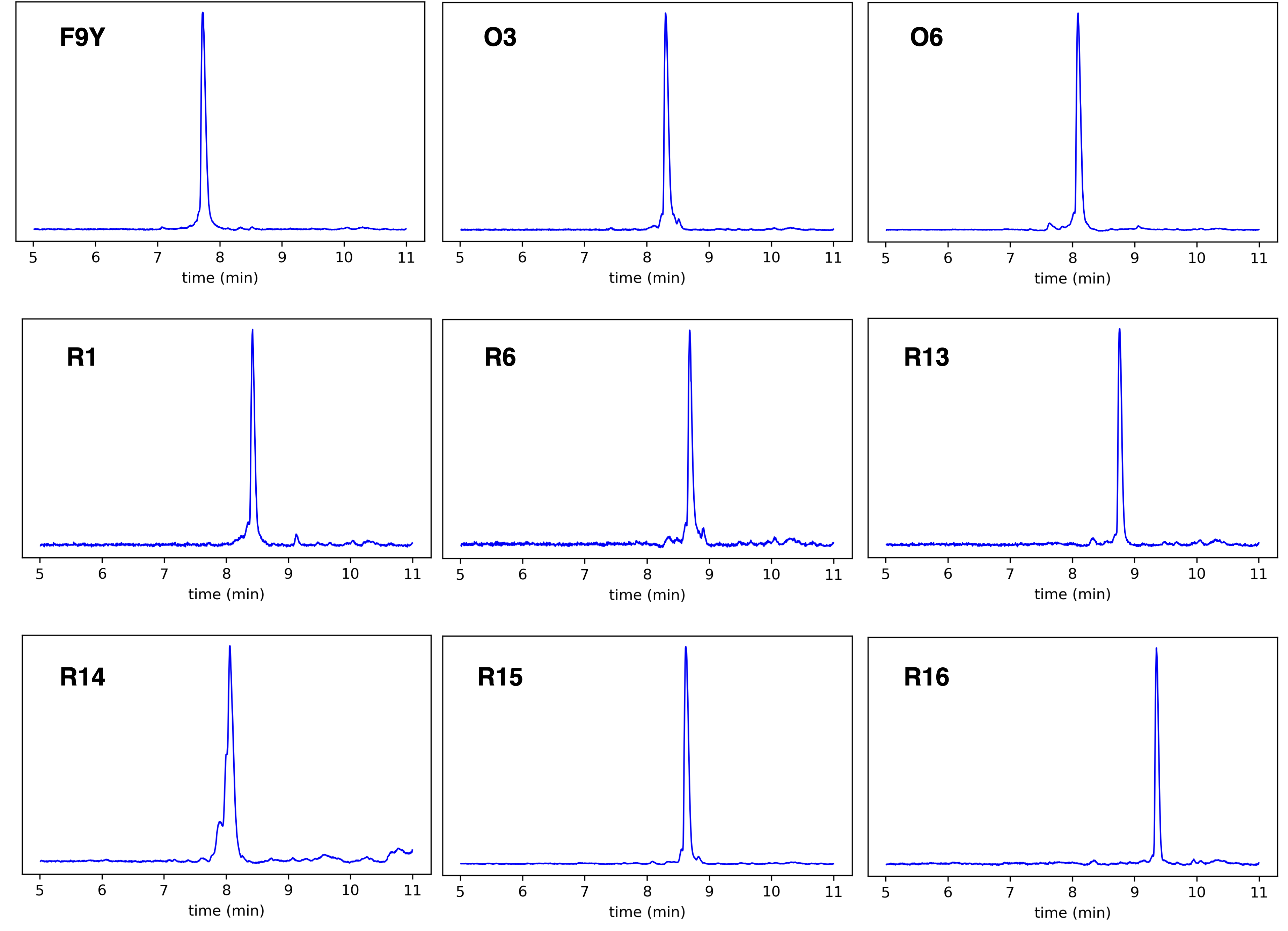
**

**Figure S17.** Chromatogram of purified HL4 mutants with incorporation of non-proteinogenic aromatic amino acids. Each chromatogram has been baseline-corrected by subtracting the blank, providing clear visualization of the purified HL4 mutants with non-proteinogenic aromatic amino acids incorporated.

**
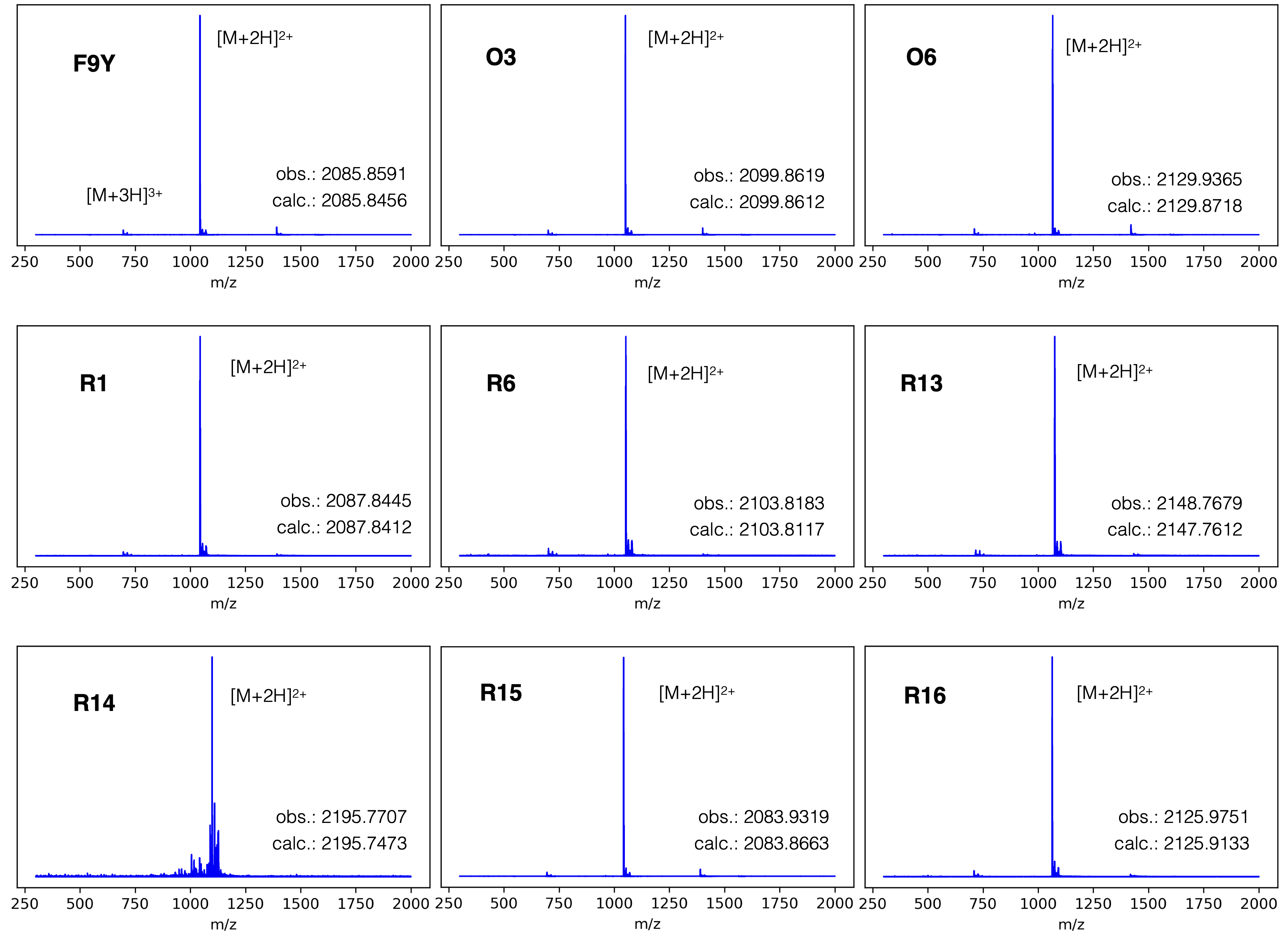
**

**Figure S18.** ESI-MS analysis of purified HL4 mutants with incorporation of non-proteinogenic aromatic amino acids. The main peaks of the peptides in the Fig. S21 were analyzed. The observed mass of each peptide was derived from the composite extracted-ion chromatogram. obs.: observed mass; calc.: calculated mass.

**
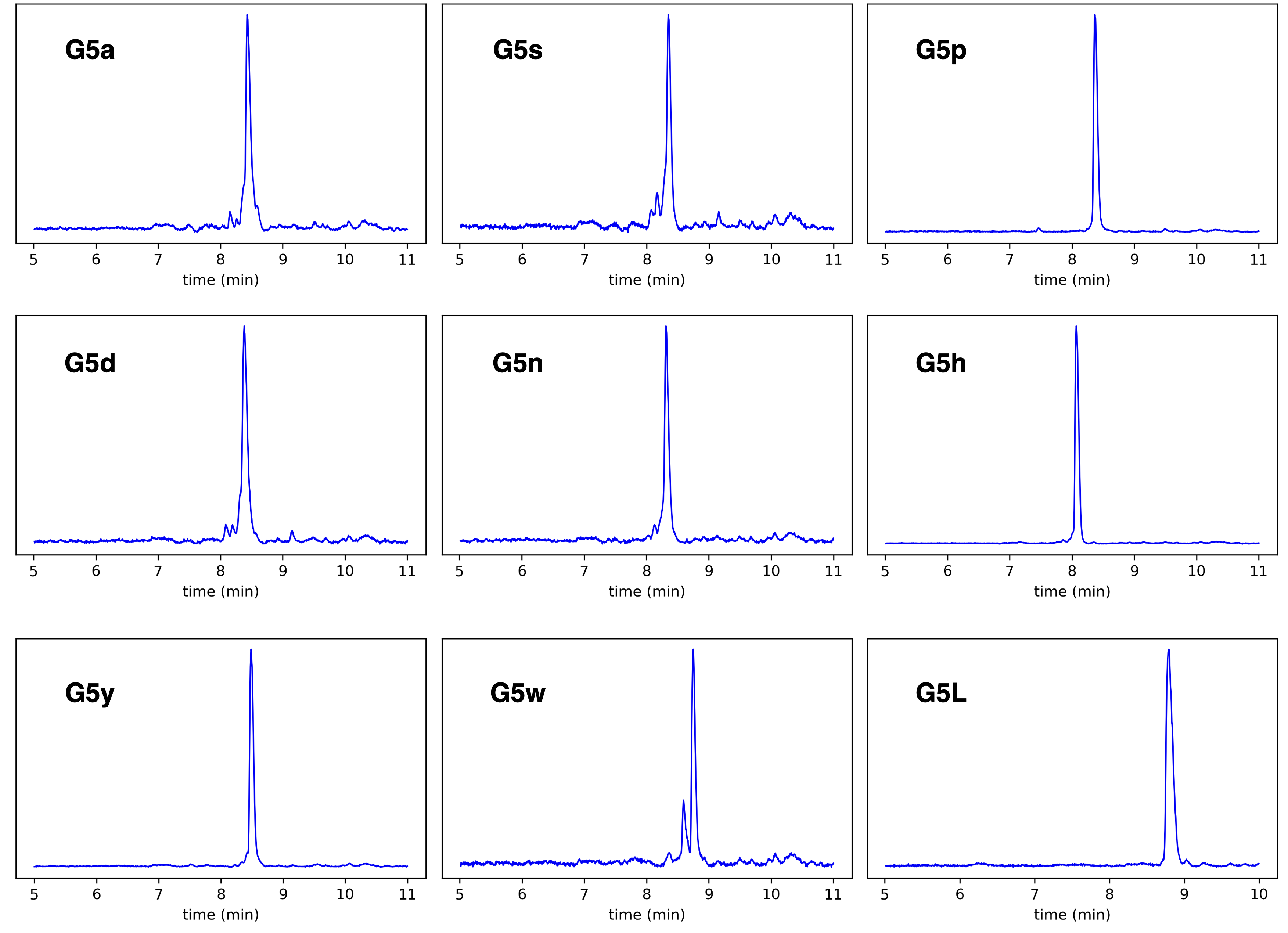
**

**Figure S19.** Chromatogram of purified HL4 mutants with incorporation of D-amino acids. Each chromatogram has been baseline-corrected by subtracting the blank, providing clear visualization of the purified HL4 mutants with non-proteinogenic aromatic amino acids incorporated.

**
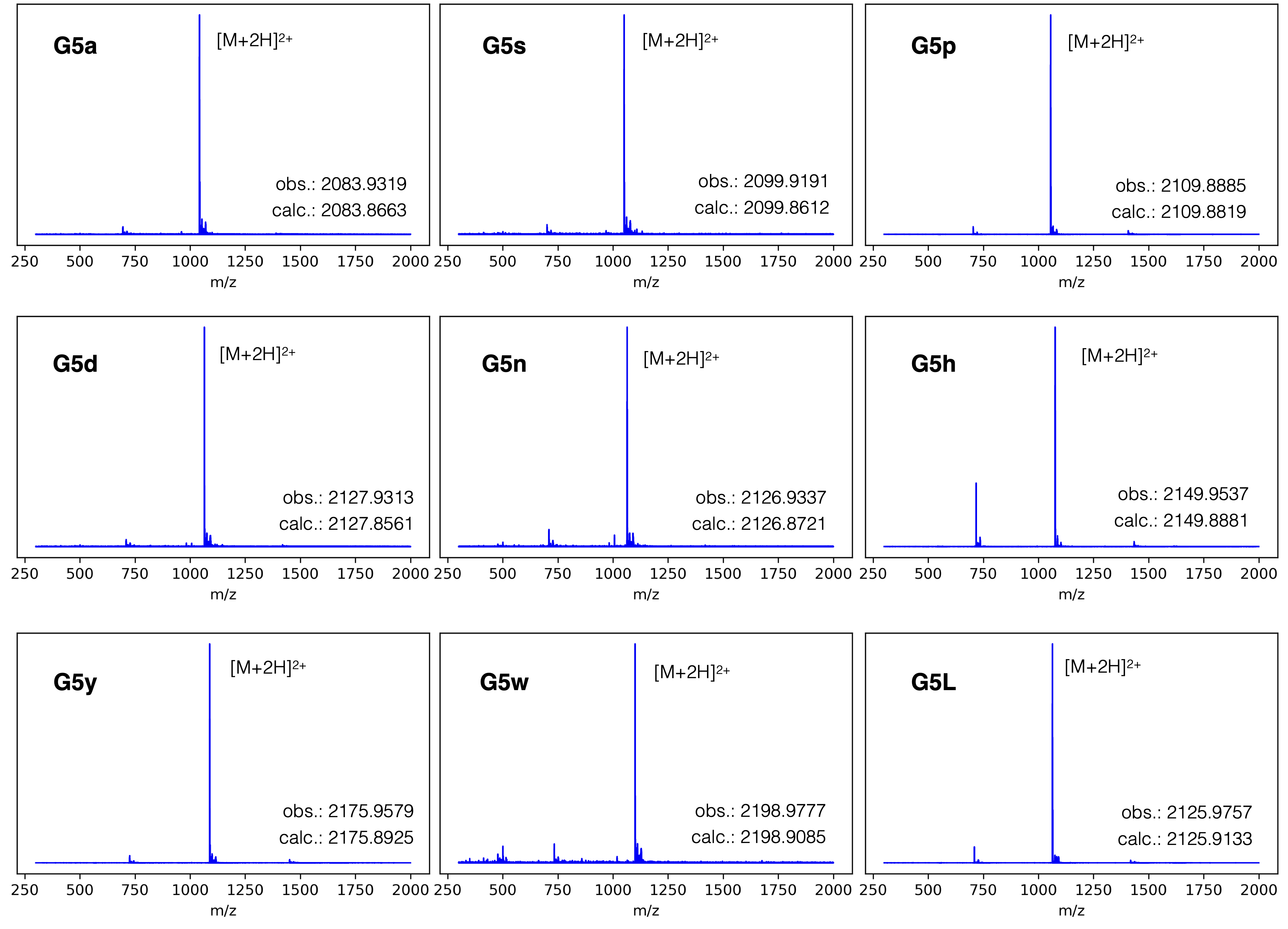
**

**Figure S20.** ESI-MS analysis of purified HL4 mutants with incorporation of D-amino acids. The main peaks of the peptides in the Fig. S23 were analyzed. The observed mass of each peptide was derived from the composite extracted-ion chromatogram. obs.: observed mass; calc.: calculated mass.

**
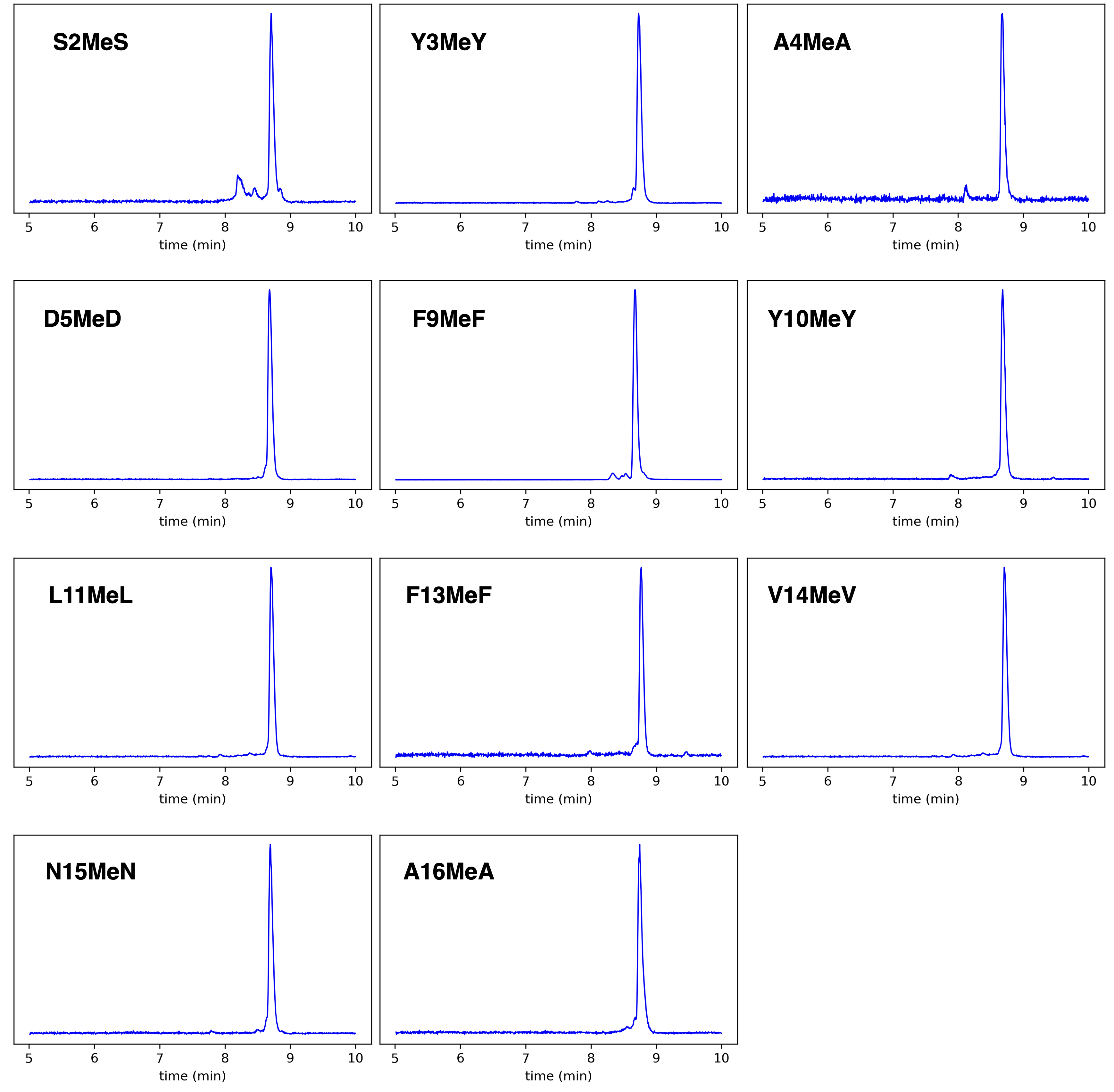
**

**Figure S21.** Chromatogram of purified HL4 mutants with incorporation of N-methyl amino acids. Each chromatogram has been baseline-corrected by subtracting the blank, providing clear visualization of the purified HL4 mutants with non-proteinogenic aromatic amino acids incorporated.

**
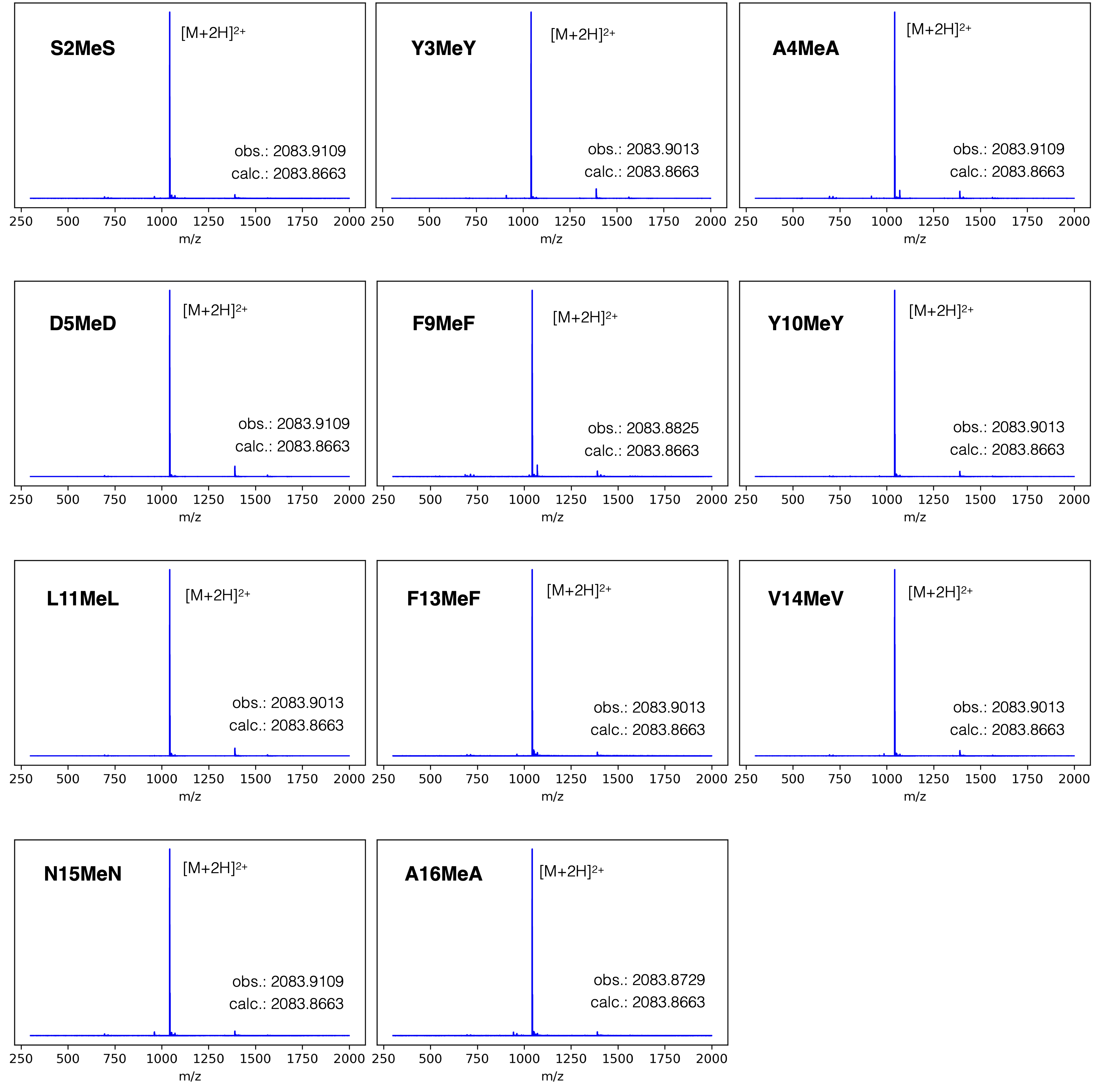
**

**Figure S22.** ESI-MS analysis of purified HL4 mutants with incorporation of N-methyl amino acids. The main peaks of the peptides in the Fig. S25 were analyzed. The observed mass of each peptide was derived from the composite extracted-ion chromatogram. obs.: observed mass; calc.: calculated mass.

**
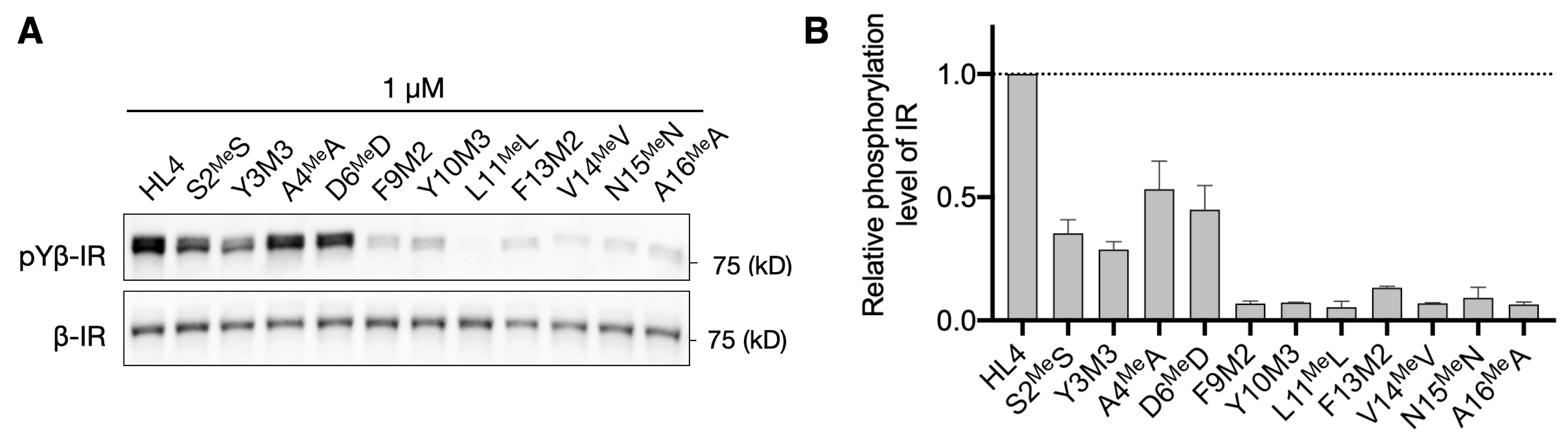
**

**Figure S23.** Agonist activity of HL4 mutants with introduction of N-methyl amino acids. (A) Auto-phosphorylation of pYβ-IR induced by HL4 mutants with incorporation of N-methyl amino acids. The original amino acid, denoted as X, was replaced by N-methyl amino acid, MeX, to examine its impact on auto-phosphorylation. Auto-phosphorylation of pYβ-INSR was induced by 1 µM of peptides at 37 ℃ for 5 min. (B) Quantification of the western blot data presented in panels A. Each experiment was conducted in triplicate. Data are presented as mean ± SD from n = 3 independent experiments.

**
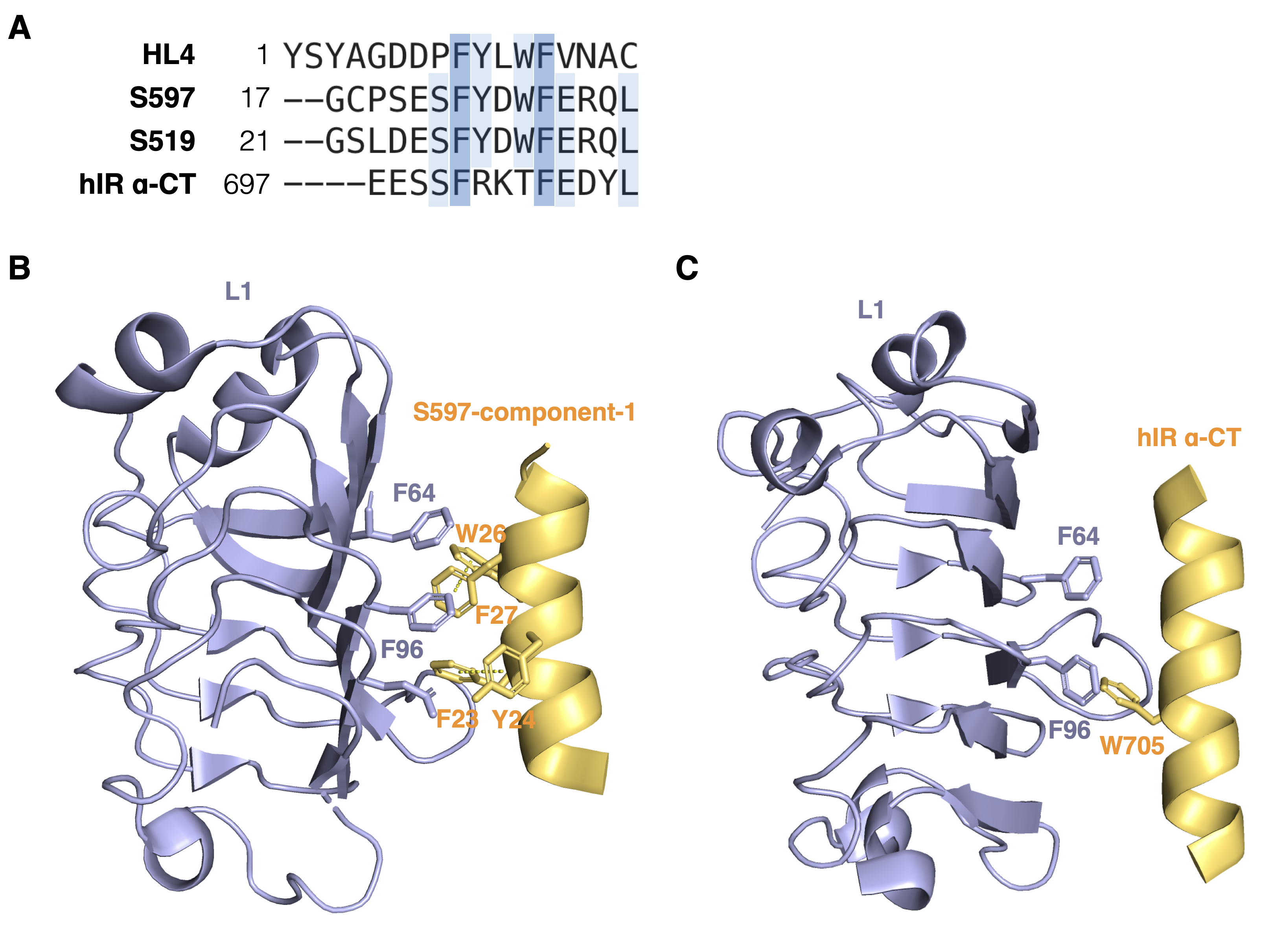
**

**Figure S24.** Comparison with S597 peptide. (A) Sequence similarity between HL4, S597 (residue 17-31), S519 (residue 21-35) and hIR ɑ-CT (residue 697-709). (B) The structure of L1 domain of IR binding to S597-component-1 (residue 18-31). PDB: 5J3H. (C) The structure of L1 domain of IR binding to the hIR ɑ-CT (residue 698-714). PDB: 6PXV.


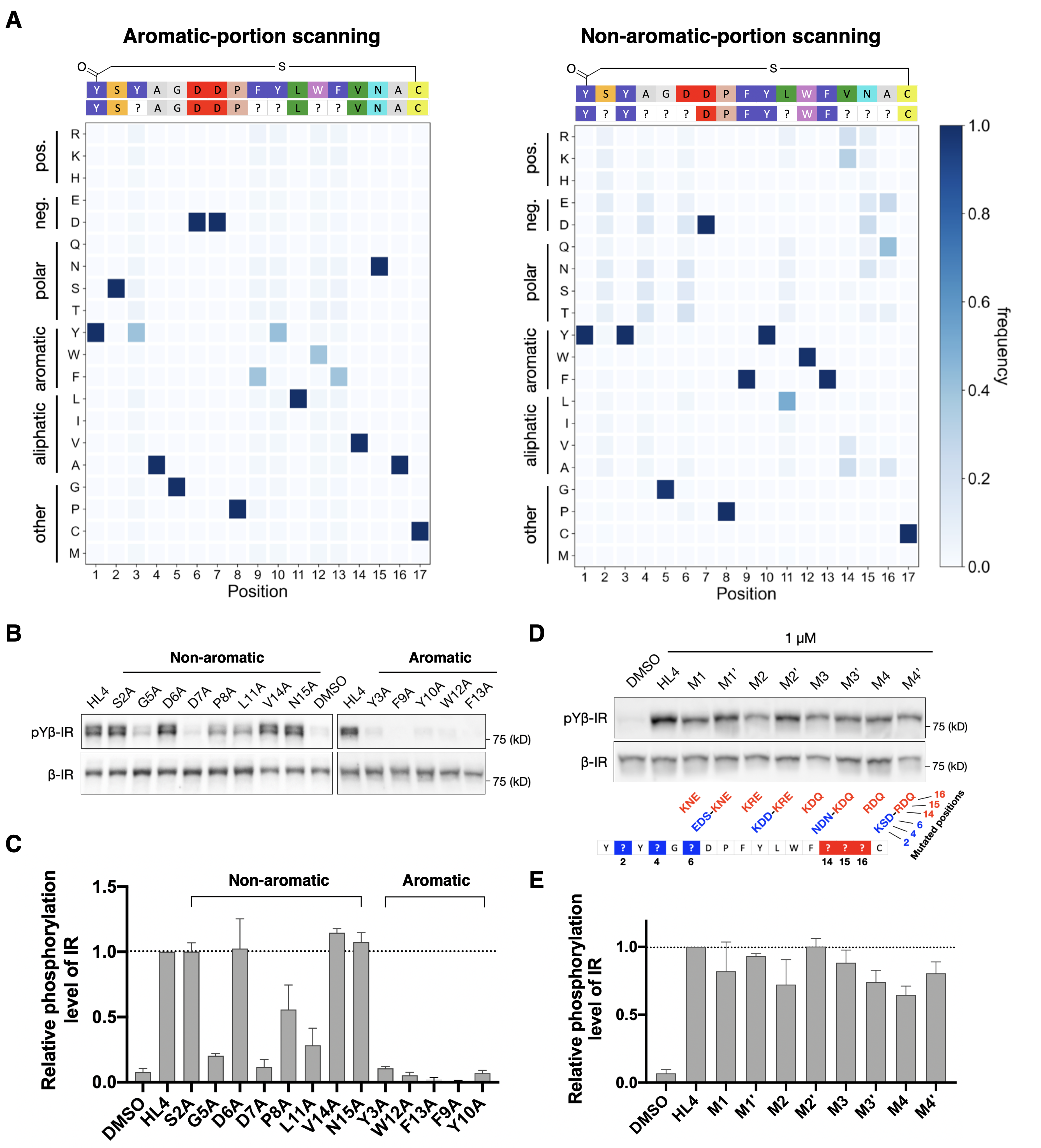


**Figure S25.** Focused library scanning using ExCell-RaPID. (A) Focused libraries designed with random sequences at the aromatic and nonaromatic portions using ExCell-RaPID. The aromatic-portion scanning introduced random proteinogenic amino acids to the aromatic regions (positions 3, 9, 10, 12, and 13), while the nonaromatic-portion scanning incorporated random amino acids at positions 2, 4, 5, 6, 11, 14, 15, and 16. (B-C) Auto-phosphorylation of insulin receptors induced by 1 µM of HL4 mutants with mutated at (B) the aromatic and (D) the nonaromatic regions. (C and E) Quantification of the western blot data presented in panels B and D. Each experiment was conducted in triplicate. Data are presented as mean ± SD from n = 3 independent experiments.

**
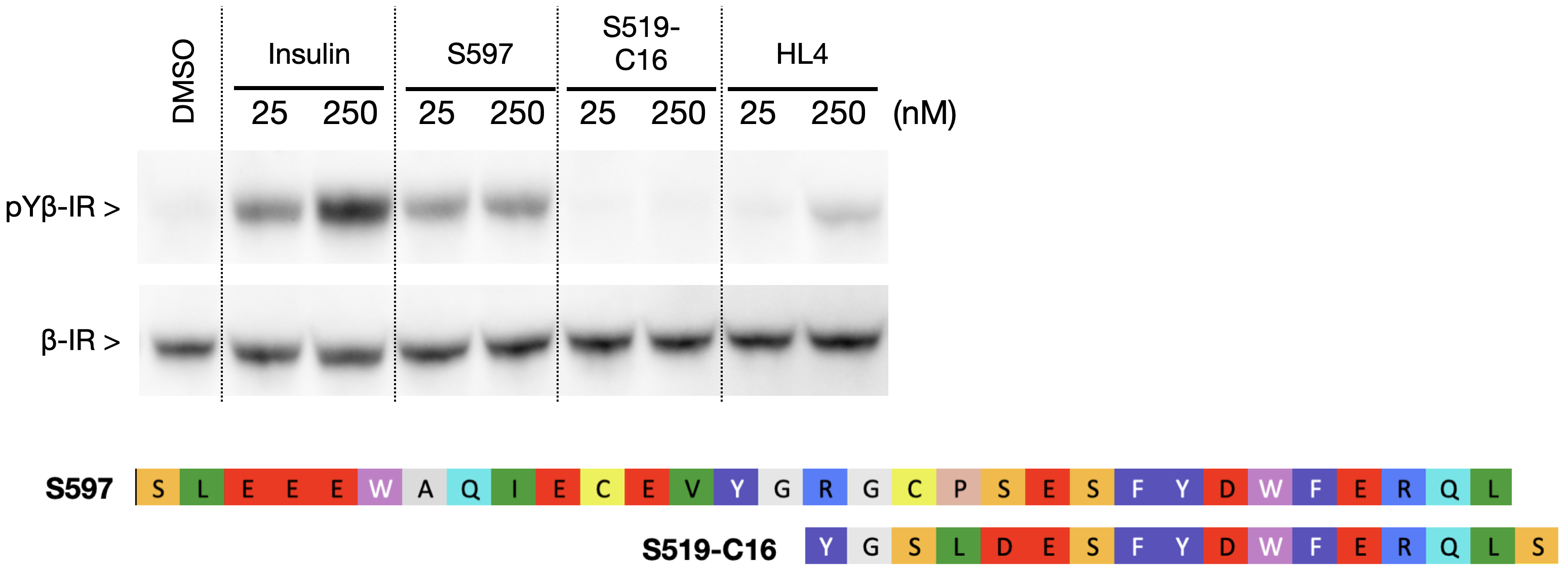
**

**Figure S26.** Auto-phosphorylation of IR induced by HL4, insulin, S597, and S597-component-1 (S519-C16). Peptides (25 nM and 250 nM) were incubated at 37 °C for 5 min to assess their effects on pYβ-INSR auto-phosphorylation. Corresponding peptide sequences are provided below the figure.

**
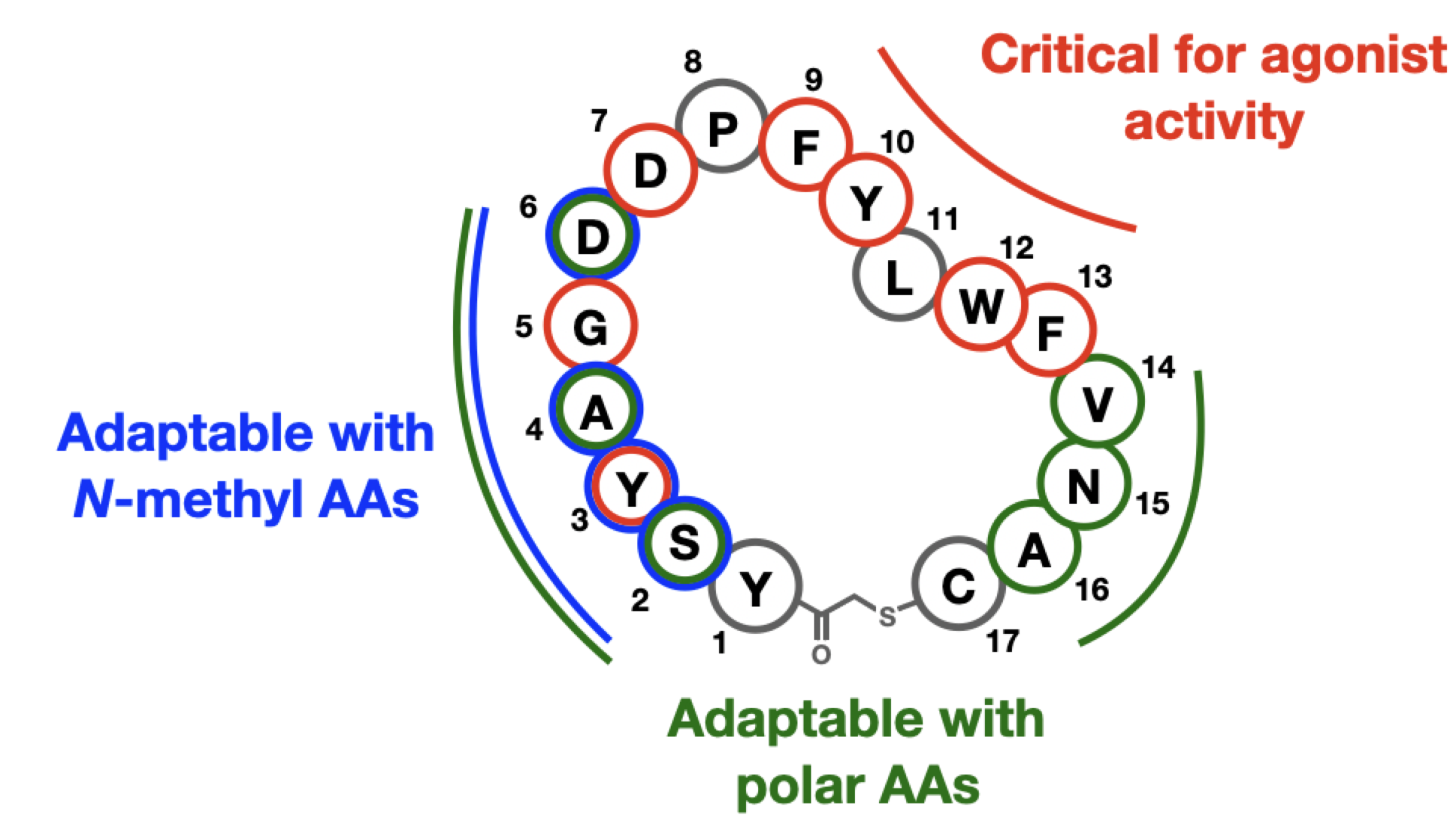

Figure S27.** Activity-Structure insights of the HL4 macrocyclic peptide. Circular schematic of HL4 highlighting residues essential for agonist activity (red), adaptable positions for N-methyl amino acid substitutions (blue), and adaptable positions for polar amino acid substitutions (green). Grey residues represent the thioether macrocyclic linker and other positions with minimal contribution to agonist potency. Aromatic residues at positions 9–13 form a conserved FYLWF motif critical for insulin receptor activation.

# Supporting Tables

## **Table S1.** Primers for construction of flexizymes and tRNAs.

| Primer | 5’-3’ sequence |
| --- | --- |
| T7ex5.F22 | GGCGTAATACGACTCACTATAG |
| eFx.R45 | ACCTAACGCTAATCCCCTTTCGGGGCCGCGGAAATCTTTCGATCC |
| eFx.R18 | ACCTAACGCTAATCCCCT |
| dFx.R46 | ACCTAACGCCATGTACCCTTTCGGGGATGCGGAAATCTTTCGATCC |
| dFx.R19 | ACCTAACGCCATGTACCCT |
| Ini-3'.R38 | TGGTTGCGGGGGCCGGATTTGAACCGACGATCTTCGGG |
| Ini1-1G-5'.F49 | GTAATACGACTCACTATAGGCGGGGTGGAGCAGCCTGGTAGCTCGTCGG |
| Ini-3’-OMe.R20 | TG*GTTGCGGGGGCCGGATTT |
| Ini cat.R44 | GAACCGACGATCTTCGGGTTATGAGCCCGACGAGCTACCAGGCT |
| Pro1E.F50 | GTAATACGACTCACTATAGGGTGATTGGCGCAGCCTGGTAGCGCACTTCG |
| Pro1E2-OMe.R20 | TG*GCGGGTGATAGGGGATTC |
| Pro1E2-CAU.R62 | TGGCGGGTGATAGGGGATTCGAACCCCTGACCCCTTCGCTATGAACGAAGTGCGCTACCAGG |

G* indicates 2’-O-methyl guanosine

## **Table S2.** Primers for construction of NNK and focused mRNA libraries.

| Primer | 5’-3’ sequence |
| --- | --- |
| T7g10M.F46 | TAATACGACTCACTATAGGGTTAACTTTAAGAAGGAGATATACATA |
| CGS3an13.R39 | TTTCCGCCCCCCGTCCTAGCTGCCGCTGCCGCTGCCGCA |
| NNK11CGS3.R78 | GCTGCCGCTGCCGCTGCCGCAMNNMNNMNNMNNMNNMNNMNNMNNMNNMNNMNNCATATGTATATCTCCTTCTTAAAG |
| NNK12CGS3.R81 | GCTGCCGCTGCCGCTGCCGCAMNNMNNMNNMNNMNNMNNMNNMNNMNNMNNMNNMNNCATATGTATATCTCCTTCTTAAAG |
| NNK13CGS3.R84 | GCTGCCGCTGCCGCTGCCGCAMNNMNNMNNMNNMNNMNNMNNMNNMNNMNNMNNMNNMNNCATATGTATATCTCCTTCTTAAAG |
| NNK14CGS3.R87 | GCTGCCGCTGCCGCTGCCGCAMNNMNNMNNMNNMNNMNNMNNMNNMNNMNNMNNMNNMNNMNNCATATGTATATCTCCTTCTTAAAG |
| NNK15CGS3.R90 | GCTGCCGCTGCCGCTGCCGCAMNNMNNMNNMNNMNNMNNMNNMNNMNNMNNMNNMNNMNNMNNMNNCATATGTATATCTCCTTCTTAAAG |
| HL4-focused-aromatic.R90 | GCTGCCGCTGCCGCTGCCGCAAGCATTCACMNNMNNCAGMNNMNNCGGATCATCACCAGCMNNAGACATATGTATATCTCCTTCTTAAAG |
| HL4-focused-polar.R90 | GCTGCCGCTGCCGCTGCCGCAMNNMNNMNNAAACCAMNNATAAAACGGATCMNNMNNMNNATAMNNCATATGTATATCTCCTTCTTAAAG |

## **Table S3.** Primers for mRNA library construction in site-saturation mutagenesis.

| Primer | 5’-3’ sequence |
| --- | --- |
| T7g10M.F46 | TAATACGACTCACTATAGGGTTAACTTTAAGAAGGAGATATACATA |
| SGG2an13.R36 | TTTCCGCCCCCCGTCCTAGCCTCCACTTCCACCAGA |
| HL4-ATG2-CGS3v2.F96 | GGGTTAACTTTAAGAAGGAGATATACATATGatgTATGCTGGTGATGATCCGTTTTATCTGTGGTTTGTGAATGCTTGCGGGAGTGGATCAGGTTC |
| HL4-ATG3-CGS3v2.F96 | GGGTTAACTTTAAGAAGGAGATATACATATGTCTatgGCTGGTGATGATCCGTTTTATCTGTGGTTTGTGAATGCTTGCGGGAGTGGATCAGGTTC |
| HL4-ATG4-CGS3v2.F96 | GGGTTAACTTTAAGAAGGAGATATACATATGTCTTATatgGGTGATGATCCGTTTTATCTGTGGTTTGTGAATGCTTGCGGGAGTGGATCAGGTTC |
| HL4-ATG5-CGS3v2.F96 | GGGTTAACTTTAAGAAGGAGATATACATATGTCTTATGCTatgGATGATCCGTTTTATCTGTGGTTTGTGAATGCTTGCGGGAGTGGATCAGGTTC |
| HL4-ATG6-CGS3v2.F96 | GGGTTAACTTTAAGAAGGAGATATACATATGTCTTATGCTGGTatgGATCCGTTTTATCTGTGGTTTGTGAATGCTTGCGGGAGTGGATCAGGTTC |
| HL4-ATG7-CGS3v2.F96 | GGGTTAACTTTAAGAAGGAGATATACATATGTCTTATGCTGGTGATatgCCGTTTTATCTGTGGTTTGTGAATGCTTGCGGGAGTGGATCAGGTTC |
| HL4-ATG8-CGS3v2.F96 | GGGTTAACTTTAAGAAGGAGATATACATATGTCTTATGCTGGTGATGATatgTTTTATCTGTGGTTTGTGAATGCTTGCGGGAGTGGATCAGGTTC |
| HL4-ATG9-CGS3v2.F96 | GGGTTAACTTTAAGAAGGAGATATACATATGTCTTATGCTGGTGATGATCCGatgTATCTGTGGTTTGTGAATGCTTGCGGGAGTGGATCAGGTTC |
| HL4-ATG10-CGS3v2.F96 | GGGTTAACTTTAAGAAGGAGATATACATATGTCTTATGCTGGTGATGATCCGTTTatgCTGTGGTTTGTGAATGCTTGCGGGAGTGGATCAGGTTC |
| HL4-ATG11-CGS3v2.F96 | GGGTTAACTTTAAGAAGGAGATATACATATGTCTTATGCTGGTGATGATCCGTTTTATatgTGGTTTGTGAATGCTTGCGGGAGTGGATCAGGTTC |
| HL4-ATG12-CGS3v2.F96 | GGGTTAACTTTAAGAAGGAGATATACATATGTCTTATGCTGGTGATGATCCGTTTTATCTGatgTTTGTGAATGCTTGCGGGAGTGGATCAGGTTC |
| HL4-ATG13-CGS3v2.F96 | GGGTTAACTTTAAGAAGGAGATATACATATGTCTTATGCTGGTGATGATCCGTTTTATCTGTGGatgGTGAATGCTTGCGGGAGTGGATCAGGTTC |
| HL4-ATG14-CGS3v2.F96 | GGGTTAACTTTAAGAAGGAGATATACATATGTCTTATGCTGGTGATGATCCGTTTTATCTGTGGTTTatgAATGCTTGCGGGAGTGGATCAGGTTC |
| HL4-ATG15-CGS3v2.F96 | GGGTTAACTTTAAGAAGGAGATATACATATGTCTTATGCTGGTGATGATCCGTTTTATCTGTGGTTTGTGatgGCTTGCGGGAGTGGATCAGGTTC |
| HL4-ATG16-CGS3v2.F96 | GGGTTAACTTTAAGAAGGAGATATACATATGTCTTATGCTGGTGATGATCCGTTTTATCTGTGGTTTGTGAATatgTGCGGGAGTGGATCAGGTTC |
| HL4-NNK2-CGS3v2.F96 | GGGTTAACTTTAAGAAGGAGATATACATATGnnkTATGCTGGTGATGATCCGTTTTATCTGTGGTTTGTGAATGCTTGCGGGAGTGGATCAGGTTC |
| HL4-NNK3-CGS3v2.F96 | GGGTTAACTTTAAGAAGGAGATATACATATGTCTnnkGCTGGTGATGATCCGTTTTATCTGTGGTTTGTGAATGCTTGCGGGAGTGGATCAGGTTC |
| HL4-NNK4-CGS3v2.F96 | GGGTTAACTTTAAGAAGGAGATATACATATGTCTTATnnkGGTGATGATCCGTTTTATCTGTGGTTTGTGAATGCTTGCGGGAGTGGATCAGGTTC |
| HL4-NNK5-CGS3v2.F96 | GGGTTAACTTTAAGAAGGAGATATACATATGTCTTATGCTnnkGATGATCCGTTTTATCTGTGGTTTGTGAATGCTTGCGGGAGTGGATCAGGTTC |
| HL4-NNK6-CGS3v2.F96 | GGGTTAACTTTAAGAAGGAGATATACATATGTCTTATGCTGGTnnkGATCCGTTTTATCTGTGGTTTGTGAATGCTTGCGGGAGTGGATCAGGTTC |
| HL4-NNK7-CGS3v2.F96 | GGGTTAACTTTAAGAAGGAGATATACATATGTCTTATGCTGGTGATnnkCCGTTTTATCTGTGGTTTGTGAATGCTTGCGGGAGTGGATCAGGTTC |
| HL4-NNK8-CGS3v2.F96 | GGGTTAACTTTAAGAAGGAGATATACATATGTCTTATGCTGGTGATGATnnkTTTTATCTGTGGTTTGTGAATGCTTGCGGGAGTGGATCAGGTTC |
| HL4-NNK9-CGS3v2.F96 | GGGTTAACTTTAAGAAGGAGATATACATATGTCTTATGCTGGTGATGATCCGnnkTATCTGTGGTTTGTGAATGCTTGCGGGAGTGGATCAGGTTC |
| HL4-NNK10-CGS3v2.F96 | GGGTTAACTTTAAGAAGGAGATATACATATGTCTTATGCTGGTGATGATCCGTTTnnkCTGTGGTTTGTGAATGCTTGCGGGAGTGGATCAGGTTC |
| HL4-NNK11-CGS3v2.F96 | GGGTTAACTTTAAGAAGGAGATATACATATGTCTTATGCTGGTGATGATCCGTTTTATnnkTGGTTTGTGAATGCTTGCGGGAGTGGATCAGGTTC |
| HL4-NNK12-CGS3v2.F96 | GGGTTAACTTTAAGAAGGAGATATACATATGTCTTATGCTGGTGATGATCCGTTTTATCTGnnkTTTGTGAATGCTTGCGGGAGTGGATCAGGTTC |
| HL4-NNK13-CGS3v2.F96 | GGGTTAACTTTAAGAAGGAGATATACATATGTCTTATGCTGGTGATGATCCGTTTTATCTGTGGnnkGTGAATGCTTGCGGGAGTGGATCAGGTTC |
| HL4-NNK14-CGS3v2.F96 | GGGTTAACTTTAAGAAGGAGATATACATATGTCTTATGCTGGTGATGATCCGTTTTATCTGTGGTTTnnkAATGCTTGCGGGAGTGGATCAGGTTC |
| HL4-NNK15-CGS3v2.F96 | GGGTTAACTTTAAGAAGGAGATATACATATGTCTTATGCTGGTGATGATCCGTTTTATCTGTGGTTTGTGnnkGCTTGCGGGAGTGGATCAGGTTC |
| HL4-NNK16-CGS3v2.F96 | GGGTTAACTTTAAGAAGGAGATATACATATGTCTTATGCTGGTGATGATCCGTTTTATCTGTGGTTTGTGAATnnkTGCGGGAGTGGATCAGGTTC |
| CGS3v2-HA-code1-SGG2.R90 | TTTCCGCCCCCCGTCCTAGCCTCCACTTCCACCAGACGATCCTGCGTAATCTGGAACATCGTATGGGTAAGAACCTGATCCACTCCCGCA |
| CGS3v2-HA-code2-SGG2.R90 | TTTCCGCCCCCCGTCCTAGCCTCCACTTCCACCAGAGGAACCTGCGTAATCTGGAACATCGTATGGGTAAGAACCTGATCCACTCCCGCA |
| CGS3v2-HA-code3-SGG2.R90 | TTTCCGCCCCCCGTCCTAGCCTCCACTTCCACCAGATGACCCTGCGTAATCTGGAACATCGTATGGGTAAGAACCTGATCCACTCCCGCA |
| CGS3v2-HA-code4-SGG2.R90 | TTTCCGCCCCCCGTCCTAGCCTCCACTTCCACCAGAAGAGCCTGCGTAATCTGGAACATCGTATGGGTAAGAACCTGATCCACTCCCGCA |
| CGS3v2-HA-code5-SGG2.R90 | TTTCCGCCCCCCGTCCTAGCCTCCACTTCCACCAGAACTGCCTGCGTAATCTGGAACATCGTATGGGTAAGAACCTGATCCACTCCCGCA |
| CGS3v2-HA-code6-SGG2.R90 | TTTCCGCCCCCCGTCCTAGCCTCCACTTCCACCAGAGCTACCTGCGTAATCTGGAACATCGTATGGGTAAGAACCTGATCCACTCCCGCA |
| CGS3v2-HA-code7-SGG2.R90 | TTTCCGCCCCCCGTCCTAGCCTCCACTTCCACCAGAGGATCCAGCGTAATCTGGAACATCGTATGGGTAAGAACCTGATCCACTCCCGCA |
| CGS3v2-HA-code8-SGG2.R90 | TTTCCGCCCCCCGTCCTAGCCTCCACTTCCACCAGACGAACCAGCGTAATCTGGAACATCGTATGGGTAAGAACCTGATCCACTCCCGCA |
| CGS3v2-HA-code9-SGG2.R90 | TTTCCGCCCCCCGTCCTAGCCTCCACTTCCACCAGAAGACCCAGCGTAATCTGGAACATCGTATGGGTAAGAACCTGATCCACTCCCGCA |
| CGS3v2-HA-code10-SGG2.R90 | TTTCCGCCCCCCGTCCTAGCCTCCACTTCCACCAGATGAGCCAGCGTAATCTGGAACATCGTATGGGTAAGAACCTGATCCACTCCCGCA |
| CGS3v2-HA-code11-SGG2.R90 | TTTCCGCCCCCCGTCCTAGCCTCCACTTCCACCAGAACTCCCAGCGTAATCTGGAACATCGTATGGGTAAGAACCTGATCCACTCCCGCA |
| CGS3v2-HA-code12-SGG2.R90 | TTTCCGCCCCCCGTCCTAGCCTCCACTTCCACCAGAGCTTCCAGCGTAATCTGGAACATCGTATGGGTAAGAACCTGATCCACTCCCGCA |
| CGS3v2-HA-code13-SGG2.R90 | TTTCCGCCCCCCGTCCTAGCCTCCACTTCCACCAGAAGATCCCGCGTAATCTGGAACATCGTATGGGTAAGAACCTGATCCACTCCCGCA |
| CGS3v2-HA-code14-SGG2.R90 | TTTCCGCCCCCCGTCCTAGCCTCCACTTCCACCAGATGAACCCGCGTAATCTGGAACATCGTATGGGTAAGAACCTGATCCACTCCCGCA |
| CGS3v2-HA-code15-SGG2.R90 | TTTCCGCCCCCCGTCCTAGCCTCCACTTCCACCAGAGGACCCCGCGTAATCTGGAACATCGTATGGGTAAGAACCTGATCCACTCCCGCA |
| CGS3v2-HA-code16-SGG2.R90 | TTTCCGCCCCCCGTCCTAGCCTCCACTTCCACCAGACGAGCCCGCGTAATCTGGAACATCGTATGGGTAAGAACCTGATCCACTCCCGCA |
| CGS3v2-HA-code17-SGG2.R90 | TTTCCGCCCCCCGTCCTAGCCTCCACTTCCACCAGAACTTCCCGCGTAATCTGGAACATCGTATGGGTAAGAACCTGATCCACTCCCGCA |
| CGS3v2-HA-code18-SGG2.R90 | TTTCCGCCCCCCGTCCTAGCCTCCACTTCCACCAGAGCTCCCCGCGTAATCTGGAACATCGTATGGGTAAGAACCTGATCCACTCCCGCA |
| CGS3v2-HA-code19-SGG2.R90 | TTTCCGCCCCCCGTCCTAGCCTCCACTTCCACCAGATGATCCGGCGTAATCTGGAACATCGTATGGGTAAGAACCTGATCCACTCCCGCA |
| CGS3v2-HA-code20-SGG2.R90 | TTTCCGCCCCCCGTCCTAGCCTCCACTTCCACCAGAAGAACCGGCGTAATCTGGAACATCGTATGGGTAAGAACCTGATCCACTCCCGCA |
| CGS3v2-HA-code21-SGG2.R90 | TTTCCGCCCCCCGTCCTAGCCTCCACTTCCACCAGACGACCCGGCGTAATCTGGAACATCGTATGGGTAAGAACCTGATCCACTCCCGCA |
| CGS3v2-HA-code22-SGG2.R90 | TTTCCGCCCCCCGTCCTAGCCTCCACTTCCACCAGAGGAGCCGGCGTAATCTGGAACATCGTATGGGTAAGAACCTGATCCACTCCCGCA |
| CGS3v2-HA-code23-SGG2.R90 | TTTCCGCCCCCCGTCCTAGCCTCCACTTCCACCAGAACTACCGGCGTAATCTGGAACATCGTATGGGTAAGAACCTGATCCACTCCCGCA |
| CGS3v2-HA-code24-SGG2.R90 | TTTCCGCCCCCCGTCCTAGCCTCCACTTCCACCAGAGCTGCCGGCGTAATCTGGAACATCGTATGGGTAAGAACCTGATCCACTCCCGCA |
| CGS3v2-HA-code25-SGG2.R90 | TTTCCGCCCCCCGTCCTAGCCTCCACTTCCACCAGAGGATCCTGCATAATCTGGAACATCGTATGGGTAAGAACCTGATCCACTCCCGCA |
| CGS3v2-HA-code26-SGG2.R90 | TTTCCGCCCCCCGTCCTAGCCTCCACTTCCACCAGACGAACCTGCATAATCTGGAACATCGTATGGGTAAGAACCTGATCCACTCCCGCA |
| CGS3v2-HA-code27-SGG2.R90 | TTTCCGCCCCCCGTCCTAGCCTCCACTTCCACCAGAAGACCCTGCATAATCTGGAACATCGTATGGGTAAGAACCTGATCCACTCCCGCA |
| CGS3v2-HA-code28-SGG2.R90 | TTTCCGCCCCCCGTCCTAGCCTCCACTTCCACCAGATGAGCCTGCATAATCTGGAACATCGTATGGGTAAGAACCTGATCCACTCCCGCA |
| CGS3v2-HA-code29-SGG2.R90 | TTTCCGCCCCCCGTCCTAGCCTCCACTTCCACCAGAACTCCCTGCATAATCTGGAACATCGTATGGGTAAGAACCTGATCCACTCCCGCA |
| CGS3v2-HA-code30-SGG2.R90 | TTTCCGCCCCCCGTCCTAGCCTCCACTTCCACCAGAGCTTCCTGCATAATCTGGAACATCGTATGGGTAAGAACCTGATCCACTCCCGCA |
| CGS3v2-HA-code31-SGG2.R90 | TTTCCGCCCCCCGTCCTAGCCTCCACTTCCACCAGACGATCCAGCATAATCTGGAACATCGTATGGGTAAGAACCTGATCCACTCCCGCA |
| CGS3v2-HA-code32-SGG2.R90 | TTTCCGCCCCCCGTCCTAGCCTCCACTTCCACCAGAGGAACCAGCATAATCTGGAACATCGTATGGGTAAGAACCTGATCCACTCCCGCA |
| CGS3v2-HA-code33-SGG2.R90 | TTTCCGCCCCCCGTCCTAGCCTCCACTTCCACCAGATGACCCAGCATAATCTGGAACATCGTATGGGTAAGAACCTGATCCACTCCCGCA |
| CGS3v2-HA-code34-SGG2.R90 | TTTCCGCCCCCCGTCCTAGCCTCCACTTCCACCAGAAGAGCCAGCATAATCTGGAACATCGTATGGGTAAGAACCTGATCCACTCCCGCA |
| CGS3v2-HA-code35-SGG2.R90 | TTTCCGCCCCCCGTCCTAGCCTCCACTTCCACCAGAACTGCCAGCATAATCTGGAACATCGTATGGGTAAGAACCTGATCCACTCCCGCA |
| CGS3v2-HA-code36-SGG2.R90 | TTTCCGCCCCCCGTCCTAGCCTCCACTTCCACCAGAGCTACCAGCATAATCTGGAACATCGTATGGGTAAGAACCTGATCCACTCCCGCA |
| CGS3v2-HA-code37-SGG2.R90 | TTTCCGCCCCCCGTCCTAGCCTCCACTTCCACCAGATGATCCCGCATAATCTGGAACATCGTATGGGTAAGAACCTGATCCACTCCCGCA |
| CGS3v2-HA-code38-SGG2.R90 | TTTCCGCCCCCCGTCCTAGCCTCCACTTCCACCAGAAGAACCCGCATAATCTGGAACATCGTATGGGTAAGAACCTGATCCACTCCCGCA |
| CGS3v2-HA-code39-SGG2.R90 | TTTCCGCCCCCCGTCCTAGCCTCCACTTCCACCAGACGACCCCGCATAATCTGGAACATCGTATGGGTAAGAACCTGATCCACTCCCGCA |
| CGS3v2-HA-code40-SGG2.R90 | TTTCCGCCCCCCGTCCTAGCCTCCACTTCCACCAGAGGAGCCCGCATAATCTGGAACATCGTATGGGTAAGAACCTGATCCACTCCCGCA |
| CGS3v2-HA-code41-SGG2.R90 | TTTCCGCCCCCCGTCCTAGCCTCCACTTCCACCAGAACTACCCGCATAATCTGGAACATCGTATGGGTAAGAACCTGATCCACTCCCGCA |
| CGS3v2-HA-code42-SGG2.R90 | TTTCCGCCCCCCGTCCTAGCCTCCACTTCCACCAGAGCTGCCCGCATAATCTGGAACATCGTATGGGTAAGAACCTGATCCACTCCCGCA |
| CGS3v2-HA-code43-SGG2.R90 | TTTCCGCCCCCCGTCCTAGCCTCCACTTCCACCAGAAGATCCGGCATAATCTGGAACATCGTATGGGTAAGAACCTGATCCACTCCCGCA |
| CGS3v2-HA-code44-SGG2.R90 | TTTCCGCCCCCCGTCCTAGCCTCCACTTCCACCAGATGAACCGGCATAATCTGGAACATCGTATGGGTAAGAACCTGATCCACTCCCGCA |
| CGS3v2-HA-code45-SGG2.R90 | TTTCCGCCCCCCGTCCTAGCCTCCACTTCCACCAGAGGACCCGGCATAATCTGGAACATCGTATGGGTAAGAACCTGATCCACTCCCGCA |
| CGS3v2-HA-code46-SGG2.R90 | TTTCCGCCCCCCGTCCTAGCCTCCACTTCCACCAGACGAGCCGGCATAATCTGGAACATCGTATGGGTAAGAACCTGATCCACTCCCGCA |
| CGS3v2-HA-code47-SGG2.R90 | TTTCCGCCCCCCGTCCTAGCCTCCACTTCCACCAGAACTTCCGGCATAATCTGGAACATCGTATGGGTAAGAACCTGATCCACTCCCGCA |
| CGS3v2-HA-code48-SGG2.R90 | TTTCCGCCCCCCGTCCTAGCCTCCACTTCCACCAGAGCTCCCGGCATAATCTGGAACATCGTATGGGTAAGAACCTGATCCACTCCCGCA |

The nucleotides in lowercase letters indicate the scanning positions. The 48 barcodes encoded for site-saturation mutagenesis are highlighted in red.

## **Table S4.** Primers for RaPID screening and NGS.

| Primer | 5’-3’ sequence |
| --- | --- |
| T7g10M.F46 | TAATACGACTCACTATAGGGTTAACTTTAAGAAGGAGATATACATA |
| CGS3an13.R39 | TTTCCGCCCCCCGTCCTAGCTGCCGCTGCCGCTGCCGCA |
| Rd1T7g10M.F70 | CACTCTTTCCCTACACGACGCTCTTCCGATCTTAATACGACTCACTATAGGGTTAACTTTAAGAAGGAGA |
| an13Rd2.R49 | GACTGGAGTTCAGACGTGTGCTCTTCCGATCTTTTCCGCCCCCCGTCCT |
| P5S502Rd1.F57 | AATGATACGGCGACCACCGAGATCTACACCTCTCTATACACTCTTTCCCTACACGAC |
| P5S503Rd1.F57 | AATGATACGGCGACCACCGAGATCTACACTATCCTCTACACTCTTTCCCTACACGAC |
| P5S505Rd1.F57 | AATGATACGGCGACCACCGAGATCTACACGTAAGGAGACACTCTTTCCCTACACGAC |
| P5S506Rd1.F57 | AATGATACGGCGACCACCGAGATCTACACACTGCATAACACTCTTTCCCTACACGAC |
| P5S507Rd1.F57 | AATGATACGGCGACCACCGAGATCTACACAAGGAGTAACACTCTTTCCCTACACGAC |
| P5S508Rd1.F57 | AATGATACGGCGACCACCGAGATCTACACCTAAGCCTACACTCTTTCCCTACACGAC |
| P5S510Rd1.F57 | AATGATACGGCGACCACCGAGATCTACACCGTCTAATACACTCTTTCCCTACACGAC |
| P5S511Rd1.F57 | AATGATACGGCGACCACCGAGATCTACACTCTCTCCGACACTCTTTCCCTACACGAC |
| Rd2N701P7.R52 | CAAGCAGAAGACGGCATACGAGATTCGCCTTAGTGACTGGAGTTCAGACGTG |
| Rd2N702P7.R52 | CAAGCAGAAGACGGCATACGAGATCTAGTACGGTGACTGGAGTTCAGACGTG |
| Rd2N703P7.R52 | CAAGCAGAAGACGGCATACGAGATTTCTGCCTGTGACTGGAGTTCAGACGTG |
| Rd2N704P7.R52 | CAAGCAGAAGACGGCATACGAGATGCTCAGGAGTGACTGGAGTTCAGACGTG |
| Rd2N705P7.R52 | CAAGCAGAAGACGGCATACGAGATAGGAGTCCGTGACTGGAGTTCAGACGTG |
| Rd2N706P7.R52 | CAAGCAGAAGACGGCATACGAGATCATGCCTAGTGACTGGAGTTCAGACGTG |
| Rd2N707P7.R52 | CAAGCAGAAGACGGCATACGAGATGTAGAGAGGTGACTGGAGTTCAGACGTG |
| Rd2N710P7.R52 | CAAGCAGAAGACGGCATACGAGATCAGCCTCGGTGACTGGAGTTCAGACGTG |
| Rd2N711P7.R52 | CAAGCAGAAGACGGCATACGAGATTGCCTCTTGTGACTGGAGTTCAGACGTG |
| Rd2N712P7.R52 | CAAGCAGAAGACGGCATACGAGATTCCTCTACGTGACTGGAGTTCAGACGTG |
| Rd2N714P7.R52 | CAAGCAGAAGACGGCATACGAGATTCATGAGCGTGACTGGAGTTCAGACGTG |
| Rd2N715P7.R52 | CAAGCAGAAGACGGCATACGAGATCCTGAGATGTGACTGGAGTTCAGACGTG |
| Rd2N716P7.R52 | CAAGCAGAAGACGGCATACGAGATTAGCGAGTGTGACTGGAGTTCAGACGTG |
| Rd2N718P7.R52 | CAAGCAGAAGACGGCATACGAGATGTAGCTCCGTGACTGGAGTTCAGACGTG |
| Rd2N719P7.R52 | CAAGCAGAAGACGGCATACGAGATTACTACGCGTGACTGGAGTTCAGACGTG |
| Rd2N720P7.R52 | CAAGCAGAAGACGGCATACGAGATAGGCTCCGGTGACTGGAGTTCAGACGTG |
| Rd2N721P7.R52 | CAAGCAGAAGACGGCATACGAGATGCAGCGTAGTGACTGGAGTTCAGACGTG |
| Rd2N722P7.R52 | CAAGCAGAAGACGGCATACGAGATCTGCGCATGTGACTGGAGTTCAGACGTG |
| Rd2N723P7.R52 | CAAGCAGAAGACGGCATACGAGATGAGCGCTAGTGACTGGAGTTCAGACGTG |
| Rd2N724P7.R52 | CAAGCAGAAGACGGCATACGAGATCGCTCAGTGTGACTGGAGTTCAGACGTG |
| Rd2N726P7.R52 | CAAGCAGAAGACGGCATACGAGATGTCTTAGGGTGACTGGAGTTCAGACGTG |
| Rd2N727P7.R52 | CAAGCAGAAGACGGCATACGAGATACTGATCGGTGACTGGAGTTCAGACGTG |
| Rd2N728P7.R52 | CAAGCAGAAGACGGCATACGAGATTAGCTGCAGTGACTGGAGTTCAGACGTG |
| Rd2N729P7.R52 | CAAGCAGAAGACGGCATACGAGATGACGTCGAGTGACTGGAGTTCAGACGTG |

## **Table S5.** Aminoacylation of tRNAs with non-proteinogenic amino acids.

| Token | Amino acid | tRNA | Activated ester /flexizyme | Incubation time (h) |
| --- | --- | --- | --- | --- |
|  | *N*-chloroacetyl-L-tyrosine | tRNA^fMet^_CAU_ | CME/eFx | 2 |
| A1 | L-2-aminobutyric acid | tRNA^Pro1E2^_CAU_ | DBE/dFx | 2 |
| A2 | L-norvaline | tRNA^Pro1E2^_CAU_ | DBE/dFx | 2 |
| A3 | L-norisoleucine | tRNA^Pro1E2^_CAU_ | DBE/dFx | 10 |
| A4 | L-2-aminoheptanoic acid | tRNA^Pro1E2^_CAU_ | DBE/dFx | 6 |
| A5 | L-2-aminooctanoic acid | tRNA^Pro1E2^_CAU_ | DBE/dFx | 6 |
| A6 | L-g-methyl-leucine | tRNA^Pro1E2^_CAU_ | DBE/dFx | 6 |
| A7 | cyclopentyl-L-alanine | tRNA^Pro1E2^_CAU_ | DBE/dFx | 6 |
| A8 | cyclohexyl-L-alanine | tRNA^Pro1E2^_CAU_ | DBE/dFx | 6 |
| A9 | 2-aminoisobutyric acid | tRNA^Pro1E2^_CAU_ | DBE/dFx | 6 |
| A10 | (S)-4,5-dehydroleucine | tRNA^Pro1E2^_CAU_ | DBE/dFx | 6 |
| M1 | *N*-methyl-glycine | tRNA^Pro1E2^_CAU_ | DBE/dFx | 6 |
| M2 | *N*-methyl-L-phenylalanine | tRNA^Pro1E2^_CAU_ | CME/eFx | 6 |
| M3 | *N*-methyl-L- tyrosine | tRNA^Pro1E2^_CAU_ | CME/eFx | 6 |
| D1 | D-alanine | tRNA^Pro1E2^_CAU_ | DBE/dFx | 6 |
| D2 | D-phenylalanine | tRNA^Pro1E2^_CAU_ | CME/eFx | 6 |
| D3 | D-tyrosine | tRNA^Pro1E2^_CAU_ | CME/eFx | 6 |
| N1 | *N*-ethyl-glycine | tRNA^Pro1E2^_CAU_ | DBE/dFx | 6 |
| N2 | *N*-propyl-glycine | tRNA^Pro1E2^_CAU_ | DBE/dFx | 6 |
| N3 | *N*-butyl-glycine | tRNA^Pro1E2^_CAU_ | DBE/dFx | 6 |
| N4 | *N*-pentyl-glycine | tRNA^Pro1E2^_CAU_ | DBE/dFx | 6 |
| N5 | *N*-isopentyl-glycine | tRNA^Pro1E2^_CAU_ | DBE/dFx | 6 |
| N6 | *N*-hexyl-glycine | tRNA^Pro1E2^_CAU_ | DBE/dFx | 6 |
| N7 | *N*-phenethyl-glycine | tRNA^Pro1E2^_CAU_ | DBE/dFx | 6 |
| P1 | *trans*-4-hydroxy-L-proline | tRNA^Pro1E2^_CAU_ | DBE/dFx | 6 |
| P2 | *cis*-4-hydroxy-L-proline | tRNA^Pro1E2^_CAU_ | DBE/dFx | 6 |
| P3 | L-pipecolic acid | tRNA^Pro1E2^_CAU_ | DBE/dFx | 6 |
| P4 | L-1,2,3,4-tetrahydroisoquinoline-3-carboxylic acid | tRNA^Pro1E2^_CAU_ | CME /dFx | 6 |
| O1 | *O*-methyl-L-serine | tRNA^Pro1E2^_CAU_ | DBE/dFx | 6 |
| O2 | *O*-methyl-L-threonine | tRNA^Pro1E2^_CAU_ | DBE/dFx | 6 |
| O3 | *O*-methyl-L-tyrosine | tRNA^Pro1E2^_CAU_ | CME/eFx | 6 |
| O4 | **(2S)-2-Amino-4-methoxy-4-oxobutanoic acid** | tRNA^Pro1E2^_CAU_ | DBE/dFx | 6 |
| O5 | **(2S)-2-Amino-5-methoxy-5-oxopentanoic acid** | tRNA^Pro1E2^_CAU_ | DBE/dFx | 6 |
| K1 | L-β-ureidoalanine | tRNA^Pro1E2^_CAU_ | DBE/dFx | 6 |
| K2 | L-2-amino-5-ureidovaleric acid | tRNA^Pro1E2^_CAU_ | DBE/dFx | 6 |
| K3 | L-**homocitrulline** | tRNA^Pro1E2^_CAU_ | DBE/dFx | 6 |
| R1 | **4-fluoro-**L**-phenylalanine** | tRNA^Pro1E2^_CAU_ | CME/eFx | 8 |
| R2 | **2,3,4,5,6-pentafluoro-**L**-phenylalanine** | tRNA^Pro1E2^_CAU_ | CME/eFx | 8 |
| R3 | **4-(trifluoromethyl)-**L**-phenylalanine** | tRNA^Pro1E2^_CAU_ | CME/eFx | 8 |
| R4 | ***O*-(trifluoromethyl)-**L**-tyrosine** | tRNA^Pro1E2^_CAU_ | CME/eFx | 8 |
| R5 | **3-chloro-**L**-phenylalanine** | tRNA^Pro1E2^_CAU_ | CME/eFx | 8 |
| R6 | **4-chloro-**L**-phenylalanine** | tRNA^Pro1E2^_CAU_ | CME/eFx | 8 |
| R7 | (1-naphthyl)-L-alanine | tRNA^Pro1E2^_CAU_ | CME/eFx | 8 |
| R8 | (2-naphthyl)-L-alanine | tRNA^Pro1E2^_CAU_ | CME/eFx | 8 |
| R9 | **3-(1-benzothiophen-3-yl)-**L**-alanine** | tRNA^Pro1E2^_CAU_ | CME/eFx | 8 |
| R10 | (2-thienyl)-L-alanine | tRNA^Pro1E2^_CAU_ | CME/eFx | 8 |
| R11 | (3-thienyl)-L-alanine | tRNA^Pro1E2^_CAU_ | CME/eFx | 8 |
| R12 | 3-(1,3-thiazol-4-yl)-L-alanine | tRNA^Pro1E2^_CAU_ | CME/eFx | 8 |
